# Inclusion of underserved young people in digital public health interventions for mental health, sexual health and substance use: A systematic review and intervention component analysis

**DOI:** 10.1016/j.invent.2026.100985

**Published:** 2026-08-06

**Authors:** Claire Tatton, Joelle Kirby, Jessica M. Armitage, Sophie Robinson, Rabeea'h Waseem Aslam, Tom Arthur, Sean Harrison, Daniel Mutanda, Ruth Garside, Jo Thompson Coon, Rhiannon Evans, G.J. Melendez-Torres

**Affiliations:** a Faculty of Health and Life Sciences, University of Exeter, UK; b DECIPHer, School of Social Sciences, Cardiff University, Cardiff, UK; c NIHR Applied Research Collaboration South West Peninsula (PenARC), UK

**Keywords:** Digital inclusion, Young people, Public health, Underserved groups

## Abstract

**Objective:**

This review examined the design features of digital public health interventions for mental health, sexual health and substance use that may affect equitable reach, engagement, and effectiveness for underserved young people.

**Materials and methods:**

We searched MEDLINE, Embase and PsycINFO for relevant systematic reviews published since 2020. We assessed them using the Peer Review of Electronic Search Strategies guidelines. We extracted randomised trials from six reviews and used them to inform searches in PsycINFO and CENTRAL for additional trials. We included mental health, sexual health and substance use interventions delivered solely or primarily through digital technologies to those aged 13–25 from underserved groups.

**Results:**

We included 62 trials of 56 interventions. Thirty-eight were sexual health interventions. We identified four coproduction components and six delivery components which may engage young people with interventions and turn knowledge into action. Evidence of effectiveness was strongest for substance use, with mixed or non-significant effects across other behavioural and biological outcomes.

**Discussion:**

Similar delivery components were evidenced across all outcome domains and underserved groups, indicating potential synergies in the ways interventions may help young people to navigate these interlinked issues. Coproduction was not well evidenced outside of sexual health interventions and those for sexual and ethnic minoritised youth.

**Conclusion:**

Whilst there is some indication of effectiveness for tailored interventions, further evaluations across a broader range of underserved groups are needed. Our framework supports an understanding of how tailored interventions may work for underserved young people that may inform future intervention design and evaluation.

## Highlights

• Digital public health interventions have been tailored to underserved young people.
• A component framework included coproduction and six delivery components.
• Effectiveness evidence was strongest for substance use.
• Coproduction was not generally well evidenced outside of sexual health.
• Effectiveness was limited for biological sexual health outcomes.

## 1 Introduction

Young people worldwide face increased risks in relation to mental ill-health, poor sexual health and the misuse of substances (World Health Organisation, 2024). These issues are often interconnected affecting resilience, decision making, and risk taking (World Health Organisation, 2025). To address this, integrated or parallel interventions are required that provide accessible mental health support (Colizzi et al., 2020), substance misuse education, and an open dialogue around sexual health (Guse et al., 2012).

Digital health interventions are widely regarded as having the potential to improve accessibility to care and support through enhanced efficiency and personalisation (Hollis et al., 2015). Digital interventions for public health have contrasting definitions across existing literature and guidance. Broadly, they refer to those provided electronically in a health care system, formally or informally (Iyamu et al., 2021; Soobiah et al., 2020). Digital public health (PH) interventions cover individual-level prevention interventions delivered via portable devices (mHealth), use of digital information and communications to deliver healthcare services to individuals and populations (eHealth), and population-level health promotion and management tools (digital health) (Wienert et al., 2022). Importantly, their focus is on prevention and health promotion (Wienert et al., 2022).

While young people's extensive use of digital technology suggests a readiness to engage with digital interventions, this assumption can drive their development without sufficient empirical validation of their aetiology (Baumel et al., 2019; Ramshaw et al., 2023). Systematic reviews have examined digital interventions for young people, yet, effective approaches that address the interconnected issues of mental ill-health, sexual ill-health, and substance misuse, are hard to identity (Hollis et al., 2017). This is especially challenging for underserved groups who experience greater barriers to access support, or because of failures to understand and provide to the different ways they may engage with and respond to interventions (NIHR, 2020). Disparities in digital literacy, access to the required technologies, and lack of cultural relevance may lead to a deepening of the digital divide (Piers et al., 2023). Consequently, interventions designed for general populations may inadvertently exacerbate existing health inequities by advantaging who already possess technological and socioeconomic advantages (Whitehead et al., 2024).

To address these gaps, we undertook this review to explore the design features of tailored digital PH interventions that may affect equitable reach, engagement and effectiveness for underserved groups. We defined digital PH interventions as those that solely or primarily deliver content using digital technologies, and those for universal prevention (for general population), selective prevention (for higher risk sub-groups), or indicated prevention (for individuals with increased vulnerability) (O'Connell and Warner, 2009), but not for treatment or disease management. We used Intervention Component Analysis because it offers a pragmatic approach to understanding the critical features of complex interventions (Sutcliffe et al., 2015) and therefore to identifying equity relevant components that may reduce disparities.

## 2 Methods

We systematically reviewed eligible randomised trials using Intervention Component Analysis (Sutcliffe et al., 2015) and principles from Grounded Theory (Lewis-Beck et al., 2004) to answer:

• What is the evidence of effectiveness of tailored interventions on behavioural and biological outcomes?
• How are digital PH interventions for mental health, sexual health and substance use tailored to underserved young people?

We defined underserved groups using the PROGRESS-Plus heuristic (O'Neill et al., 2014) and referred to the PRO EDI guidance for considering equity, diversity, and inclusion in evidence reviews (Trial Forge, E.S.I., Campbell Collaboration, 2024). The review was registered with PROSPERO CRD42025641364. Ethics approval was not required.

### 2.1 Search methods

As digital health interventions have been extensively researched, we conducted the search for relevant randomised trials in two phases to maximise coverage and efficiency. First, we searched MEDLINE, Embase and PsycINFO for systematic reviews published since 2020, reflecting the fast-moving field of digital health. We designed the search strategy to retrieve trials of digital interventions (including associated platforms, devices, software and individual interventions) that examined the three outcome domains. We derived terms from existing strategies in relevant systematic reviews and refined them via iterative explorative searching to improve precision. We applied a systematic review filter (SR / MA / HTA / ITC (Canada Drug Agency, 2026)) to searches in MEDLINE and PsycINFO. The retrieved reviews were broad in scope as we did not limit searches by specific intervention modality or equity relevant groups. Using principles from the Peer Review of Electronic Search Strategies guideline (McGowan et al., 2016), an experienced information specialist (JK) reviewed the search strategy and supplemental search methods of each review to determine how likely each was to have missed relevant studies. Assessments were discussed with three reviewers (GJMT, TA, CT) who together determined the two reviews for each outcome domain which were most likely to contain relevant studies for this review. We extracted relevant randomised trials from these priority reviews and used them to refine the search strategy for the second search. For this, we searched PsycINFO and CENTRAL (to search across sources including Medline and Embase). The search strategies are presented in the supplementary file.

### 2.2 Eligibility criteria

We included only randomised trials of digital PH interventions for mental health, sexual health and substance use conducted in high income countries (defined by the World Bank (World Bank, 2026)) delivered to young people aged 13 to 25 years from an underserved group (defined using the PROGRESS-Plus heuristic (O'Neill et al., 2014)). We excluded trials undertaken in diagnostic or clinical mental health populations (which tended to focus on treatment), populations with an existing health condition, and populations with an addiction disorder (except nicotine or tobacco addiction). We excluded trials undertaken in higher education settings which only reported substance use outcomes as most of this evidence was from the USA which has a higher legal drinking age than most other high-income countries. We did not apply any limitations to language.

We included universal, selective, and indicated prevention interventions that solely or primarily used digital technologies to deliver intervention content (for example, by text messages, social media, and via web-based platforms). We excluded interventions that used digital technologies solely for measurement or assessment (for example, wearables, pedometers) without linked content delivery, and interventions of one-time passive delivery of information (for example, a single video or static webpage). We also excluded interventions designed for online provision of clinical services, for individual management of health services (for example, telehealth/telemedicine), and digital interventions that required substantial therapist or face-to-face contact (beyond set-up support or safety). We included any comparator.

We included interventions which measured depression, anxiety, mental wellbeing, suicide risk or self-harm outcomes, sexually transmitted infections, sexual risk behaviours, uptake of contraception or protective technologies (for example, barrier methods, pre-exposure prophylaxis (PrEP)), alcohol, tobacco, or other drug use outcomes. Full eligibility criteria are presented in the supplementary file.

### 2.3 Screening

Five reviewers (JK, RWA, TA, CT, GJMT) screened the systematic reviews, and three reviewers (JMA, CT, GJMT) screened the randomised trials using EPPI reviewer. All screening was done in duplicate and independently. Conflicts in assessments were resolved through discussion and recourse to a third reviewer, as required.

### 2.4 Data extraction and analysis

One reviewer (RWA) extracted data items related to trial and intervention design, and outcomes. Data extraction was checked by a second reviewer (CT). One reviewer (RWA) appraised the quality of included trials using the Cochrane Risk of Bias tool (Higgins, 2011), selected due to its suitability for assessing study level bias. Risk of bias assessments were audited by a second reviewer (CT). We summarised judgements of risk of bias using the framework developed by the Cochrane Collaboration (Higgins et al., 2011). We coded underserved groups by their focal characteristic (defined by the trial eligibility criteria or the focus of the paper) and noted other equity relevant characteristics of interest present in over half of the trial sample. We categorised interventions according to their main outcome domain. Due to heterogeneity, we were unable to conduct meta-analyses of intervention effectiveness. We constructed harvest plots to depict the weight and direction of the available evidence for the range of outcomes of interest. We plotted all relevant outcomes from trials measured across a range of timepoints. We categorised these are short term (< 3 months post intervention), medium term (4–12 months post intervention) and long term (>12 months post intervention). Where available, we used adjusted outcome measures over unadjusted measures. We also included information on trial sample size, risk of bias assessment and equity tailoring. We used a *p* value of 0.05 to indicate statistical significance (Higgins et al., 2011).

One reviewer (JMA) summarised intervention descriptions from the main trial reports and their supplementary materials. Three reviewers (JMA, CT, GJMT) began by selecting five intervention descriptions relating to a range of underserved groups. Each reviewer independently used open coding (Benaquisto, 2008) to generate a list of possible intervention descriptors, focusing on aspects developed to meet the needs of underserved groups. We compared and combined the lists. Using principles of axial coding (Simmons, 2017), we proceeded through the remaining intervention descriptions, collapsing codes, adding new codes, organising codes into categories and identifying relationships between the components. This resulted in a comprehensive list of descriptors to characterise the equity relevant components of included interventions. For simplicity, we associated intervention components to the focal equity relevant characteristic and main outcome domain.

## 3 Results

**Fig. 1:**
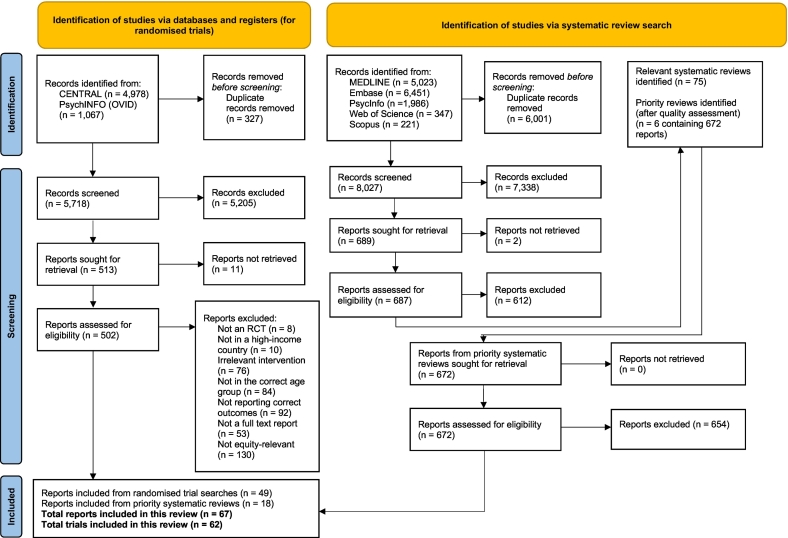
PRISMA flowchart.

A total of 62 trials of 56 tailored interventions reported in 67 papers were included in this review. Of the 67 papers included, 18 were identified through the priority systematic reviews and 49 were identified through searches for randomised trials (Fig. 1).

### 3.1 Characteristics of included trials and interventions

Fifty-five trials were conducted in North America, four in Europe, and three in Asia. Thirty-eight trials were of sexual health interventions (Bauermeister et al., 2024; Bauermeister et al., 2019; Bennett et al., 2015; Biello et al., 2022; Biello et al., 2020; Biello et al., 2025; Bull et al., 2016; Bull et al., 2012; Carvalho et al., 2016; Chernick et al., 2025; Chernick et al., 2022; Chernick et al., 2017; Christensen et al., 2013; Cordova et al., 2020; Fiellin et al., 2017; Gerend et al., 2020; Hebert et al., 2018; Hightow-Weidman et al., 2019; Jones et al., 2013; Kaufman et al., 2025; Kissinger et al., 2023; Manlove et al., 2020; Martínez-García et al., 2023; Mustanski et al., 2013; Mustanski et al., 2025; Mustanski et al., 2018; Patel et al., 2024; Reback et al., 2024; Reiter et al., 2023; Santa Maria et al., 2021; Schnall et al., 2022; Schnall et al., 2024; Starosta et al., 2016; Stephenson et al., 2020; Tan et al., 2022; Whiteley et al., 2018; Ybarra et al., 2021; Ybarra et al., 2017), fifteen were of substance use interventions (Fang et al., 2010; Gilmore et al., 2023; Mason et al., 2016; Mason et al., 2015; Prokhorov et al., 2008; Schinke et al., 2009; Schinke et al., 2005; Schwartz et al., 2014; Schwinn et al., 2021; Schwinn et al., 2010; Schwinn et al., 2015; Wall et al., 2024; Walton et al., 2013; Walton et al., 2010; Walton et al., 2014), and nine were of mental health interventions (Aboody et al., 2020; Ampatzoglou et al., 2024; Bauermeister et al., 2022; Craig Rushing et al., 2021; Egan et al., 2021; Glass et al., 2024; Glass et al., 2022; Jeong et al., 2024; Shen et al., 2023).

The number of tailored interventions by main outcome domain and focal equity relevant characteristic are presented in Table 1. Trial, population and intervention details are reported in the supplementary file.

**Table 1:** Interventions by main outcome domain and focal equity relevant characteristic.

	Main outcome domain			
Focal PROGRESS-Plus characteristic	Sexual health	Substance use	Mental health	TOTAL
Place of residence	5	6	0	11
Race/ethnicity/culture/language	9	4	1	14
Gender/sex	7	3	4	14
Personal characteristics	17	1	3	21
Features of relationships	0	1	1	2
TOTAL	38	15	9	62

Ten interventions tailored to place focussed on urban and inner-city locations underserved by healthcare services or with large proportions of ethnic minoritised and disadvantaged young people (Chernick et al., 2025; Chernick et al., 2022; Chernick et al., 2017; Cordova et al., 2020; Gilmore et al., 2023; Prokhorov et al., 2008; Schinke et al., 2005; Walton et al., 2013; Walton et al., 2010; Walton et al., 2014). One focussed on homeless young people (Santa Maria et al., 2021).

Two interventions tailored to ethnicity focussed on American Indian and Alaska Native youth (Kaufman et al., 2025; Craig Rushing et al., 2021) and twelve on African American, Hispanic, Latinx and Latina youth (Bull et al., 2016; Bull et al., 2012; Fiellin et al., 2017; Hebert et al., 2018; Jones et al., 2013; Kissinger et al., 2023; Manlove et al., 2020; Whiteley et al., 2018; Fang et al., 2010; Mason et al., 2016; Mason et al., 2015; Schwinn et al., 2021).

Twelve interventions tailored to gender/sex focussed on girls (Bennett et al., 2015; Martínez-García et al., 2023; Patel et al., 2024; Reback et al., 2024; Starosta et al., 2016; Stephenson et al., 2020; Schinke et al., 2009; Schwartz et al., 2014; Schwinn et al., 2010; Aboody et al., 2020; Ampatzoglou et al., 2024; Glass et al., 2022; Jeong et al., 2024), one on transgender and gender expansive youth (Reback et al., 2024) and one to boys (Carvalho et al., 2016).

Sixteen interventions tailored to personal characteristics focussed on young men who have sex with men (YMSM) (Bauermeister et al., 2024; Bauermeister et al., 2019; Biello et al., 2022; Biello et al., 2020; Biello et al., 2025; Christensen et al., 2013; Gerend et al., 2020; Hightow-Weidman et al., 2019; Mustanski et al., 2013; Mustanski et al., 2025; Mustanski et al., 2018; Reiter et al., 2023; Schnall et al., 2022; Schnall et al., 2024; Tan et al., 2022; Ybarra et al., 2017) and five on sexual and gender minority youth (Ybarra et al., 2021; Schwinn et al., 2015; Bauermeister et al., 2022; Egan et al., 2021; Shen et al., 2023).

Two interventions tailored to features of relationships focussed on youth with parents with alcohol use problems (Wall et al., 2024), and youth with experience of intimate partner violence (IPV) (Glass et al., 2024).

No interventions were focally tailored to occupation, religion, education, socioeconomic status, social capital or time-dependent relationships.

### 3.2 Effectiveness

#### 3.2.1 Sexual health

**Fig. 2:**
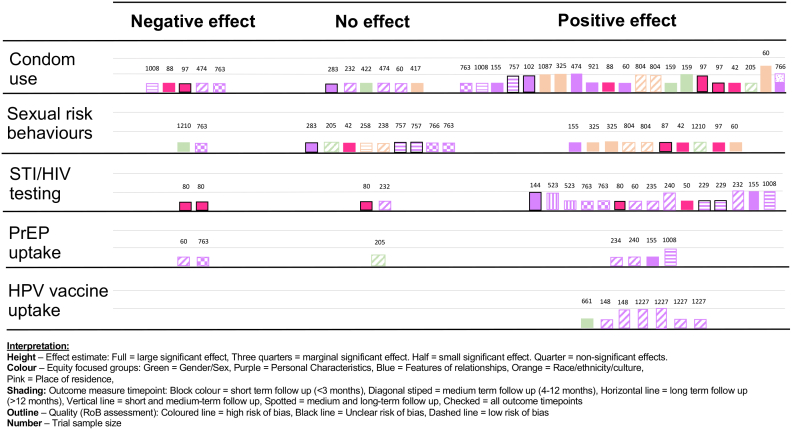
Harvest plots - sexual health outcomes I.

**Fig. 3:**
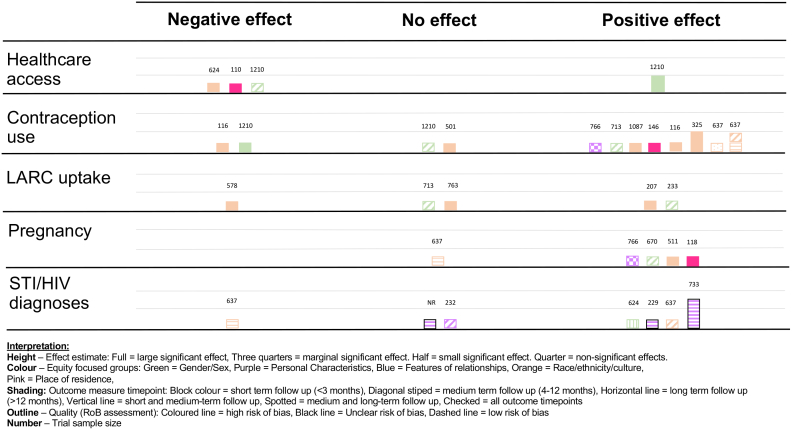
Harvest plots - sexual health outcomes II.

Overall, there was some indication of a positive gradient of effects for improved condom use. This was driven by a mix of significant and non-significant positive effects, mainly at short term follow up. There was some indication that mixed effects were observed more frequently in interventions tailored to personal characteristics and place of residence than in interventions tailored to race/ethnicity and gender/sex. Findings are summarised in Fig. 2, Fig. 3.

There was no indication of a gradient of effectiveness for reduction in sexual risk behaviours tailored to place of residence, personal characteristics, race/ethnicity and gender/sex, mainly at short and medium term follow up.

Taking together outcomes for improving STI/HIV testing, evidence indicated a positive gradient of effects. This was mainly driven through interventions tailored to personal characteristics across a range of follow up timepoints. Despite the overall positive direction, effects for interventions tailored to place of residence appeared mixed.

There was some indication of a gradient of effectiveness for improved uptake of pre-exposure prophylaxis (PrEP)). This was driven mainly through interventions tailored to personal characteristics. However, in most trials, the positive effects did not reach statistical significance.

Overall, trials of interventions for to improve the human papilloma virus (HPV) vaccine rates indicated a positive gradient of effects. This was driven mainly through interventions tailored to personal characteristics through medium term outcome measures. Statistically significant effects were indicated for vaccine uptake, but not for completion (Gerend et al., 2020; Reiter et al., 2023).

On balance, there was no indication of a gradient of effectiveness for improving healthcare access or uptake of long-acting reversible contraception (LARC) uptake in interventions tailored to race/ethnicity, gender/sex and place of residence.

There was some indication of a positive gradient of effects for improved contraception use (other than condom use or LARC) and for reducing pregnancies. These were predominantly driven from interventions tailored to race/ethnicity and gender/sex across a range of timepoints. However, in most trials, effects did not reach statistical significance.

Finally, there was some indication of a possible positive gradient to effects for improved STI/HIV diagnoses in interventions tailored to gender/sex, place of residence or race/ethnicity from short and medium term outcome data. However, again, in most trials, effects did not reach statistical significance.

Five trials were assessed as unclear risk of bias (Bauermeister et al., 2024; Chernick et al., 2025; Mustanski et al., 2013; Mustanski et al., 2018; Ybarra et al., 2017) and the remainder were assessed as high risk of bias.

#### 3.2.2 Substance use

**Fig. 4:**
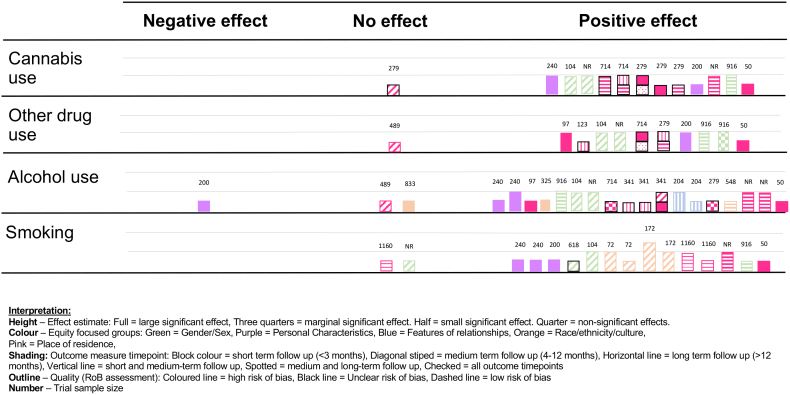
Harvest plot - substance use outcomes.

Taken together, trials indicated a direction of statistically significant positive effects on cannabis and other drug use outcomes in interventions tailored to place of residence, gender/sex and personal characteristics, mainly driven by medium and long term outcome data. Findings are summarised in Fig. 4.

Also, trials indicated a direction of positive effects on alcohol use outcomes in interventions tailored to place of residence, gender/sex, race/ethnicity and features of relationships, driven mainly by medium and long term follow up data. However, positive effects frequently did not reach statistical significance. Whilst evidence indicated a general positive direction in reducing alcohol use, no trials reported improved rates of abstinence.

Finally, trials indicated a direction of positive effects on smoking outcomes in interventions tailored to personal characteristics, gender/sex, race/ethnicity, place of residence and personal characteristics, mainly driven from medium and long term outcome data. However, positive effects frequently did not reach statistical significance. Whilst evidence indicated a general positive direction on smoking prevention and reduction, no trials reported improved rates of cessation.

Five trials were assessed as unclear risk of bias (Gilmore et al., 2023; Schwartz et al., 2014; Walton et al., 2013; Walton et al., 2010; Walton et al., 2014), the remainder were rated as high risk of bias.

#### 3.2.3 Mental health

**Fig. 5:**
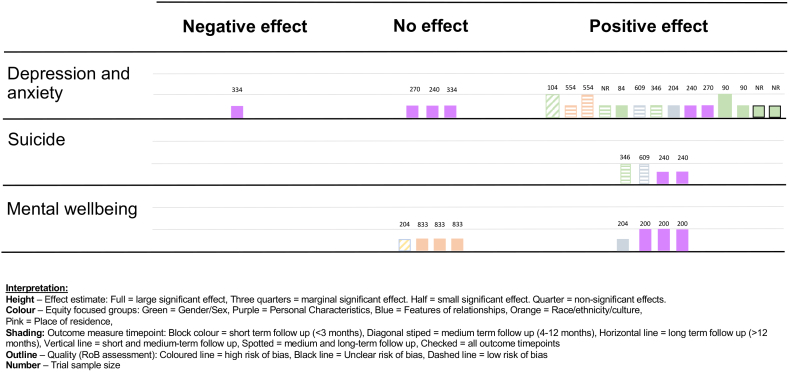
Harvest plot - mental health outcomes.

Taking together outcomes for depression and anxiety, evidence indicated a positive gradient of effects driven mainly by short and long term measures. However, positive effects frequently did not reach statistical significance. There was some indication that effects of interventions tailored to personal characteristics were more mixed than for interventions tailored to race/ethnicity, gender/sex and features of relationships. Findings are summarised in Fig. 5.

Overall, trials of interventions to reduce suicide risk indicated a positive gradient of effects. However, only four trials of interventions were included tailored to gender/sex, personal characteristics and features of relationships. Statistically significant outcomes were only observed in long term outcome measures.

Finally, on balance, there was no indication of a gradient of effectiveness for mental wellbeing outcomes in interventions tailored to race/ethnicity, personal characteristics and features of relationships.

One trial was assessed as unclear risk of bias (Ampatzoglou et al., 2024), the remainder were rated as high risk of bias.

Trial level outcomes and risk of bias assessments are presented in the supplementary materials. The main sources of bias across trials across all outcome domains were lack of blinding of participants, personnel, and outcome assessors, and incomplete outcome data.

#### 3.2.4 Intervention component framework

**Fig. 6:**
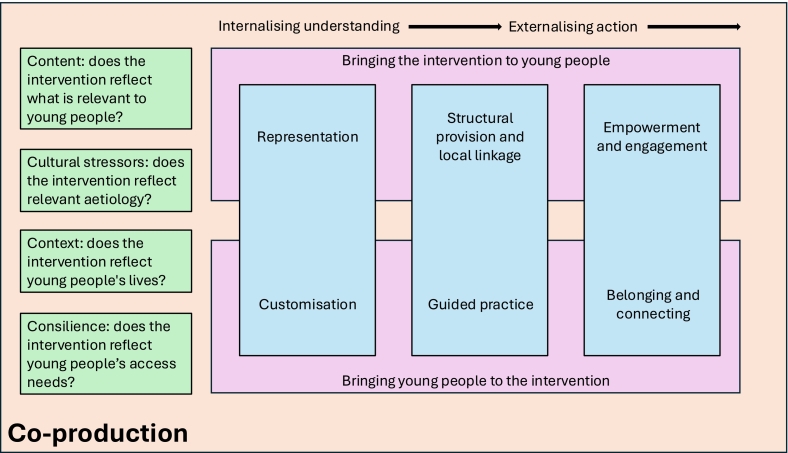
Framework for equity tailoring components.

We structured our list of equity relevant components designed to meet the needs of underserved groups into four coproduction components and six delivery components. We arranged the delivery components into three sets of dyads that work in parallel to bring young people to the intervention and the intervention to young people. We theorised that the components may work together to convert internalized understanding into externalised action. The framework is presented in Fig. 6 and definitions and exemplar descriptions are presented in Table 2.

**Table 2:** Definitions and exemplar descriptions of the equity relevant intervention components.

Component	Definition and exemplar descriptions
Coproduction components	
Content	***Does the intervention reflect what is relevant to young people?*** Intervention content is developed and refined by the young people the intervention is intended for. They are remunerated for their time and expertise.
Cultural stressors	***Does the intervention reflect relevant aetiology?*** Intervention design draws from theories, frameworks and lived experience to take account of the combination of societal and cultural stressors that affect underserved young people.
Context	***Does the intervention reflect young people's lives?*** Intervention design and content draw from young peoples lived experience to ensure it is relevant and applicable to them, how they relate to each other, and to the world around them.
Consilience	***Does the intervention reflect young people's access needs?*** Interventions are adaptable, offer multiple ways of engaging and are designed to avoid reliance on the quality, power and storage of the end-user's device.

Delivery dyads	
Representation	***Young people see themselves reflected in the intervention*** Intervention design and content delivery portrays young people of the same age, sexual orientation, ethnicity and culture as participants.
Customisation	***Young people can tailor the intervention to suit their individual needs and preferences*** Content is personalised according to young people's baseline characteristics and offers ways for young people to express their personality and creativity in how they engage with the intervention.
Structural provision and local linkage	***The intervention connects young people to other forms of support*** The intervention removes access barriers and uses features that help to reduce stigma or anxiety about accessing other services.
Guided practice	***The intervention guides young people through a planned programme*** The intervention structures content in a way that offers young people a sense of progress and achievement as they learn about the causes of health issues, and how they can address these.
Empowerment	***The intervention builds resilience, self-esteem, self-efficacy and assertiveness*** The intervention incorporates activities or games that enable young people to consider how they respond to different scenarios, practice skills, and receive feedback.
Belonging and connecting	***The intervention helps to grow the young person's support network*** The intervention links participants with others who they can share their experiences with, learn from, and practice skills with.

### 3.3 Intervention component results

The frequency of components by main outcome domain and focal equity relevant characteristics are presented in Table 3.

**Table 3:**
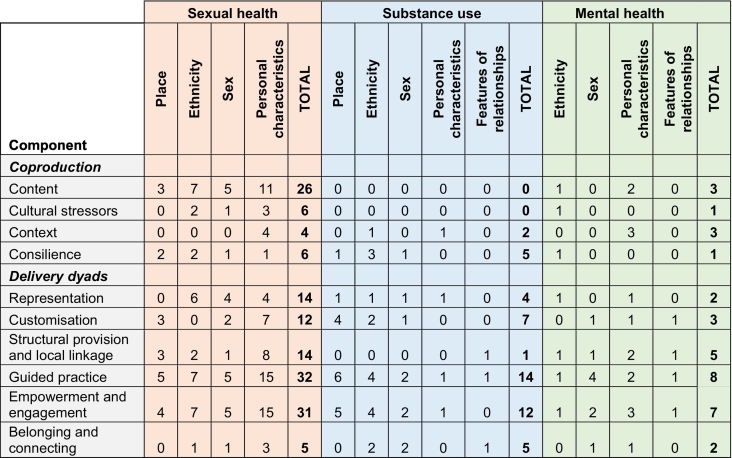
Frequency of components by main outcome domain and focal equity relevant characteristics.

#### 3.3.1 Coproduction components

##### 3.3.1.1 Content

Young people were involved in developing intervention content in sexual health interventions for young men who have sex with men (YMSM) (Bauermeister et al., 2024; Bauermeister et al., 2019; Biello et al., 2022; Biello et al., 2020; Biello et al., 2025; Hightow-Weidman et al., 2019; Mustanski et al., 2013; Mustanski et al., 2018; Reback et al., 2024; Schnall et al., 2022), girls (including ethnic minoritised girls) (Bennett et al., 2015; Chernick et al., 2022; Hebert et al., 2018; Jones et al., 2013; Manlove et al., 2020; Martínez-García et al., 2023; Stephenson et al., 2020), boys (Chernick et al., 2017), and transgender and gender expansive youth (Reiter et al., 2023). Coproduced content was also evident in mental health interventions for YMSM (Bauermeister et al., 2022; Egan et al., 2021) and Alaska Native and American Indian girls (Kaufman et al., 2025; Craig Rushing et al., 2021). Involvement was mainly facilitated through focus group discussions and user testing. In most cases, young people were remunerated for their involvement.

##### 3.3.1.2 Cultural stressors

Some interventions were informed by minority stress theory which describes the specific cumulative impact of social stressors and stigma on health outcomes of sexual minoritised individuals (Frost and Meyer, 2023). This was a feature of interventions for YMSM to reduce HIV risk (Schnall et al., 2022), improve HPV vaccination uptake (Reiter et al., 2023), reduce sexual risk behaviours (Hightow-Weidman et al., 2019), prevent drug use (Schwinn et al., 2015) and improve help seeking and coping strategies (Egan et al., 2021; Shen et al., 2023). Queer history was used to develop interventions to reduce stress and internalized stigma (Bauermeister et al., 2022), and improve HIV prevention (Bauermeister et al., 2019). Finally, a bicultural lens was used to help Hispanic youth balance navigating family values with those of non-Hispanic peers and adults in relation to substance use (Schwinn et al., 2021).

##### 3.3.1.3 Context

Sexual and mental health interventions often used coproduction to ensure interventions reflected young people's lives. This included drawing on insights from YMSM about same-sex attractions, dating behaviours, and popular YMSM-specific dating apps (Bauermeister et al., 2019), as well as designing modules and video game levels based on settings and situations that shape sexual decision making (Fiellin et al., 2017; Mustanski et al., 2018). Contraception interventions for girls were shaped by women's experiences of how access, barriers, and acceptability influenced their beliefs and choices (Stephenson et al., 2020). Sexual health and mental health interventions for Alaska Native and American Indian girls followed guidelines for culturally relevant technology-based health interventions (Kaufman et al., 2025; Craig Rushing et al., 2021), and integrated Indigenous learning heuristics such as the Medicine Wheel; a cultural symbol encompassing spiritual, emotional, mental, and physical wellness (Kaufman et al., 2025).

##### 3.3.1.4 Consilience

Some sexual health and substance use interventions addressed accessibility barriers. Smartphones were provided to African American girls and homeless youth (Jones et al., 2013; Santa Maria et al., 2021; Mason et al., 2016), internet-based content was made available on CD-ROM (Schinke et al., 2009; Schinke et al., 2005), and an online system at local youth clubs was used for those without phones to access messages (Bull et al., 2016). Interventions for drug prevention (Schwinn et al., 2021), contraception (Chernick et al., 2017; Martínez-García et al., 2023), and HIV prevention (Mustanski et al., 2025) for Hispanic youth were made available in English and Spanish.

#### 3.3.2 Delivery dyads

##### 3.3.2.1 Dyad 1 – representation and customisation

Facilitators, peers or actors reflecting participants' identities were used to deliver content through peer videos, soap operas, images and testimonials in sexual health interventions for YMSM (Bull et al., 2012; Mustanski et al., 2013; Mustanski et al., 2018; Schnall et al., 2022; Tan et al., 2022; Whiteley et al., 2018; Bauermeister et al., 2022), girls (Hebert et al., 2018; Patel et al., 2024; Stephenson et al., 2020) (including African American and Latina girls (Jones et al., 2013; Kissinger et al., 2023)), and boys (Carvalho et al., 2016), and substance use interventions for girls (Schwartz et al., 2014) and sexual and ethnic minoritised young people (Schinke et al., 2005; Schwinn et al., 2021; Schwinn et al., 2015). Content addressed misconceptions around HIV risks and STI testing, modelled communication and negotiation skills, provided medical information, and demonstrated conversations with healthcare professionals. Interventions for Alaska Native and American Indian girls used community role models to present content (Kaufman et al., 2025; Craig Rushing et al., 2021).

Sexual health interventions for YMSM, homeless young people, transgender and gender expansive youth, and urban youth used baseline demographic, sexual history and HIV/STI testing data to personalise content (Bauermeister et al., 2024; Biello et al., 2022; Biello et al., 2020; Biello et al., 2025; Cordova et al., 2020; Reiter et al., 2023; Santa Maria et al., 2021; Ybarra et al., 2021). Some interventions asked users to update this information regularly and used it tailor content further (Bauermeister et al., 2024; Biello et al., 2022; Biello et al., 2020; Biello et al., 2025; Reiter et al., 2023; Santa Maria et al., 2021). Contraception and HPV interventions for girls tailored content using baseline data, user preferences and pregnancy intentions (Bennett et al., 2015; Chernick et al., 2022; Stephenson et al., 2020). Substance use interventions provided home page and profile personalisation (Schwinn et al., 2010). Mental health interventions generated customised content and safety plans based on individual circumstances (Glass et al., 2024; Glass et al., 2022). Substance use interventions for minoritised ethnic boys and urban youth used individual and peer substance use behaviour information to offer personalised support (Gilmore et al., 2023; Mason et al., 2016; Mason et al., 2015; Prokhorov et al., 2008). Mental health and sexual health interventions for YMSM and substance use interventions for urban youth allowed participants to customise their on-screen character (Christensen et al., 2013; Walton et al., 2013; Walton et al., 2014; Egan et al., 2021).

##### 3.3.2.2 Dyad 2 - structural provision and local linkage and guided practice

Sexual health interventions for YMSM used clinic locators (Gerend et al., 2020; Hightow-Weidman et al., 2019), GPS-enabled maps, and geolocation notifications for HIV/STI testing and PrEP provision (Biello et al., 2022; Biello et al., 2020; Biello et al., 2025). Some ranked testing sites using “mystery shopper” data (Bauermeister et al., 2024), distributed free home-order HIV/STI kits, condoms, and lubricant (Biello et al., 2022; Biello et al., 2020; Biello et al., 2025), and supported HIV self-testing via mobile image processing (Schnall et al., 2024). Sexual health interventions for Black and Latina girls also provided reproductive health clinic locators (Manlove et al., 2020). Interventions also provided contact with professionals and counsellors for sexual health advice, guidance, and referrals for YMSM (Biello et al., 2022; Biello et al., 2020; Biello et al., 2025; Hightow-Weidman et al., 2019; Mustanski et al., 2025), and urban youth (Chernick et al., 2025; Chernick et al., 2022; Cordova et al., 2020). Resources linked LGBTQ+ youth to sexual health and mental health content (Bauermeister et al., 2022; Egan et al., 2021), and offered those facing IPV advocates and personalised resources (Glass et al., 2024; Glass et al., 2022). Interventions for Alaska Native and American Indian youth focussed on destigmatising mental health services and encouraged engagement with tribal clinics and chat lines (Craig Rushing et al., 2021). Alcohol reduction interventions for youth with alcohol dependent parents guided them to social services and health centres (Wall et al., 2024; Walton et al., 2013).

Most interventions across all outcome domains and groups used structured modules or messages to build young people's understanding of health and decision making. Content was usually delivered through regular, structured modules or messages. Some incorporated responsive programming which reinforced positive choices or prompted users to rethink their answers.

##### 3.3.2.3 Dyad 3 - empowerment and engagement and belonging and connecting

Sexual health interventions for YMSM used role-play and video games to practice negotiation, reduce risk behaviours, and combat isolation (Bauermeister et al., 2019; Bull et al., 2012; Christensen et al., 2013; Fiellin et al., 2017). They also encouraged HIV/STI prevention and testing goal setting and provided forums for sharing personal stories (Biello et al., 2022; Biello et al., 2020; Biello et al., 2025; Hightow-Weidman et al., 2019; Mustanski et al., 2013; Mustanski et al., 2018; Santa Maria et al., 2021; Schnall et al., 2024). For YMSM and girls, sexual health interventions offered demonstrations and activities to improve confidence to communicate with healthcare professionals (Bennett et al., 2015; Hebert et al., 2018; Jones et al., 2013; Patel et al., 2024), service rating (Bauermeister et al., 2024), and scripted responses to relationship questions (Ybarra et al., 2021; Ybarra et al., 2017). Mental health interventions for YMSM provided forums to share personal stories (Bauermeister et al., 2022), identity affirming video game challenges (Egan et al., 2021) and stress management tools (Shen et al., 2023). Girls were provided with activities to promote body positivity (Aboody et al., 2020), create safety plans (Glass et al., 2024; Glass et al., 2022), and mentor younger girls (Starosta et al., 2016). Substance use interventions used video game activities, interactive games and role playing to encourage urban youths, YMSM and Hispanic youth to practice problem solving skills (Schinke et al., 2005; Schwinn et al., 2021; Schwinn et al., 2015; Walton et al., 2013; Walton et al., 2014). Girls were provided with quizzes on navigating self-esteem, assertion and peer pressure (Schwinn et al., 2010), and negotiation exercises for mothers and daughters (Fang et al., 2010; Schinke et al., 2009).

Mental health interventions for YMSM and girls facilitated peer discussion and support through chat interfaces (Bauermeister et al., 2022) and text messages (Jeong et al., 2024). Sexual health interventions for YMSM, girls, and transgender and gender expansive youth provided peer discussion forums (Bull et al., 2012; Hightow-Weidman et al., 2019; Reback et al., 2024), and paired participants together for skill practice and support (Ybarra et al., 2021; Ybarra et al., 2017). Substance use interventions for girls and Hispanic youth involved their parent(s) to strengthen family support (Fang et al., 2010; Schinke et al., 2009; Schwinn et al., 2021), or provided discussion forums for them to chat and share content with other participants (Schwinn et al., 2010). Youth with alcohol dependent parents were encouraged to talk to friends or teachers (Wall et al., 2024).

## 4 Discussion

This review explored how digital PH interventions for sexual health, substance use and mental health are tailored to underserved young people, and evidence of effectiveness.

Most evidence of tailored interventions was for sexual health, predominantly for YMSM. Evidence of tailored substance use and mental health interventions was limited. There were also notable differences in gender-based interventions, with most sexual health and mental health interventions for heterosexual young people tailored to girls (Bennett et al., 2015; Martínez-García et al., 2023; Patel et al., 2024; Starosta et al., 2016; Stephenson et al., 2020; Aboody et al., 2020; Ampatzoglou et al., 2024; Glass et al., 2022).

### 4.1 Effectiveness

The most promising and consistent evidence for tailored interventions was on reducing cannabis and other drug use. Whilst an overall positive gradient of effects was also observed for alcohol, smoking, condom use, STI/HIV testing, STI/HIV diagnoses, PrEP uptake and anxiety and depression, effects were less clear and did not always reach statistical significance. Whilst positive gradients of effects were indicated for HPV vaccination uptake, suicide risk, pregnancy and contraception use this was based on a small number of trials. Finally, there was lack of evidence of any gradient of effects on mental wellbeing, sexual risk behaviours, LARC uptake or improving access to healthcare.

### 4.2 Design features

We developed a framework which comprised four coproduction components and three dyads of delivery components. Together these components may work to engage young people and turn knowledge into action. Despite well-established guidance on the importance of involving young people in development of interventions (World Health Organisation, 2021), evidence from this review highlights coproduction practices within intervention design were not consistently used outside of sexual health interventions and with sexual and ethnic minoritised young people (Bauermeister et al., 2024; Bauermeister et al., 2019; Biello et al., 2022; Biello et al., 2020; Biello et al., 2025; Gerend et al., 2020; Hightow-Weidman et al., 2019; Mustanski et al., 2013; Mustanski et al., 2025; Mustanski et al., 2018; Schnall et al., 2022). Although not consistently used, we found similar delivery features were used across all outcome domains and underserved groups. This indicates potential synergies in the ways that interventions may engage young people and build knowledge and skills that can help them navigate these interlinked health issues.

### 4.3 Strengths and limitations

Our comprehensive search strategy and inclusive definition of digital public health interventions enabled us to capture a wide range of relevant evidence. By examining features of mental health, substance use and sexual health interventions tailored for diverse groups, we were able to identify shared approaches that may better reflect how these connected issues affect underserved young people, and how digital interventions might address them.

However, it is likely that some eligible trials were missed. ICA requires subjective engagement to define key intervention features. It is therefore possible that a different group of researchers may generate different coding and findings. Additionally, our review may have been limited by simplified or incomplete reporting of intervention descriptions which is a common problem in complex, social interventions. We were unable to assess effectiveness using meta-analyses. Whilst using harvest plots allowed us to synthesise heterogenous data and include a range of dimensions, the focus on significance tests limits precision of conclusions on effectiveness. Additionally, most trials were assessed as high risk of bias which also may reduce the certainty of evidence presented. Finally, we were unable to analyse which components or combination of components may be related to effectiveness. Therefore, our framework offers an explanatory account of how intervention components may work and potential synergies across outcome domains rather than causal claims.

### 4.4 Implications for research, policy and practice

Our framework may help trialists, developers and practitioners consider what options are available to tailor digital PH interventions to underserved young people and offer a basis for future testing. It contributes to existing literature by providing further details regarding how coproduction can be used for digital intervention development to improve relevance and acceptability (Brotherdale et al., 2024). This review also highlights the need to evaluate the effectiveness of tailored interventions beyond those for sexual health and with a broader range of underserved groups such as youth with learning disabilities, those excluded from school, and care experienced young people (Evans et al., 2025). This is especially important to ensure the rapid progress of digital interventions in mental health strategies are informed by evidence of how they can work for underserved groups (Department for Health and Social Care, 2025; NICE, 2023; NHS Confederation, 2023). Developing and evaluating interventions for a wider range of underserved groups requires a deeper understanding of what different individuals, groups and communities consider important. Approaches such as cultural adaptation frameworks, community engagement, engagement with external experts (Leung et al., 2025) and use of technology (Demetry et al., 2026) may support trialists and developers to identify what is relevant and acceptable for diverse groups, improving the quality of future intervention evaluations. Finally, addressing accessibility barriers may be costly when delivering interventions at scale. Therefore, definitive and pragmatic trials should incorporate implementation and cost-effectiveness evidence to ensure practitioners can substantiate investment decisions.

## 5 Conclusion

Tailored interventions produced their clearest effects in reducing cannabis and other drug use, with smaller and often non-significant effects across depression, anxiety, smoking, alcohol use and most sexual health behavioural and biological outcomes. No meaningful effects were indicated for mental wellbeing, sexual risk behaviours, LARC uptake or healthcare access. Our framework supports an understanding of how tailored interventions may work to reach and engage underserved young people that may inform future intervention design and evaluation.

## CRediT authorship contribution statement

*Claire Tatton* – conceptualisation, data curation, formal analysis, investigation, visualisation, writing – original draft, writing – review and editing.

*Joelle Kirby* - conceptualisation, data curation, methodology, investigation, writing – review and editing.

*Jessica Armitage* - conceptualisation, data curation, formal analysis, investigation, visualisation, writing – review and editing.

*Sophie Robinson* - conceptualisation, data curation, methodology, investigation, writing – review and editing.

*Rabeea'h Waseem Aslam* - conceptualisation, data curation, investigation, writing – review and editing.

*Tom Arthur* - conceptualisation, data curation, investigation, writing – review and editing.

*Sean Harrison* - conceptualisation, writing – review and editing.

*Daniel Mutanda* - conceptualisation, writing - review and editing.

*Ruth Garside* - conceptualisation, funding acquisition, writing – review and editing.

*Jo Thompson Coon* - conceptualisation, funding acquisition, writing – review and editing.

*Rhiannon Evans* - conceptualisation, funding acquisition, writing – review and editing.

*G.J. Melendez-Torres* - conceptualisation, data curation, methodology, formal analysis, funding acquisition, investigation, project administration, writing – review and editing, visualisation, supervision.

## Funding

National Institute for Health and Care Research 10.13039/501100001921Public Health Research Programme (NIHR175227) This research was supported by the National Institute for Health and Care Research Applied Research Collaboration South West Peninsula. Prof G.J. Melendez-Torres is an NIHR Senior Investigator. Prof JS Chandan is an 10.13039/100006662NIHR Research Professor (NIHR306365). The views expressed in this publication are those of the author(s) and not necessarily those of the National Institute for Health and Care Research or the Department of Health and Social Care.

## Declaration of competing interest

None to report.
