# Supplementary material for: Inclusion of underserved young people in digital public health interventions for mental health, sexual health and substance use: A systematic review and intervention component analysis
Source: Internet Interv. 2026 Aug 6;45:100985. doi: 10.1016/j.invent.2026.100985 (PMC13475656; doi:10.1016/j.invent.2026.100985)
Supplement: Supplementary file 1 [file mmc1.pdf]

Inclusion of underserved young people in digital public health interventions for mental health, sexual health and substance use. An intervention component analysis.

Supplementary file

Table of Contents

Eligibility criteria **1**

Search strings – phase 1 search for systematic reviews **2**

a) Summary 3

b) Ovid MEDLINE(R) ALL Search 3

c) Embase 7

d) APA PsycInfo 11

e) Web of Science 15

f) Scopus 16

Search strings – phase 2 search for randomised trials **16**

a) Summary 16

b) Database: CENTRAL 17

c) Database: APA PsycInfo 19

Main trial reports included **21**

Reports of long-term outcomes to main trials included **27**

Trial and participant characteristics **28**

a) Sexual health interventions 29

b) Substance use interventions 37

c) Mental health interventions 40

Intervention details **42**

a) Sexual health interventions 42

b) Substance use interventions 58

c) Mental health interventions 64

Outcomes **67**

a) Sexual health interventions 67

b) Substance use interventions 78

c) Mental health interventions 82

Risk of bias assessments **84**

Eligibility criteria

	Inclusion	Exclusion
**Population**	Young people aged 13-25.	Adults above the age of 25 years old.<br><br>Children under the age of 13 years old.<br><br>For mental health: populations that are diagnostic in nature, beyond mild/moderate mental health problems (e.g. with clinical diagnoses of mood disorders, or of other chronic or acute conditions).<br><br>For substance use: populations that have a clinical diagnosis of addiction, such as alcohol use disorder (except for nicotine/tobacco).
**Interventions**	These interventions are interventions that solely or primarily use digital technologies for the delivery of intervention content.<br><br>Included interventions might include,<br>• mobile apps.<br>• social media-based interventions.<br>• other text message-based interventions.	Interventions using digital technologies,<br><br>• for measurement or assessment (e.g. wearables, pedometers) without any linked digital delivery of content).<br>• of one-time passive delivery of information (e.g. a single video or static webpage).<br>• delivered to a pre-existing group (e.g. to entire classes in schools).<br>• designed as telehealth/telemedicine, i.e. for online provision of clinical services, or for individual management of health services.<br>• Requiring significant therapist or face to face contact
**Comparator**	Any comparator, including waitlist, no-treatment control, another digital intervention, or a face-to-face intervention.	n/a.
**Country**	High-income.	Low-income and middle-income countries.
**Setting**	Online.<br>Community.<br>Peer support.	Hospital-based (excepting initiation)
**Outcomes**	Mental health, including symptomatology of depression or anxiety, internalising and externalising problems, and mental well-being. We are specifically interested in outcomes related to suicide, depression anxiety, mental well-being, suicide and self-harm, and first episode psychosis.<br><br>Sexual health, including sexually transmitted infections, sexual risk behaviours, uptake of contraception or protective technologies (barrier methods, vaccination, pre-exposure prophylaxis).<br><br>Substance use, including use of alcohol, tobacco, or other drugs.	Severe mental illness, a diagnosed mental health condition.<br><br>Substance use based on addictive behaviours, for example gambling and internet addiction.<br><br>Sexual violence outcomes.
**Study types**	Phase 1 of searching: Systematic reviews where reviews focus specifically on randomised controlled trials, scoping reviews, literature reviews.<br><br>Phase 2 of searching: Randomised controlled trials	Phase 1 of searching: Scoping reviews not focused on evaluations of relevant interventions, or focusing on digital health more generally, will be excluded.<br><br>Phase 2 of searching: Non-randomised controlled studies

Search strings – phase 1 search for systematic reviews

a) Summary

Source	Date	Number of hits
**Medline (Ovid)**	20/01/25	5,023
**Embase (Ovid)**	20/01/25	6,451
**PsycInfo (Ovid)**	21/01/25	1,986
**Web of Science**	21/01/25	347
**Scopus**	21/01/25	221
**Total**		14,028
**After deduplication**		8,714

b) Ovid MEDLINE(R) ALL Search

Date of search: 20th of January 2025

Search period: 1946 to 17th of January 2025

#	Query	Results from 20 Jan 2025
1	exp Mental Disorders/pc [Prevention & Control]	57,243
2	Mental Health/	70,922
3	Mindfulness/	7,613
4	Stress, Psychological/	139,904
5	Depression/	165,576
6	Anxiety/	118,208
7	Resilience, Psychological/	10,775
8	(mental* or psych* or ?mood*).ti.	580,211
9	(mental* adj3 health*).ab,kf.	254,675
10	(depress* or anxious* or anxiet* or emotion* or mood or resilience or self-efficacy or self-esteem or mindful* or cope or coping or cognitive* or lifeskills or "life skills" or stress* or psychosocial or "psychological* distress*").ti,kf.	1,102,357
11	(well-being or well being or wellbeing).ti,ab,kf.	173,042
12	((social or emotional) adj1 (skills or support or network*)).ti,ab,kf.	103,509
13	((depress* or anxiet*) adj5 (symptom* or prevent*)).ab.	147,341
14	1 or 2 or 3 or 4 or 5 or 6 or 7 or 8 or 9 or 10 or 11 or 12 or 13	1,983,979
15	exp Substance-Related Disorders/pc [Prevention & Control]	26,257
16	(cannabis or marijuana or "substance use").ti.	40,043
17	"legal high*".tw.	446
18	exp "Marijuana Use"/	7,544
19	Cannabis/	15,467
20	"Tobacco Use Disorder"/pc [Prevention & Control]	1,954
21	"Tobacco Use Cessation"/	1,556
22	Smoking Reduction/	123
23	Smoking Cessation/	34,131
24	exp Alcohol Drinking/pc [Prevention & Control]	5,477
25	binge drinking/ or alcohol drinking in college/ or underage drinking/	4,841
26	(alcohol* or beer* or wine* or liqo?r* or spirits or alcopop*).ti.	186,714
27	15 or 16 or 17 or 18 or 19 or 20 or 21 or 22 or 23 or 24 or 25 or 26	286,105
28	exp Substance-Related Disorders/ or Illicit Drugs/	329,616
29	exp *Alcoholic Beverages/	17,980
30	exp *Smoking/	84,060
31	(Drug* or substance* or polysubstance or polydrug or "poly-drug" or "legal high*" or cannabis or weed or marijuana or skunk or cocaine or stimulant* or ketamine or pingers	2,594,339

	or MDMA or Molly or ecstasy or hotboxing or "hot boxing" or hallucinogen* or inhalant* or psychoactive* or "psycho active*").ti,ab,kf.	
32	(smoking or smoke* or tobacco* or nicotine or cigar? or cigs or cigarette* or cigarilloi* or "hand roll*" or handroll* or rollies or "roll up*" or "roll-up*" or rollup* or vape or vaping or ecig* or e-cig* or "e cig*").ti,ab,kf.	454,300
33	(drink* or alcohol* or beer* or wine* or liqo?r* or spirits or alcopop*).ti,ab,kf.	580,626
34	28 or 29 or 30 or 31 or 32 or 33	3,495,501
35	*Health Promotion/	54,246
36	Psychosocial Intervention/	1,370
37	exp Preventive Health Services/	711,011
38	Primary Prevention/	20,392
39	exp Public Health/	9,792,229
40	("public health" or "health promotion" or prevent* or avoid* or reduc* or protect* or abstain* or abstinence or refus* or stop* or delay or influenc* or reduc* or abstain* or abstinence or temperance or refus* or prevent* or stop* or delay* or binge* or excessive* or underage* or under-age* or "under age*" or high-risk or "high risk" or behavio?r* or teen* or youth* or adolescen* or juvenile* or youngster* or highschool* or college* or universit* or school* or education).ti,ab,kf.	11,758,031
41	35 or 36 or 37 or 38 or 39 or 40	17,625,197
42	34 and 41	2,083,117
43	27 or 42	2,176,362
44	exp Sexual Behavior/	130,637
45	Unsafe Sex/	5,290
46	exp Sexually Transmitted Diseases/pc [Prevention & Control]	77,166
47	(sexually transmitted disease* or std* or STI*).ti.	431,616
48	(sex* adj2 (health* or safe* or unsafe or protect* or risk or risky or high-risk or unprotected or abstinence or behavio?r* or activit*)).ti,ab,kf.	97,494
49	((STI or STIs or STD or STDs or sexually transmitted disease* or sexually transmitted infection* or Chlamydia or gonorrhoea or syphilis) and (prevent* or control* or risk* or reduc*)).ti,kf.	8,515
50	(contraception or contraceptive* or condom* or "HPV vaccine" or "human papillomavirus" or "pre-exposure prophylaxis").ti,ab,kf.	156,450
51	Safe Sex/	3,627
52	Pregnancy in Adolescence/pc [Prevention & Control]	1,594
53	Sexual Health/	3,184
54	(sexual adj3 (health* or behavio?r*)).ti,ab,kf.	58,782
55	Contraception/	22,939
56	Condoms/	11,353
57	*Reproductive Health Services/	2,043
58	44 or 45 or 46 or 47 or 48 or 49 or 50 or 51 or 52 or 53 or 54 or 55 or 56 or 57	791,734

59	14 or 43 or 58	4,607,970
60	Digital Technology/	1,308
61	Mobile Applications/	14,050
62	Wearable Electronic Devices/	10,469
63	exp Cell Phone/	25,075
64	Videoconferencing/	2,515
65	gamification/	141
66	(digital* or online* or internet* or web* or email or mobile or app or apps or browser or virtual).ti.	251,755
67	((Digital* or online or web* or internet or virtual* or email*) adj2 (technolog* or intervention* or deliver* or based or support*)).ab,kw.	100,551
68	(smartphone* or (smart adj phone*)).tw.	29,540
69	(mobile adj (device* or health or phone*)).tw.	25,031
70	text messag*.tw.	7,128
71	(e-health or ehealth or m-health or mhealth).tw.	15,140
72	(SMS or MMS).tw.	15,224
73	wearable*.tw.	32,615
74	(facebook or twitter or snapchat or facetime or Tiktok or youtube or whatsapp or Instagram or reddit or blog or blogs or blogging or webinar* or podcast*).tw.	26,844
75	(social adj (media or network*)).tw.	60,593
76	(smart adj (device* or tech*)).tw.	2,252
77	(electronic adj2 device*).tw.	18,391
78	((mobile or online or tablet or computer or phone) adj2 (app or apps)).ab.	7,391
79	(Zoom or skype or "video conferenc*").tw.	6,919
80	virtual reality/	7,581
81	(virtual adj2 (realit* or world* or environment*)).tw.	25,299
82	3d vision.tw.	346
83	Gamification.tw.	1,591
84	60 or 61 or 62 or 63 or 64 or 65 or 66 or 67 or 68 or 69 or 70 or 71 or 72 or 73 or 74 or 75 or 76 or 77 or 78 or 79 or 80 or 81 or 82 or 83	497,602
85	59 and 84	118,204
86	(systematic review or meta-analysis).pt.	372,386
87	meta-analysis/ or systematic review/ or systematic reviews as topic/ or meta-analysis as topic/ or "meta analysis (topic)"/ or "systematic review (topic)"/ or exp technology assessment, biomedical/ or network meta-analysis/	416,035
88	((systematic* adj3 (review* or overview*)) or (methodologic* adj3 (review* or overview*))).ti,ab,kf.	398,156
89	((quantitative adj3 (review* or overview* or synthes*)) or (research adj3 (integrati* or overview*))).ti,ab,kf.	18,955

90	((integrative adj3 (review* or overview*)) or (collaborative adj3 (review* or overview*)) or (pool* adj3 analy*)).ti,ab,kf.	45,198
91	(data synthes* or data extraction* or data abstraction*).ti,ab,kf.	49,251
92	(handsearch* or hand search*).ti,ab,kf.	11,897
93	(mantel haenszel or peto or der simonian or dersimonian or fixed effect* or latin square*).ti,ab,kf.	40,430
94	(met analy* or metanaly* or technology assessment* or HTA or HTAs or technology overview* or technology appraisal*).ti,ab,kf.	13,755
95	(meta regression* or metaregression*).ti,ab,kf.	17,773
96	(meta-analy* or metaanaly* or systematic review* or biomedical technology assessment* or bio-medical technology assessment*).mp,hw.	557,149
97	(medline or cochrane or pubmed or medlars or embase or cinahl).ti,ab,hw.	413,180
98	(cochrane or (health adj2 technology assessment) or evidence report).jw.	22,357
99	(comparative adj3 (efficacy or effectiveness)).ti,ab,kf.	20,360
100	(outcomes research or relative effectiveness).ti,ab,kf.	12,144
101	((indirect or indirect treatment or mixed-treatment or bayesian) adj3 comparison*).ti,ab,kf.	4,874
102	(multi* adj3 treatment adj3 comparison*).ti,ab,kf.	321
103	(mixed adj3 treatment adj3 (meta-analy* or metaanaly*)).ti,ab,kf.	184
104	umbrella review*.ti,ab,kf.	2,488
105	(multi* adj2 paramet* adj2 evidence adj2 synthesis).ti,ab,kf.	15
106	(multiparamet* adj2 evidence adj2 synthesis).ti,ab,kf.	19
107	(multi-paramet* adj2 evidence adj2 synthesis).ti,ab,kf.	13
108	86 or 87 or 88 or 89 or 90 or 91 or 92 or 93 or 94 or 95 or 96 or 97 or 98 or 99 or 100 or 101 or 102 or 103 or 104 or 105 or 106 or 107	807,592
109	85 and 108	7,602
110	limit 109 to yr="2020 -Current"	5,023

c) Embase

Date of search: 20th of January, 2025

Search period: 1974 to 17th of January, 2025

#	Query	Results from 20 Jan 2025
1	exp *mental disease/pc [Prevention]	42,264
2	mental health/	244,305
3	mindfulness/	17,607
4	mental stress/	104,420
5	depression/	538,449
6	anxiety/	321,725
7	psychological resilience/	12,515
8	(mental* or psych* or ?mood*).ti.	639,984

9	(mental* adj3 health*).ab,kf.	310,995
10	(depress* or anxious* or anxiet* or emotion* or mood or resilience or self-efficacy or self-esteem or mindful* or cope or coping or cognitive* or lifeskills or "life skills" or stress* or psychosocial or "psychological* distress*").ti,kf.	1,376,737
11	(well-being or well being or wellbeing).ti,ab,kf.	208,337
12	((social or emotional) adj1 (skills or support or network*)).ti,ab,kf.	124,278
13	((depress* or anxiet*) adj5 (symptom* or prevent*)).ab.	194,347
14	1 or 2 or 3 or 4 or 5 or 6 or 7 or 8 or 9 or 10 or 11 or 12 or 13	2,602,884
15	exp drug dependence/pc [Prevention]	8,407
16	(cannabis or marijuana or "substance use").ti.	48,671
17	"legal high*".tw.	655
18	exp "Marijuana Use"/	21,059
19	Cannabis/	48,518
20	exp smoking/pc [Prevention]	7,346
21	smoking cessation/	73,016
22	smoking reduction/	530
23	drinking behavior/pc [Prevention]	1,192
24	binge drinking/	8,423
25	college drinking/	846
26	underage drinking/	1,938
27	(alcohol* or beer* or wine* or liqo?r* or spirits or alcopop*).ti.	228,016
28	15 or 16 or 17 or 18 or 19 or 20 or 21 or 22 or 23 or 24 or 25 or 26 or 27	388,147
29	exp *"substance use"/	154,183
30	exp *alcoholic beverage/	14,398
31	exp *smoking/	101,779
32	(Drug* or substance* or polysubstance or polydrug or "poly-drug" or "legal high*" or cannabis or weed or marijuana or skunk or cocaine or stimulant* or ketamine or pingers or MDMA or Molly or ecstasy or hotboxing or "hot boxing" or hallucinogen* or inhalant* or psychoactive* or "psycho active*").ti,ab,kf.	3,451,014
33	(smoking or smoke* or tobacco* or nicotine or cigar? or cigs or cigarette* or cigarilloi* or "hand roll*" or handroll* or rollies or "roll up*" or "roll-up*" or rollup* or vape or vaping or ecig* or e-cig* or "e cig*").ti,ab,kf.	647,322
34	(drink* or alcohol* or beer* or wine* or liqo?r* or spirits or alcopop*).ti,ab,kf.	771,629
35	29 or 30 or 31 or 32 or 33 or 34	4,553,542
36	exp *health promotion/	43,264
37	psychosocial intervention/	3,179
38	exp preventive health service/	31,850
39	primary prevention/	49,160
40	exp public health/	248,665

41	("public health" or "health promotion" or prevent* or avoid* or reduc* or protect* or abstain* or abstinence or refus* or stop* or delay or influenc* or reduc* or abstain* or abstinence or temperance or refus* or prevent* or stop* or delay* or binge* or excessive* or underage* or under-age* or "under age*" or high-risk or "high risk" or behavio?r* or teen* or youth* or adolescen* or juvenile* or youngster* or highschool* or college* or universit* or school* or education).ti,ab,kf.	14,778,140
42	36 or 37 or 38 or 39 or 40 or 41	14,891,518
43	35 and 42	2,187,990
44	28 or 43	2,362,213
45	exp sexual behavior/	264,602
46	exp unsafe sex/	8,370
47	sexually transmitted disease/pc [Prevention]	8,637
48	(sexually transmitted disease* or std* or STI*).ti.	491,171
49	(sex* adj2 (health* or safe* or unsafe or protect* or risk or risky or high-risk or unprotected or abstinence or behavio?r* or activit*)).ti,ab,kf.	128,430
50	((STI or STIs or STD or STDs or sexually transmitted disease* or sexually transmitted infection* or Chlamydia or gonorrhoea or syphilis) and (prevent* or control* or risk* or reduc*)).ti,kf.	9,369
51	(contraception or contraceptive* or condom* or "HPV vaccine" or "human papillomavirus" or "pre-exposure prophylaxis").ti,ab,kf.	184,262
52	exp safe sex/	12,733
53	adolescent pregnancy/pc [Prevention]	465
54	exp sexual health/	25,015
55	(sexual adj3 (health* or behavio?r*)).ti,ab,kf.	76,398
56	exp contraception/	188,006
57	exp contraceptive device/	50,503
58	45 or 46 or 47 or 48 or 49 or 50 or 51 or 52 or 53 or 54 or 55 or 56 or 57	1,094,662
59	14 or 44 or 58	5,613,374
60	exp Digital Technology/	7,094
61	Mobile Application/	25,621
62	Wearable Electronic Devices/	2,907
63	exp Cell Phone/	55,441
64	videoconferencing/	10,645
65	gamification/	823
66	(digital* or online* or internet* or web* or email or mobile or app or apps or browser or virtual).ti.	286,986
67	((Digital* or online or web* or internet or virtual* or email*) adj2 (technolog* or intervention* or deliver* or based or support*)).ab,kw.	128,473
68	(smartphone* or (smart adj phone*)).tw.	37,169
69	(mobile adj (device* or health or phone*)).tw.	29,049

70	text messag*.tw.	9,132
71	(e-health or ehealth or m-health or mhealth).tw.	16,889
72	(SMS or MMS).tw.	20,757
73	wearable*.tw.	34,981
74	(facebook or twitter or snapchat or facetime or Tiktok or youtube or whatsapp or Instagram or reddit or blog or blogs or blogging or webinar* or podcast*).tw.	35,402
75	(social adj (media or network*)).tw.	72,205
76	(smart adj (device* or tech*)).tw.	2,504
77	(electronic adj2 device*).tw.	19,106
78	((mobile or online or tablet or computer or phone) adj2 (app or apps)).ab.	8,930
79	(Zoom or skype or "video conferenc*").tw.	10,976
80	virtual reality/	31,432
81	(virtual adj2 (realit* or world* or environment*)).tw.	30,222
82	3d vision.tw.	566
83	Gamification.tw.	1,654
84	60 or 61 or 62 or 63 or 64 or 65 or 66 or 67 or 68 or 69 or 70 or 71 or 72 or 73 or 74 or 75 or 76 or 77 or 78 or 79 or 80 or 81 or 82 or 83	613,788
85	59 and 84	148,224
86	(systematic review or meta-analysis).pt.	0
87	meta-analysis/ or systematic review/ or systematic reviews as topic/ or meta-analysis as topic/ or "meta analysis (topic)"/ or "systematic review (topic)"/ or exp technology assessment, biomedical/ or network meta-analysis/	726,825
88	((systematic* adj3 (review* or overview*)) or (methodologic* adj3 (review* or overview*))).ti,ab,kf.	473,356
89	((quantitative adj3 (review* or overview* or synthes*)) or (research adj3 (integrati* or overview*))).ti,ab,kf.	21,418
90	((integrative adj3 (review* or overview*)) or (collaborative adj3 (review* or overview*)) or (pool* adj3 analy*)).ti,ab,kf.	62,660
91	(data synthes* or data extraction* or data abstraction*).ti,ab,kf.	58,742
92	(handsearch* or hand search*).ti,ab,kf.	14,399
93	(mantel haenszel or peto or der simonian or dersimonian or fixed effect* or latin square*).ti,ab,kf.	52,800
94	(met analy* or metanaly* or technology assessment* or HTA or HTAs or technology overview* or technology appraisal*).ti,ab,kf.	23,314
95	(meta regression* or metaregression*).ti,ab,kf.	21,341
96	(meta-analy* or metaanaly* or systematic review* or biomedical technology assessment* or bio-medical technology assessment*).mp,hw.	856,300
97	(medline or cochrane or pubmed or medlars or embase or cinahl).ti,ab,hw.	526,701
98	(cochrane or (health adj2 technology assessment) or evidence report).jw.	32,096
99	(comparative adj3 (efficacy or effectiveness)).ti,ab,kf.	29,393

100	(outcomes research or relative effectiveness).ti,ab,kf.	17,301
101	((indirect or indirect treatment or mixed-treatment or bayesian) adj3 comparison*).ti,ab,kf.	8,529
102	[(meta-analysis or systematic review).md.]	0
103	(multi* adj3 treatment adj3 comparison*).ti,ab,kf.	452
104	(mixed adj3 treatment adj3 (meta-analy* or metaanaly*)).ti,ab,kf.	263
105	umbrella review*.ti,ab,kf.	2,556
106	(multi* adj2 paramet* adj2 evidence adj2 synthesis).ti,ab,kf.	36
107	(multiparamet* adj2 evidence adj2 synthesis).ti,ab,kf.	22
108	(multi-paramet* adj2 evidence adj2 synthesis).ti,ab,kf.	31
109	or/86-108	1,139,783
110	85 and 109	10,234
111	limit 110 to yr="2020 -Current"	6,451

d) APA PsycInfo

Date of search: 21st of January, 2025
Search period: 1806 to Week 2 of January, 2025

#	Query	Results from 21 Jan 2025
1	mental disorders/	102,488
2	exp mental health/	106,660
3	mindfulness/	14,069
4	"resilience (psychological)"/	25,766
5	"depression (emotion)"/	27,322
6	exp anxiety/	100,336
7	exp stress/	160,855
8	public mental health/	1,040
9	exp *well being/	55,206
10	(mental* or psych* or ?mood*).ti.	656,437
11	(mental* adj3 health*).ab.	253,189
12	(depress* or anxious* or anxiet* or emotion* or mood or resilience or self-efficacy or self-esteem or mindful* or cope or coping or cognitive* or lifeskills or "life skills" or stress* or psychosocial or "psychological* distress*").ti.	621,751
13	(well-being or well being or wellbeing).ti,ab.	141,040
14	((social or emotional) adj1 (skills or support or network*)).ti,ab.	115,049
15	((depress* or anxiet*) adj5 (symptom* or prevent*)).ab.	122,156
16	1 or 2 or 3 or 4 or 5 or 6 or 7 or 8 or 9 or 10 or 11 or 12 or 13 or 14 or 15	1,553,730
17	binge drinking/ or social drinking/ or underage drinking/	5,559
18	(cannabis or marijuana or "substance use").ti.	28,907

19	marijuana/	3,953
20	"cannabis use"/	4,587
21	"legal high*".tw.	197
22	exp drug abstinence/	20,315
23	exp "substance use prevention"/	5,411
24	(alcohol* or beer* or wine* or liqo?r* or spirits or alcopop*).ti.	72,182
25	17 or 18 or 19 or 20 or 21 or 22 or 23 or 24	124,626
26	exp alcoholic beverages/	3,643
27	"alcohol use attitudes"/	3,378
28	exp *tobacco smoking/	33,450
29	(Drug* or substance* or polysubstance or polydrug or "poly-drug" or "legal high*" or cannabis or weed or marijuana or skunk or cocaine or stimulant* or ketamine or pingers or MDMA or Molly or ecstasy or hotboxing or "hot boxing" or hallucinogen* or inhalant* or psychoactive* or "psycho active*").ti,ab.	345,205
30	(smoking or smoke* or tobacco* or nicotine or cigar? or cigs or cigarette* or cigarilloi* or "hand roll*" or handroll* or rollies or "roll up*" or "roll-up*" or rollup* or vape or vaping or ecig* or e-cig* or "e cig*").ti,ab.	86,072
31	(drink* or alcohol* or beer* or wine* or liqo?r* or spirits or alcopop*).ti,ab.	173,333
32	26 or 27 or 28 or 29 or 30 or 31	505,138
33	exp *health promotion/	25,548
34	psychosocial interventions/	2,141
35	exp preventive health services/	7,805
36	exp *public health/	131,566
37	("public health" or "health promotion" or prevent* or avoid* or reduc* or protect* or abstain* or abstinence or refus* or stop* or delay or influenc* or reduc* or abstain* or abstinence or temperance or refus* or prevent* or stop* or delay* or binge* or excessive* or underage* or under-age* or "under age*" or high-risk or "high risk" or behavio?r* or teen* or youth* or adolescen* or juvenile* or youngster* or highschool* or college* or universit* or school* or education).ti,ab.	3,080,570
38	33 or 34 or 35 or 36 or 37	3,130,241
39	32 and 38	344,031
40	25 or 39	376,662
41	exp psychosexual behavior/	275,139
42	exp sexually transmitted diseases/	54,268
43	(sexually transmitted disease* or std* or STI*).ti.	93,661
44	(sex* adj2 (health* or safe* or unsafe or protect* or risk or risky or high-risk or unprotected or abstinence or behavio?r* or activit*)).ti,ab.	59,045
45	((STI or STIs or STD or STDs or sexually transmitted disease* or sexually transmitted infection* or Chlamydia or gonorrhoea or syphilis) and (prevent* or control* or risk* or reduc*)).ti.	1,437

46	(contraception or contraceptive* or condom* or "HPV vaccine" or "human papillomavirus" or "pre-exposure prophylaxis").ti,ab.	23,583
47	exp adolescent pregnancy/	3,200
48	(sexual adj3 (health* or behavio?r*)).ti,ab.	41,646
49	exp birth control/	12,826
50	reproductive health/	4,845
51	41 or 42 or 43 or 44 or 45 or 46 or 47 or 48 or 49 or 50	436,022
52	16 or 40 or 51	2,119,292
53	exp digital technology/	183,540
54	exp electronic health services/	30,310
55	exp mobile applications/	3,729
56	wearable devices/	1,230
57	exp mobile phones/	8,786
58	videoconferencing/	1,133
59	gamification/	1,167
60	(digital* or online* or internet* or web* or email or mobile or app or apps or browser or virtual).ti.	98,328
61	((Digital* or online or web* or internet or virtual* or email*) adj2 (technolog* or intervention* or deliver* or based or support*)).ab.	44,626
62	(smartphone* or (smart adj phone*)).tw.	9,738
63	(mobile adj (device* or health or phone*)).tw.	10,514
64	text messag*.tw.	3,469
65	(e-health or ehealth or m-health or mhealth).tw.	5,814
66	(SMS or MMS).tw.	2,672
67	wearable*.tw.	3,140
68	(facebook or twitter or snapchat or facetime or Tiktok or youtube or whatsapp or Instagram or reddit or blog or blogs or blogging or webinar* or podcast*).tw.	20,328
69	(social adj (media or network*)).tw.	60,634
70	(smart adj (device* or tech*)).tw.	530
71	(electronic adj2 device*).tw.	1,400
72	((mobile or online or tablet or computer or phone) adj2 (app or apps)).ab.	2,479
73	(Zoom or skype or "video conferenc*").tw.	4,061
74	virtual reality/	13,022
75	(virtual adj2 (realit* or world* or environment*)).tw.	17,378
76	3d vision.tw.	52
77	Gamification.tw.	1,684
78	53 or 54 or 55 or 56 or 57 or 58 or 59 or 60 or 61 or 62 or 63 or 64 or 65 or 66 or 67 or 68 or 69 or 70 or 71 or 72 or 73 or 74 or 75 or 76 or 77	297,119
79	52 and 78	108,811

80	(metasynthesis or "systematic review").md. or "Systematic Review"/ or "systematic reviews as topic".mh. or ("Cochrane Database of Systematic Reviews" or evidence report technology assessment or evidence report technology assessment summary).jn. or (((((comprehensive or comprehensively) adj (analysis or review or reviewed)) or ((literature or scoping) adj (search or searches))).ti,ab,id. not "narrative review".ti.) and (database or databases or cinahl or cochrane or embase or psycinfo or pubmed or medline or scopus or (web adj1 science) or ((bibliographic or literature) adj (review or reviews)) or (((electronic adj (database or databases)) or (databases adj3 searched)) and (eligibility or excluded or exclusion or included or inclusion))).ti,ab,id.) or (((comparative adj effectiveness) and (effectiveness adj review)) or ((critical adj interpretive) and ((interpretive adj review) or (interpretive adj synthesis)))).ti,ab,id. or ((diagnostic adj test) and ((accuracy adj review) or (accuracy adj reviews) or (accuracy adj studies) or (accuracy adj study)) and (meta-analysis or scoping or systematic)).ti,ab,id. or ((evidence adj assessment) and GRADE).ti,ab,id. or ((evidence adj2 gap) and (gap adj map)).ti,ab,id. or ((evidence adj mapping) or (evidence adj review) or (exploratory adj review) or (framework adj synthesis) or (mapping adj review)).ti,ab,id. or ((meta adj (epidemiological or ethnographic or ethnography or interpretation or narrative or review or study or synthesis or summary or theory)) or metaethnographic or metaethnography or metasynthesis).ti,ab,id. or ((methodological or methodology) adj1 review).ti,ab,id. or ((mixed adj methods) and (methods adj1 (review or synthesis))).ti,ab,id. or ((narrative adj1 synthesis) or (overview adj4 reviews) or ("PRISMA" adj4 (guideline or guidelines or preferred or reporting or requirements)) or (PRISMA adj "P")).ti,ab,id. or (((prognostic or psychometric) adj1 review) or ((qualitative adj (evidence or research)) and ((evidence or research) adj synthesis))).ti,ab,id. or (((rapid adj evidence) and (evidence adj assessment)) or (rapid adj realist) or (rapid adj2 (review or reviews)) or (realist adj2 (review or reviews or syntheses or synthesis))).ti,ab,id. or (((review adj economic) and (economic adj1 (evaluation or evaluations))) or ((scoping or systematic) adj2 (review or reviews or studies or study))).ti,ab,id. or ((review adj1 reviews) or ((systematic adj evidence) and (evidence adj map)) or (systematic adj2 mapping) or (systematic adj2 literature) or (systematic adj2 (Embase or Medline or PsycInfo or PubMed)) or (systematic adj2 (review or reviews)) or ((systematical or systematically) adj2 (review or reviewed reviews)) or (systematically adj identified) or (systematized adj review) or (umbrella adj (review or reviews))).ti,ab,id. or "Meta-Analysis".md. or "meta-analysis"/ or "meta-analysis as topic".mh. or "Network Meta-Analysis".mh. or (meta adj2 (analyse or analyser or analyses or analysis or analytic or analytical or analytics or analyze or analyzed or analyzes)).ti,ab,id. or (metaanalyse or Metaanalysen or metaanalyser or metaanalyses or metaanalysis* or metaanalytic or metaanalytical or metaanalytics or metaanalyze or metaanalyzed or metaanalyzes).ti,ab,id. or (network adj1 (meta or metaanalyses or metaanalysis or metaregression)).ti,ab,id. or (systematic and ((meta adj regression) or metagression)).ti,ab,id.	135,778
81	79 and 80	4,310
82	limit 81 to yr="2022 -Current"	1,722
83	meta-analysis/ or systematic review/ or systematic reviews as topic/ or meta-analysis as topic/ or "meta analysis (topic)"/ or "systematic review (topic)"/ or exp technology assessment, biomedical/ or network meta-analysis/	903

84	((systematic* adj3 (review* or overview*)) or (methodologic* adj3 (review* or overview*))).ti,ab.	67,250
85	((quantitative adj3 (review* or overview* or synthes*)) or (research adj3 (integrati* or overview*))).ti,ab.	12,214
86	((integrative adj3 (review* or overview*)) or (collaborative adj3 (review* or overview*)) or (pool* adj3 analy*)).ti,ab.	7,447
87	(data synthes* or data extraction* or data abstraction*).ti,ab.	4,751
88	(handsearch* or hand search*).ti,ab.	1,766
89	(mantel haenszel or peto or der simonian or dersimonian or fixed effect* or latin square*).ti,ab.	7,118
90	(met analy* or metanaly* or technology assessment* or HTA or HTAs or technology overview* or technology appraisal*).ti,ab.	1,176
91	(meta regression* or metaregression*).ti,ab.	3,255
92	(meta-analy* or metaanaly* or systematic review* or biomedical technology assessment* or bio-medical technology assessment*).mp,hw.	98,227
93	(medline or cochrane or pubmed or medlars or embase or cinahl).ti,ab,hw.	43,909
94	(comparative adj3 (efficacy or effectiveness)).ti,ab.	2,590
95	(outcomes research or relative effectiveness).ti,ab.	4,180
96	((indirect or indirect treatment or mixed-treatment or bayesian) adj3 comparison*).ti,ab.	583
97	(meta-analysis or systematic review).md.	80,071
98	(multi* adj3 treatment adj3 comparison*).ti,ab.	54
99	(mixed adj3 treatment adj3 (meta-analy* or metaanaly*)).ti,ab.	19
100	umbrella review*.ti,ab.	438
101	(multi* adj2 paramet* adj2 evidence adj2 synthesis).ti,ab.	2
102	(multiparamet* adj2 evidence adj2 synthesis).ti,ab.	6
103	(multi-paramet* adj2 evidence adj2 synthesis).ti,ab.	2
104	83 or 84 or 85 or 86 or 87 or 88 or 89 or 90 or 91 or 92 or 93 or 94 or 95 or 96 or 97 or 98 or 99 or 100 or 101 or 102 or 103	155,074
105	79 and 104	4,745
106	limit 105 to yr="2022 -Current"	1,783
107	82 or 106	1,986

e) Web of Science

Date of search: 20th of January 2025
Search period: 1946 to 17th of January 2025

#	Query	Results from 21 Jan 2025
1	TS=(DIGITAL INTERVENTION) AND TI=(depress* OR anxious* OR anxiet* OR emotion* OR mood OR resilience OR self-esteem OR mindful* OR cope OR coping OR cognitive* OR lifestyles OR "life skills" OR stress* OR psychosocial OR "psychological* distress*" OR cannabis OR marijuana OR drug* OR substance* OR polysubstance OR polydrug OR "poly-	347

	drug" OR "legal high*" OR cannabis OR weed OR marijuana OR skunk OR cocaine OR stimulant* OR ketamine OR pincers OR MDMA OR Molly OR ecstasy OR hobbing OR "hot boxing" OR hallucinogen* OR inhalant* OR psychoactive* OR "psycho active*" OR drink* OR alcohol* OR beer* OR wine* OR liqo?r* OR spirits OR alcopop* OR smoking OR smoke* OR tobacco*OR nicotine OR cigar? OR cigs OR cigarette* OR cigarilloi* OR "hand roll*" OR handroll* OR rollins OR "roll up*" OR "roll-up*" OR rollup* OR vape OR vaping OR ecig* OR e-cig* OR "e cig*" OR STI OR STIs OR STD OR STDs OR sexually transmitted disease* OR sexually transmitted infection* OR Chlamydia OR gonorrhoea OR syphilis OR contraception OR contraceptive* OR condom* OR "HPV vaccine" OR "human papillomavirus" OR "pre-exposure prophylaxis" OR sex*) REFINED BY REVIEW ARTICLE AND DATE 2022-PRESENT	

f) Scopus

Date of search: 21st of January, 2025
Search period: 1946 to 17th of January, 2025

#	Query	Results from 21 Jan 2025
1	INDEXTERMS ("digital intervention") OR TITLE("digital intervention*" OR "digital technolog*" OR app OR apps OR virtual OR facebook OR twitter OR snapchat OR facetime OR Tiktok OR youtube OR whatsapp OR Instagram OR reddit OR blog OR blogs OR blogging OR webinar* OR podcast* OR gamification) AND PUBYEAR > 2021 AND PUBYEAR < 2026 AND (LIMIT-TO (SRCTYPE,"j")) AND (LIMIT-TO (DOCTYPE,"re")) AND (LIMIT-TO (EXACTKEYWORD,"Adolescent") OR LIMIT-TO (EXACTKEYWORD,"Young Adult") OR LIMIT-TO (EXACTKEYWORD,"Child") OR LIMIT-TO (EXACTKEYWORD,"Adolescents") OR LIMIT-TO (EXACTKEYWORD,"Higher Education"))LIMIT TO REVIEW AND DATE 2022-2025	221

Search strings – phase 2 search for randomised trials

a) Summary

Mental Health

Source	Date	Number of hits
CENTRAL	25/05/25	2849
PsycINFO (Ovid)	25/05/25	706
**Total**		3,555
**After deduplication**		3,366

Substance Use

Source	Date	Number of hits
CENTRAL	25/05/25	1277
PsycINFO (Ovid)	25/05/25	212
**Total**		1489
**After deduplication**		1398

Sexual Health

Source	Date	Number of hits
CENTRAL	25/05/25	852
PsycINFO (Ovid)	25/05/25	149
**Total**		1001
**After deduplication**		954

b) Database: CENTRAL

Date Run: 25/05/2025

#	Query	Results from 25 May 2025
#1	MeSH descriptor: [Mental Health] explode all trees	3428
#2	MeSH descriptor: [Mindfulness] explode all trees	2467
#3	MeSH descriptor: [Resilience, Psychological] explode all trees	546
#4	MeSH descriptor: [Depression] explode all trees	19096
#5	MeSH descriptor: [Anxiety] explode all trees	13232
#6	MeSH descriptor: [Stress, Psychological] explode all trees	9380
#7	MeSH descriptor: [Psychological Well-Being] explode all trees	69
#8	(mental* or psych* or mood*):ti	47941
#9	(mental* NEAR/3 health*):ab	30083
#10	(depress* or anxious* or anxiet* or emotion* or mood or resilience or self-efficacy or self-esteem or mindful* or cope or coping or cognitive* or lifeskills or "life skills" or stress* or psychosocial or psychological* NEXT distress* or self-harm* OR self NEXT harm*):ti	134189
#11	(well-being or well being or wellbeing):ti,ab	47051
#12	((social or emotional) NEAR/1 (skills or support or network*)):ti,ab	12639
#13	((depress* or anxiet* or self-harm* or self NEXT harm*) NEAR/5 (symptom* or prevent*)):ab	34692
#14	#1 OR #2 OR #3 OR #4 OR #5 OR #6 OR #7 OR #8 OR #9 OR #10 OR #11 OR #12 OR #13	235162
#15	MeSH descriptor: [Cannabis] explode all trees	649
#16	MeSH descriptor: [Marijuana Use] explode all trees	486
#17	MeSH descriptor: [Drinking Behavior] explode all trees	6043
#18	MeSH descriptor: [Alcoholic Beverages] explode all trees	722
#19	MeSH descriptor: [Smoking] explode all trees	8491
#20	MeSH descriptor: [Smoking Cessation] explode all trees	5836
#21	MeSH descriptor: [Illicit Drugs] explode all trees	326
#22	(alcohol* or beer* or wine* or liqo*r* or spirits or alcopop* or drinking):ti	18719
#23	(smoking or smoke* or tobacco* or nicotine or cigar? or cigs or cigarette* or cigarilloi* or hand NEXT roll* or handroll* or rollies or roll NEXT up* or roll-up* or rollup* or vape or vaping or ecig* or e-cig* or E NEXT cig*):ti,ab	48508
#24	(Drug* or substance* or polysubstance or polydrug or "poly-drug" or legal NEXT high* or cannabis or weed or marijuana or skunk or cocaine or stimulant* or ketamine or pingers or MDMA or Molly or ecstasy or hotboxing or "hot boxing" or hallucinogen* or inhalant* or psychoactive* or psycho NEXT active*):ti,ab	289926
#25	#15 OR #16 OR #17 #18 OR #19 OR #20 OR #21 OR #22 OR #23 OR #24	343399
#26	MeSH descriptor: [Sexually Transmitted Diseases] explode all trees	21180
#27	(sexually transmitted or std* or STI*):ti	45526
#28	((sex* or intercourse) NEAR/3 (health* or safe* or unsafe or protect* or risk or risky or high-risk or unprotected or abstinence or behavio?r* or activit*)):ti,ab	10800
#29	((STI or STIs or STD or STDs or sexually transmitted disease* or sexually transmitted infection* or Chlamydia or gonorrhoea or syphilis) and (prevent* or control* or risk* or reduc* or public health or health promotion)):ti,ab	4933
#30	(contraception or contraceptive* or condom* or "HPV vaccine" or "human papillomavirus" or "pre-exposure prophylaxis"):ti,ab	23819
#31	MeSH descriptor: [Sexual Behavior] explode all trees	4862
#32	MeSH descriptor: [Pregnancy in Adolescence] explode all trees	285
#33	MeSH descriptor: [Contraception] explode all trees	825
#34	MeSH descriptor: [Reproductive Health] explode all trees	178
#35	((unwanted OR prevent*) NEAR/2 pregnan*):ti,ab	1595
#36	(family planning OR reproductive health):ti	890
#37	#26 OR #27 OR #28 OR #29 OR #30 OR #31 OR #32 OR #33 OR #34 OR #35 OR #36	96897
#38	MeSH descriptor: [Digital Technology] explode all trees	26

#39	MeSH descriptor: [Digital Health] explode all trees	47
#40	MeSH descriptor: [Mobile Applications] explode all trees	2352
#41	MeSH descriptor: [Wearable Electronic Devices] explode all trees	1085
#42	MeSH descriptor: [Cell Phone] explode all trees	3658
#43	MeSH descriptor: [Videoconferencing] explode all trees	363
#44	MeSH descriptor: [Gamification] explode all trees	28
#45	(digital* or online* or internet* or web* or email or mobile or app or apps or browser or virtual or computeri?ed):ti	40555
#46	((Digital* or online or "on line" or web* or internet or virtual* or email* or computer or phone or technolog*) NEAR/2 (feedback or technolog* or intervention* or deliver* or based or support*)):ab	60812
#47	(smartphone* or (smart NEAR/1 phone*)):ti,ab	10360
#48	(mobile NEAR/1 (device* or health or phone*)):ti,ab	7752
#49	text messag*:ti,ab	8145
#50	(e-health or ehealth or m-health or mhealth):ti,ab	4941
#51	(SMS or MMS):ti,ab	4059
#52	wearable*:ti,ab	3009
#53	(facebook or twitter or snapchat or facetime or Tiktok or youtube or whatsapp or Instagram or reddit or blog or blogs or blogging or webinar* or podcast*):ti,ab	3682
#54	(social NEAR/1 (media or network*)):ti,ab	5338
#55	(smart NEAR/1 (device* or tech*)):ti,ab	367
#56	(electronic NEAR/2 device*):ti,ab	1234
#57	((mobile or online or tablet or computer or phone) NEAR/2 (app or apps)):ab	3188
#58	(Zoom or skype or video NEXT conferenc*):ti,ab	2319
#59	MeSH descriptor: [Virtual Reality] explode all trees	1392
#60	(virtual NEAR/2 (realit* or world* or environment*)):ti,ab	8399
#61	3d vision:ti,ab	333
#62	Gamification:ti,ab	552
#63	#38 OR #39 OR #40 OR #41 OR #42 OR #43 OR #44 OR #45 OR #46 OR #47 OR #48 OR #49 OR #50 OR #51 OR #52 OR #53 OR #54 OR #55 OR #56 OR #57 OR #58 OR #59 OR #60 OR #61 OR #62	112069
#64	(underage* OR under-age* OR under NEXT age* OR teen* OR youth* OR adolescen* OR juvenile* OR youngster* OR emerging NEXT adult* OR first-grader* OR second-grader* OR third-grader* OR fourth-grader* OR fifth-grader* OR sixth-grader* OR seventh-grader* OR highschool* OR high NEXT school* OR college* OR universit* NEAR/2 student*):ti,ab	75658
#65	(young NEAR/2 (adult* OR person* OR individual* OR people* OR population* OR man OR men OR wom*n)):ti,ab	24967
#66	((secondary OR high*) NEAR/2 (school* OR education)):ti,ab	8974
#67	MeSH descriptor: [Young Adult] explode all trees	98597
#68	MeSH descriptor: [Adolescent] explode all trees	136477
#69	#64 OR #65 OR #66 OR #67 OR #68	255284
#70	#63 AND #69	19376
#71	#14 AND #70 with Cochrane Library publication date Between Feb 2022 and Jun 2025	2913<br>(2849 RCTs)
#72	#25 AND #70 with Cochrane Library publication date Between Nov 2020 and Jun 2025	1345<br>(1277 RCTs)
#73	#37 AND #70 with Cochrane Library publication date Between Aug 2020 and Jun 2025	860<br>(852 RCTs)

c) Database: APA PsycInfo

Date search run: 25/05/2025

#	Query	Results from 25 May 2025
1	mental disorders/	103,492
2	exp mental health/	109,646
3	mindfulness/	14,416
4	"resilience (psychological)"/	26,819

5	"depression (emotion)"/	27,515
6	exp anxiety/	102,306
7	exp stress/	164,183
8	public mental health/	1,056
9	exp *well being/	57,879
10	(mental* or psych* or ?mood*).ti.	663,859
11	(mental* adj3 health*).ab.	259,667
12	(depress* or anxious* or anxiet* or emotion* or mood or resilience or self-efficacy or self-esteem or mindful* or cope or coping or cognitive* or lifeskills or "life skills" or stress* or psychosocial or "psychological* distress* self-harm*" or self NEXT harm*).ti.	626,384
13	(well-being or well being or wellbeing).ti,ab.	145,809
14	((social or emotional) adj1 (skills or support or network*)).ti,ab.	117,213
15	((depress* or anxiet* or self-harm* or self NEXT harm*) adj5 (symptom* or prevent*)).ab.	124,992
16	1 or 2 or 3 or 4 or 5 or 6 or 7 or 8 or 9 or 10 or 11 or 12 or 13 or 14 or 15	1,577,368
17	binge drinking/ or social drinking/ or underage drinking/	5,623
18	marijuana/	3,988
19	"cannabis use"/	4,582
20	"legal high*".tw.	200
21	exp drug abstinence/	20,503
22	exp "substance use prevention"/	5,453
23	exp alcoholic beverages/	3,694
24	"alcohol use attitudes"/	3,315
25	exp *tobacco smoking/	33,865
26	(Drug* or "substance use" or polysubstance or polydrug or "poly-drug" or "legal high*" or cannabis or weed or marijuana or skunk or cocaine or stimulant* or ketamine or pingers or MDMA or Molly or ecstasy or hotboxing or "hot boxing" or hallucinogen* or inhalant* or psychoactive* or "psycho active*").ti,ab.	306,964
27	(smoking or smoke* or tobacco* or nicotine or cigar? or cigs or cigarette* or cigarilloi* or "hand roll*" or handroll* or rollies or "roll up*" or "roll-up*" or rollup* or vape or vaping or ecig* or e-cig* or "e cig*").ti,ab.	87,003
28	(drink* or alcohol* or beer* or wine* or liqo?r* or spirits or alcopop*).ti,ab.	174,954
29	17 or 18 or 19 or 20 or 21 or 22 or 23 or 24 or 25 or 26 or 27 or 28	475,858
30	exp psychosexual behavior/	279,446
31	exp sexually transmitted diseases/	54,938
32	(sexually transmitted disease* or std* or STI*).ti.	94,751
33	((sex* or intercourse) adj2 (health* or safe* or unsafe or protect* or risk or risky or high-risk or unprotected or abstinence or behavio?r* or activit*)).ti,ab.	60,044
34	((STI or STIs or STD or STDs or sexually transmitted disease* or sexually transmitted infection* or Chlamydia or gonorrhoea or syphilis) and (prevent* or control* or risk* or reduc*)).ti.	1,449
35	(contraception or contraceptive* or condom* or "HPV vaccine" or "human papillomavirus" or "pre-exposure prophylaxis").ti,ab.	23,904
36	exp adolescent pregnancy/	3,217
37	(sexual adj3 (health* or behavio?r*)).ti,ab.	42,143
38	exp birth control/	12,961
39	reproductive health/	4,957
40	"unwanted pregnan*".ti,ab.	987
41	(family planning or reproductive health).ti.	2,541
42	30 or 31 or 32 or 33 or 34 or 35 or 36 or 37 or 38 or 39 or 40 or 41	442,655
43	exp digital technology/	245,652
44	exp electronic health services/	31,658
45	exp mobile applications/	3,971
46	wearable devices/	1,318
47	exp mobile phones/	9,003

48	videoconferencing/	1,176
49	gamification/	1,230
50	(digital* or online* or on line or internet* or web* or email or mobile or app or apps or browser or virtual or computeri?sed).ti.	102,801
51	((Digital* or online or on line or web* or internet or virtual* or email* or computer* or phone or technology or text) adj2 (feedback or technolog* or intervention* or deliver* or based or support*)).ab.	157,356
52	(smartphone* or (smart adj phone*)).tw.	10,111
53	(mobile adj (device* or health or phone*)).tw.	10,836
54	text messag*.tw.	3,534
55	(e-health or ehealth or m-health or mhealth).tw.	6,081
56	(SMS or MMS).tw.	2,713
57	wearable*.tw.	3,305
58	(facebook or twitter or snapchat or facetime or Tiktok or youtube or whatsapp or Instagram or reddit or blog or blogs or blogging or webinar* or podcast*).tw.	20,942
59	(social adj (media or network*)).tw.	62,461
60	(smart adj (device* or tech*)).tw.	555
61	(electronic adj2 device*).tw.	1,436
62	((mobile or online or tablet or computer or phone) adj2 (app or apps)).ab.	2,570
63	(Zoom or skype or "video conferenc*").tw.	4,309
64	virtual reality/	13,489
65	(virtual adj2 (realit* or world* or environment*)).tw.	18,005
66	3d vision.tw.	52
67	Gamification.tw.	1,786
68	43 or 44 or 45 or 46 or 47 or 48 or 49 or 50 or 51 or 52 or 53 or 54 or 55 or 56 or 57 or 58 or 59 or 60 or 61 or 62 or 63 or 64 or 65 or 66 or 67	427,075
69	(((underage* or under-age* or under NEXT age* or teen* or youth* or adolescen* or juvenile* or (young adj2 (adult* or person* or individual* or people* or population* or man or men or wom?n)) or youngster* or first-grader* or second-grader* or third-grader* or fourth-grader* or fifth-grader* or sixth-grader* or seventh-grader* or highschool* or college* or university*) adj2 student*) or ((secondary or high*) adj2 (school* or education))).ti,ab.	279,940
70	emerging adulthood/	11,270
71	adolescent.hw.	119,985
72	69 or 70 or 71	393,904
73	Experiment Controls/ or Placebo/ or Clinical Trials/ or Randomized Controlled Trials/ or Randomized Clinical Trials/	21,073
74	Clinical trial.md.	46,752
75	(random* or sham or placebo*).ti,ab,id,hw,mf.	311,915
76	((singl* or doubl*) adj (blind* or dumm* or mask*)).ti,ab,id,hw,mf.	38,736
77	((tripl* or trebl*) adj (blind* or dumm* or mask*)).ti,ab,id,hw,mf.	146
78	(control* adj3 (study or studies or trial* or group*)).ti,ab,id,hw,mf.	243,893
79	(clinical adj3 (study or studies or trial*)).ti,ab,id,hw,mf.	88,545
80	(Nonrandom* or non random* or non-random* or quasi-random* or quasirandom*).ti,ab,id,hw,mf.	7,344
81	(phase adj6 (study or studies or trial*)).ti,ab,id,hw,mf.	16,461
82	((crossover or cross-over) adj3 (study or studies or trial*)).ti,ab,id,hw,mf.	8,882
83	((multicent* or multi-cent*) adj3 (study or studies or trial*)).ti,ab,id,hw,mf.	11,501
84	allocated.ti,ab,hw,mf.	14,326
85	((open label or open-label) adj5 (study or studies or trial*)).ti,ab,id,hw,mf.	5,655
86	((equivalence or superiority or non-inferiority or noninferiority) adj3 (study or studies or trial*)).ti,ab,id,hw,mf.	1,383
87	(pragmatic study or pragmatic studies).ti,ab,id,hw,mf.	167
88	((pragmatic or practical) adj3 trial*).ti,ab,id,hw,mf.	1,352
89	((quasiexperimental or quasi-experimental) adj3 (study or studies or trial*)).ti,ab,id,hw,mf.	8,266
90	trial.ti,id.	49,769

91	or/73-90	551,926
92	68 and 72 and 91	5,095
93	16 and 92	1,842
94	29 and 92	718
95	42 and 92	394
96	limit 93 to up=20220201-20250601	706
97	limit 94 to up=20201101-20250601	212
98	limit 95 to up=20200801-20250601	149

Main trial reports included

Aboody, D., Siev, J., & Doron, G. (2020). Building resilience to body image triggers using brief cognitive training on a mobile application: A randomized controlled trial. Behav Res Ther, 134, 103723. https://doi.org/10.1016/j.brat.2020.103723

Ampatzoglou, A., Carballo-Marquez, A., Garcia-Casanovasa, A., Rojas, J., Fernandez-Capo, M., Gamiz-Sanfeliu, M., Garolera, M., & Porras-Garcia, B. (2024). Exploring the Impact of Gamified Virtual Reality Cognitive Training on Improving Anxiety and Depressive Symptoms in Spanish Adolescent Girls. Annual review of cybertherapy and telemedicine,(22), 106-112.

Bauermeister, J., Choi, S. K., Bruehlman-Senecal, E., Golinkoff, J., Taboada, A., Lavra, J., Ramazzini, L., Dillon, F., & Haritatos, J. (2022). An Identity-Affirming Web Application to Help Sexual and Gender Minority Youth Cope With Minority Stress: Pilot Randomized Controlled Trial. J Med Internet Res, 24(8), e39094. https://doi.org/10.2196/39094

Bauermeister, J. A., Horvath, K. J., Lin, W. Y., Golinkoff, J. M., Claude, K. F., Dowshen, N., Castillo, M., Sullivan, P. S., Paul, M., Hightow-Weidman, L., & Stephenson, R. (2024). Enhancing routine HIV and STI testing among young men who have sex with men: primary outcomes of the get connected clinical randomized trial (ATN 139). BMC public health, 24(1), 1072. https://doi.org/10.1186/s12889-024-18522-w

Bauermeister, J. A., Tingler, R. C., Demers, M., Connochie, D., Gillard, G., Shaver, J., Chavanduka, T., & Harper, G. W. (2019). Acceptability and Preliminary Efficacy of an Online HIV Prevention Intervention for Single Young Men Who Have Sex with Men Seeking Partners Online: The myDEx Project. AIDS Behav, 23(11), 3064-3077. https://doi.org/10.1007/s10461-019-02426-7

Bennett, A. T., Patel, D. A., Carlos, R. C., Zochowski, M. K., Pennewell, S. M., Chi, A. M., & Dalton, V. K. (2015). Human Papillomavirus Vaccine Uptake After a Tailored, Online Educational Intervention for Female University Students: A Randomized Controlled Trial. J Womens Health (Larchmt), 24(11), 950-957. https://doi.org/10.1089/jwh.2015.5251

Biello, K. B., Daddario, S. R., Hill-Rorie, J., Futterman, D., Sullivan, P. S., Hightow-Weidman, L., Jones, J., Mimiaga, M. J., & Mayer, K. H. (2022). Uptake and Acceptability of MyChoices: Results of a Pilot RCT of a Mobile App Designed to Increase HIV Testing and PrEP Uptake Among Young American MSM. AIDS and Behavior, 26(12), 3981-3990. https://doi.org/10.1007/s10461-022-03724-3

Biello, K. B., Horvitz, C., Mullin, S., Mayer, K. H., Scott, H., Coleman, K., Dormitzer, J., Norelli, J., Hightow-Weidman, L., Sullivan, P., Mimiaga, M. J., Buchbinder, S., Bojan, K., Futterman, D., Emmanuel, P., Liu, A., & of the iTech Team, o. b. (2020). HIV self-testing and STI self-collection via mobile apps: experiences from two pilot randomized controlled trials of young men who have sex with men. mHealth, 7. https://mhealth.amegroups.org/article/view/50249

Biello, K. B., Mayer, K. H., Scott, H., Valente, P. K., Hill-Rorie, J., Buchbinder, S., Ackah-Toffey, L., Sullivan, P. S., Hightow-Weidman, L., & Liu, A. Y. (2025). The Effects of MyChoices and LYNX Mobile Apps on HIV Testing and Pre-Exposure Prophylaxis Use by Young US Sexual Minority Men: Results From a National Randomized Controlled Trial. JMIR Public Health Surveill, 11, e63428. https://doi.org/10.2196/63428

Bull, S., Devine, S., Schmiege, S. J., Pickard, L., Campbell, J., & Shlay, J. C. (2016). Text Messaging, Teen Outreach Program, and Sexual Health Behavior: A Cluster Randomized Trial. Am J Public Health, 106(S1), S117-S124. https://doi.org/10.2105/AJPH.2016.303363

Bull, S. S., Levine, D. K., Black, S. R., Schmiege, S. J., & Santelli, J. (2012). Social Media Delivered Sexual Health Intervention: A Cluster Randomized Controlled Trial. American Journal of Preventive Medicine, 43(5), 467-474. https://doi.org/10.1016/j.amepre.2012.07.022

Carvalho, T., Alvarez, M.-J., Pereira, C., & Schwarzer, R. (2016). Stage-Based Computer-Delivered Interventions to Increase Condom Use in Young Men. International Journal of Sexual Health, 28(2), 176-186. https://doi.org/10.1080/19317611.2016.1158764

Chernick, L. S., Bugaighis, M., Hochster, D., Daylor, V., Gorroochurn, P., Schnall, R., Stockwell, M. S., & Bell, D. (2025). A Randomized Controlled Trial of a Digital Intervention to Improve the Sexual Health of Adolescent and Young Adult Male Emergency Department Patients. Journal of adolescent health, 76(1), 140-147. https://doi.org/10.1016/j.jadohealth.2024.08.020

Chernick, L. S., Santelli, J., Stockwell, M. S., Gonzalez, A., Ehrhardt, A., Thompson, J. L. P., Leu, C. S., Bakken, S., Westhoff, C. L., & Dayan, P. S. (2022). A multi-media digital intervention to improve the sexual and reproductive health of female adolescent emergency department patients. Acad Emerg Med, 29(3), 308-316. https://doi.org/10.1111/acem.14411

Chernick, L. S., Stockwell, M. S., Wu, M., Castaño, P. M., Schnall, R., Westhoff, C. L., Santelli, J., & Dayan, P. S. (2017). Texting to Increase Contraceptive Initiation Among Adolescents in the Emergency Department. Journal of adolescent health, 61(6), 786-790. https://doi.org/10.1016/j.jadohealth.2017.07.021

Christensen, J. L., Miller, L. C., Appleby, P. R., Corsbie-Massay, C., Godoy, C. G., Marsella, S. C., & Read, S. J. (2013). Reducing shame in a game that predicts HIV risk reduction for young adult MSM: a randomized trial delivered nationally over the Web. J Int AIDS Soc, 16(3 Suppl 2), 18716. https://doi.org/10.7448/IAS.16.3.1871618716

Cordova, D., Munoz-Velazquez, J., Mendoza Lua, F., Fessler, K., Warner, S., Delva, J., Adelman, N., Fernandez, A., & Bauermeister, J. (2020). Pilot Study of a Multilevel Mobile Health App for Substance Use, Sexual Risk Behaviors, and Testing for Sexually Transmitted Infections and HIV Among Youth: Randomized Controlled Trial. JMIR Mhealth Uhealth, 8(3), e16251. https://doi.org/10.2196/16251

Craig Rushing, S., Kelley, A., Bull, S., Stephens, D., Wrobel, J., Silvasstar, J., Peterson, R., Begay, C., Ghost Dog, T., McCray, C., Love Brown, D., Thomas, M., Caughlan, C., Singer, M., Smith, P., & Sumbundu, K. (2021). Efficacy of an mHealth Intervention (BRAVE) to Promote Mental Wellness for American Indian and Alaska Native Teenagers and Young Adults: Randomized Controlled Trial. JMIR Ment Health, 8(9), e26158. https://doi.org/10.2196/26158

Egan, J. E., Corey, S. L., Henderson, E. R., Abebe, K. Z., Louth-Marquez, W., Espelage, D., Hunter, S. C., DeLucas, M., Miller, E., Morrill, B. A., Hieftje, K., Sang, J. M., Friedman, M. S., & Coulter, R. W. S. (2021). Feasibility of a Web-Accessible Game-Based Intervention Aimed at Improving Help Seeking and Coping Among Sexual and Gender Minority Youth: Results From a Randomized

Controlled Trial. Journal of adolescent health, 69(4), 604-614. https://doi.org/10.1016/j.jadohealth.2021.03.027

Fang, L., Schinke, S. P., & Cole, K. C. A. (2010). Preventing Substance Use Among Early Asian–American Adolescent Girls: Initial Evaluation of a Web-based, Mother–Daughter Program. Journal of adolescent health, 47(5), 529-532. https://doi.org/10.1016/j.jadohealth.2010.03.011

Fiellin, L. E., Hieftje, K. D., Pendergrass, T. M., Kyriakides, T. C., Duncan, L. R., Dziura, J. D., Sawyer, B. G., Mayes, L., Crusto, C. A., Forsyth, B. W. C., & Fiellin, D. A. (2017). Video Game Intervention for Sexual Risk Reduction in Minority Adolescents: Randomized Controlled Trial. J Med Internet Res, 19(9), e314. https://doi.org/10.2196/jmir.8148

Gerend, M. A., Madkins, K., Crosby, S., Korpak, A. K., Phillips, G. L., II, Bass, M., Houlberg, M., & Mustanski, B. (2020). Evaluation of a Text Messaging-Based Human Papillomavirus Vaccination Intervention for Young Sexual Minority Men: Results from a Pilot Randomized Controlled Trial. Annals of Behavioral Medicine, 55(4), 321-332. https://doi.org/10.1093/abm/kaaa056

Gilmore, A. K., Fortson, K., Mullican, K. N., García-Ramírez, G., Hutchins, A., Bartlett, A. M., Gooding, H. C., Wallis, E., Levy, S., Ruggiero, K. J., Kaysen, D., Danielson, C. K., Platner, R., Hartman, A., & Self-Brown, S. (2023). An eHealth Prevention Program for Substance Use, Sexual Assault, and Sexual Risk Behaviors for Adolescents in Primary Care: Pilot Feasibility Randomized Controlled Trial of Teen Well Check. JMIR Form Res, 7, e50833. https://doi.org/10.2196/50833

Glass, N., Bloom, T., Alexander, K. A., Emezue, C., Olawole, W., Clough, A., Turner, R., & Perrin, N. (2024). Effectiveness of the myPlan Teen App, a Digital Healthy Relationship and Safety Planning Intervention With Adolescent Aged 15–17 Years. Journal of adolescent health, 75(4), 665-672. https://doi.org/https://doi.org/10.1016/j.jadohealth.2024.06.014

Glass, N. E., Clough, A., Messing, J. T., Bloom, T., Brown, M. L., Eden, K. B., Campbell, J. C., Gielen, A., Laughon, K., Grace, K. T., Turner, R. M., Alvarez, C., Case, J., Barnes-Hoyt, J., Alhusen, J., Hanson, G. C., & Perrin, N. A. (2022). Longitudinal Impact of the myPlan App on Health and Safety Among College Women Experiencing Partner Violence. J Interpers Violence, 37(13-14), NP11436-NP11459. https://doi.org/10.1177/0886260521991880

Hebert, L. E., Hill, B. J., Quinn, M., Holl, J. L., Whitaker, A. K., & Gilliam, M. L. (2018). Mobile contraceptive application use in a clinical setting in addition to standard contraceptive counseling: A randomized controlled trial. Contraception, 98(4), 281-287. https://doi.org/10.1016/j.contraception.2018.07.001

Hightow-Weidman, L. B., LeGrand, S., Muessig, K. E., Simmons, R. A., Soni, K., Choi, S. K., Kirschke-Schwartz, H., & Egger, J. R. (2019). A Randomized Trial of an Online Risk Reduction Intervention for Young Black MSM. AIDS and Behavior, 23(5), 1166-1177. https://doi.org/10.1007/s10461-018-2289-9

Jeong, S., Cha, C., Nam, S., & Song, J. (2024). The effects of mobile technology-based support on young women with depressive symptoms: A block randomized controlled trial. Medicine (Baltimore), 103(1), e36748. https://doi.org/10.1097/MD.0000000000036748

Jones, R., Hoover, D. R., & Lacroix, L. J. (2013). A randomized controlled trial of soap opera videos streamed to smartphones to reduce risk of sexually transmitted human immunodeficiency virus (HIV) in young urban African American women. Nursing Outlook, 61(4), 205-215.e203. https://doi.org/10.1016/j.outlook.2013.03.006

Kaufman, C. E., Asdigian, N. L., Reed, N. D., Shrestha, U., Bull, S., Tuitt, N. R., Vossberg, R., Mumby, S., & Sarche, M. (2025). One-month outcomes of a culturally tailored alcohol-exposed pregnancy prevention mobile app among urban Native young women: A randomized controlled trial of Native WYSE CHOICES. Alcohol Clin Exp Res (Hoboken), 49(3), 641-653. https://doi.org/10.1111/acer.70002

Kissinger, P. J., Green, J., Latimer, J., Schmidt, N., Ratnayake, A., Madkour, A. S., Clum, G., Wingood, G. M., DiClemente, R. J., & Johnson, C. (2023). Internet-Delivered Sexually Transmitted Infection and Teen Pregnancy Prevention Program: A Randomized Trial. Sexually Transmitted Diseases, 50(6). https://journals.lww.com/stdjournal/fulltext/2023/06000/internet_delivered_sexually_transmitted_infection.2.aspx

Manlove, J., Cook, E., Whitfield, B., Johnson, M., Martínez-García, G., & Garrido, M. (2020). Short-Term Impacts of Pulse: An App-Based Teen Pregnancy Prevention Program for Black and Latinx Women. Journal of adolescent health, 66(2), 224-232. https://doi.org/10.1016/j.jadohealth.2019.08.017

Martínez-García, G., Ewing, A. C., Olugbade, Y., DiClemente, R. J., & Kourtis, A. P. (2023). Crush: A Randomized Trial to Evaluate the Impact of a Mobile Health App on Adolescent Sexual Health. Journal of adolescent health, 72(2), 287-294. https://doi.org/10.1016/j.jadohealth.2022.09.019

Mason, M., Mennis, J., Way, T., Zaharakis, N., Campbell, L. F., Benotsch, E. G., Keyser-Marcus, L., & King, L. (2016). Text Message Delivered Peer Network Counseling for Adolescent Smokers: A Randomized Controlled Trial. J Prim Prev, 37(5), 403-420. https://doi.org/10.1007/s10935-016-0439-2

Mason, M. J., Campbell, L., Way, T., Keyser-Marcus, L., Benotsch, E., Mennis, J., Zhang, J., King, L., May, J., & Stembridge, D. R. (2015). Development and Outcomes of a Text Messaging Tobacco Cessation Intervention with Urban Adolescents. Substance Abuse, 36(4), 500-506. https://doi.org/10.1080/08897077.2014.987946

Mustanski, B., Garofalo, R., Monahan, C., Gratzer, B., & Andrews, R. (2013). Feasibility, Acceptability, and Preliminary Efficacy of an Online HIV Prevention Program for Diverse Young Men who have Sex with Men: The Keep It Up! Intervention. AIDS and Behavior, 17(9), 2999-3012. https://doi.org/10.1007/s10461-013-0507-z

Mustanski, B., Macapagal, K., Li, D. H., Rodriguez-Diaz, C. E., Saber, R., Matson, M., Moskowitz, D. A., Rendina, H. J., Laber, E., Ryan, D. T., & Newcomb, M. E. (2025). Effectiveness of the smart program: Stepped-care HIV prevention for gay and bisexual adolescent boys. Health Psychol, 44(3), 321-331. https://doi.org/10.1037/hea0001471

Mustanski, B., Parsons, J. T., Sullivan, P. S., Madkins, K., Rosenberg, E., & Swann, G. (2018). Biomedical and Behavioral Outcomes of Keep It Up!: An eHealth HIV Prevention Program RCT. American Journal of Preventive Medicine, 55(2), 151-158. https://doi.org/10.1016/j.amepre.2018.04.026

Patel, P. R., Nandigam, L., Thompson, J., Abacan, A., & Raphael, M. (2024). The Impact of Get It? on Long-Acting Reversible Contraception Use Among Adolescents and Young Adults. Journal of Pediatric and Adolescent Gynecology, 37(2), 156-159. https://doi.org/10.1016/j.jpag.2023.11.002

Prokhorov, A. V., Kelder, S. H., Shegog, R., Murray, N., Peters, R., Jr., Agurcia-Parker, C., Cinciripini, P. M., de Moor, C., Conroy, J. L., Hudmon, K. S., Ford, K. H., & Marani, S. (2008). Impact of A Smoking Prevention Interactive Experience (ASPIRE), an interactive, multimedia smoking prevention and cessation curriculum for culturally diverse high-school students. Nicotine Tob Res, 10(9), 1477-1485. https://doi.org/10.1080/14622200802323183

Reback, C. J., Cain, D., Rusow, J. A., Benkeser, D., Schader, L., Gwiazdowski, B. A., Skeen, S. J., Hannah, M., Belzer, M., Castillo, M., Mayer, K. H., Paul, M. E., Hill-Rorie, J., Johnson, N. D., McAvoy-Banerjea, J., Sanchez, T., Hightow-Weidman, L. B., Sullivan, P. S., & Horvath, K. J. (2024). Technology-Based Interventions, with a Stepped Care Approach, for Reducing Sexual Risk Behaviors and Increasing PrEP Initiation Among Transgender and Gender Expansive Youth and Young Adults. AIDS and Behavior, 28(12), 3956-3969. https://doi.org/10.1007/s10461-024-04513-w

Reiter, P. L., Gower, A. L., Kiss, D. E., Shoben, A. B., Katz, M. L., Bauermeister, J. A., Paskett, E. D., & McRee, A.-L. (2023). Efficacy of the Outsmart HPV Intervention: A Randomized Controlled Trial to Increase HPV Vaccination among Young Gay, Bisexual, and Other Men Who Have Sex with Men. Cancer epidemiology, biomarkers & prevention, 32(6), 760-767. https://doi.org/10.1158/1055-9965.Epi-23-0007

Santa Maria, D., Padhye, N., Businelle, M., Yang, Y., Jones, J., Sims, A., & Lightfoot, M. (2021). Efficacy of a Just-in-Time Adaptive Intervention to Promote HIV Risk Reduction Behaviors Among Young Adults Experiencing Homelessness: Pilot Randomized Controlled Trial. J Med Internet Res, 23(7), e26704. https://doi.org/10.2196/26704

Schinke, S. P., Fang, L., & Cole, K. C. (2009). Computer-delivered, parent-involvement intervention to prevent substance use among adolescent girls. Prev Med, 49(5), 429-435. https://doi.org/10.1016/j.ypmed.2009.08.001

Schinke, S. P., Schwinn, T. M., & Ozanian, A. J. (2005). Alcohol abuse prevention among high-risk youth: computer-based intervention. J Prev Interv Community, 29(1-2), 117-130. https://doi.org/10.1300/J005v29n01_08

Schnall, R., Kuhns, L. M., Pearson, C., Batey, D. S., Bruce, J., Hidalgo, M. A., Hirshfield, S., Janulis, P., Jia, H., Radix, A., Belkind, U., Rodriguez, R. G., & Garofalo, R. (2022). Efficacy of MyPEEPS Mobile, an HIV Prevention Intervention Using Mobile Technology, on Reducing Sexual Risk Among Same-Sex Attracted Adolescent Males: A Randomized Clinical Trial. JAMA Netw Open, 5(9), e2231853. https://doi.org/10.1001/jamanetworkopen.2022.31853

Schnall, R., Scherr, T. F., Kuhns, L. M., Janulis, P., Jia, H., Wood, O. R., Almodovar, M., & Garofalo, R. (2024). Efficacy of the mLab App: a randomized clinical trial for increasing HIV testing uptake using mobile technology. Journal of the American Medical Informatics Association, 32(2), 275-284. https://doi.org/10.1093/jamia/ocae261

Schwartz, J., Bottorff, J. L., Ratner, P. A., Gotay, C., Johnson, K. C., Memetovic, J., & Richardson, C. G. (2014). Effect of web-based messages on girls' knowledge and risk perceptions related to cigarette smoke and breast cancer: 6-month follow-up of a randomized controlled trial. JMIR Res Protoc, 3(3), e53. https://doi.org/10.2196/resprot.3282

Schwinn, T. M., Fang, L., Hopkins, J., & Pacheco, A. R. (2021). Longitudinal Outcomes of a Smartphone Application to Prevent Drug Use Among Hispanic Youth. J Stud Alcohol Drugs, 82(5), 668-677. https://doi.org/10.15288/jsad.2021.82.668

Schwinn, T. M., Schinke, S. P., & Di Noia, J. (2010). Preventing drug abuse among adolescent girls: outcome data from an internet-based intervention. Prev Sci, 11(1), 24-32. https://doi.org/10.1007/s11121-009-0146-9

Schwinn, T. M., Thom, B., Schinke, S. P., & Hopkins, J. (2015). Preventing Drug Use Among Sexual-Minority Youths: Findings From a Tailored, Web-Based Intervention. Journal of adolescent health, 56(5), 571-573. https://doi.org/10.1016/j.jadohealth.2014.12.015

Shen, J., Rubin, A., Cohen, K., Hart, E. A., Sung, J., McDanal, R., Roulston, C., Sotomayor, I., Fox, K. R., & Schleider, J. L. (2023). Randomized evaluation of an online single-session intervention for minority stress in LGBTQ+ adolescents. Internet Interv, 33, 100633. https://doi.org/10.1016/j.invent.2023.100633

Starosta, A. J., Cranston, E., & Earleywine, M. (2016). Safer sex in a digital world: A Web-based motivational enhancement intervention to increase condom use among college women. Journal of American College Health, 64(3), 184-193. https://doi.org/10.1080/07448481.2015.1107835

Stephenson, J., Bailey, J. V., Gubijev, A., D'Souza, P., Oliver, S., Blandford, A., Hunter, R., Shawe, J., Rait, G., Brima, N., & Copas, A. (2020). An interactive website for informed contraception choice: randomised evaluation of Contraception Choices. DIGITAL HEALTH, 6, 2055207620936435. https://doi.org/10.1177/2055207620936435

Tan, R. K. J., Koh, W. L., Le, D., Banerjee, S., Chio, M. T.-W., Chan, R. K. W., Wong, C. M., Tai, B. C., Wong, M. L., Cook, A. R., Chen, M. I. C., & Wong, C. S. (2022). Effect of a Popular Web Drama Video Series on HIV and Other Sexually Transmitted Infection Testing Among Gay, Bisexual, and

Other Men Who Have Sex With Men in Singapore: Community-Based, Pragmatic, Randomized Controlled Trial. J Med Internet Res, 24(5), e31401. https://doi.org/10.2196/31401

Wall, H., Hansson, H., Zetterlind, U., Kvillemo, P., & Elgan, T. H. (2024). Effectiveness of a Web-Based Individual Coping and Alcohol Intervention Program for Children of Parents With Alcohol Use Problems: Randomized Controlled Trial. J Med Internet Res, 26, e52118. https://doi.org/10.2196/52118

Walton, M. A., Bohnert, K., Resko, S., Barry, K. L., Chermack, S. T., Zucker, R. A., Zimmerman, M. A., Booth, B. M., & Blow, F. C. (2013). Computer and therapist based brief interventions among cannabis-using adolescents presenting to primary care: one year outcomes. Drug Alcohol Depend, 132(3), 646-653. https://doi.org/10.1016/j.drugalcdep.2013.04.020

Walton, M. A., Chermack, S. T., Shope, J. T., Bingham, C. R., Zimmerman, M. A., Blow, F. C., & Cunningham, R. M. (2010). Effects of a brief intervention for reducing violence and alcohol misuse among adolescents: a randomized controlled trial. JAMA, 304(5), 527-535. https://doi.org/10.1001/jama.2010.1066

Walton, M. A., Resko, S., Barry, K. L., Chermack, S. T., Zucker, R. A., Zimmerman, M. A., Booth, B. M., & Blow, F. C. (2014). A randomized controlled trial testing the efficacy of a brief cannabis universal prevention program among adolescents in primary care. Addiction, 109(5), 786-797. https://doi.org/10.1111/add.12469

Whiteley, L. B., Brown, L. K., Curtis, V., Ryoo, H. J., & Beausoleil, N. (2018). Publicly Available Internet Content as a HIV/STI Prevention Intervention for Urban Youth. The Journal of Primary Prevention, 39(4), 361-370. https://doi.org/10.1007/s10935-018-0514-y

Ybarra, M., Goodenow, C., Rosario, M., Saewyc, E., & Prescott, T. (2021). An mHealth Intervention for Pregnancy Prevention for LGB Teens: An RCT. Pediatrics, 147(3). https://doi.org/10.1542/peds.2020-013607

Ybarra, M. L., Prescott, T. L., Phillips, G. L., II, Bull, S. S., Parsons, J. T., & Mustanski, B. (2017). Pilot RCT Results of an mHealth HIV Prevention Program for Sexual Minority Male Adolescents. Pediatrics, 140(1). https://doi.org/10.1542/peds.2016-2999

Reports of long-term outcomes to main trials included

Schinke, S. P., Fang, L., & Cole, K. C. (2009). Preventing substance use among adolescent girls: 1-year outcomes of a computerized, mother-daughter program. Addictive behaviors, 34(12), 1060–1064. https://doi.org/10.1016/j.addbeh.2009.06.007

Schwinn, T. M., & Schinke, S. P. (2010). Preventing alcohol use among late adolescent urban youth: 6-year results from a computer-based intervention. Journal of studies on alcohol and drugs, 71(4), 535–538. https://doi.org/10.15288/jsad.2010.71.535

Schinke, S. P., Schwinn, T. M., Di Noia, J., & Cole, K. C. (2004). Reducing the risks of alcohol use among urban youth: three-year effects of a computer-based intervention with and without parent involvement. Journal of studies on alcohol, 65(4), 443–449. https://doi.org/10.15288/jsa.2004.65.443

Schinke, S. P., Schwinn, T. M., & Fang, L. (2010). Longitudinal outcomes of an alcohol abuse prevention program for urban adolescents. The Journal of adolescent health : official publication of the Society for Adolescent Medicine, 46(5), 451–457. https://doi.org/10.1016/j.jadohealth.2009.11.208

Ybarra, M., Rosario, M., Saewyc, E., Goodenow, C., & Dunsiger, S. (2023). One-Year Follow-up After a Pregnancy Prevention Intervention for LGB+ Teens: An RCT. Pediatrics, 151(4), e2022059172. https://doi.org/10.1542/peds.2022-059172

Trial and participant characteristics

a) Sexual health interventions

Author (year)	Country	Participant age	Headline participant demographics	Focal equity relevant characteristic	Other equity relevant characteristics (>50% trial sample)
Bauermeister (2019)	USA	18 - 24<br>(M 21.67 SD 1.81)	100% assigned male at birth and identify as male (eligibility criterion).<br>Gay 89.0% (N=138); Bisexual 7.1% (N=11); Queer 3.9% (N=6)<br>White: 67.1% (N=104) Multiracial: 16.1% (N=25) Black: 10.0% (N=16) Asian: 5.2% (N=8) Middle Eastern: 0.6% (N=1) Native American: 0.6% (N=1) Hispanic/Latino: 30.0% (N=46)<br>Some high school 2.8%; High school/GED 10.6%; Technical/Associate 7.8%; Some college 40.0%; College graduate 29.4%; Graduate school 9.5%.<br>Mean number of male partners in past 30 days: 2.72 (SD 4.09).<br>Receptive anal sex: 61.9% (N=96); among these, condomless 69.7% (N=67); condomless with Sero discordant/unknown partner 37.3% (N=25). Insertive anal sex: 49.0% (N=79); among these, condomless 72.2% (N=57); condomless with Sero discordant/unknown partner 36.8% (N=21).	Personal characteristics (Sexual orientation)	Gender/sex (male)
Bauermeister (2024)	USA	15 - 24<br>(M 21.14, SD 2.09)	Non-Hispanic White: 45.6% (n = 130) Non-Hispanic Black: 18.2% Non-Hispanic Asian: 11.2% Latinx: 20.0% Multiracial: 4.2% Other: 0.7%<br>All participants were cisgender male<br>Gay 74%, Bisexual 20.47%, Other 5.3%<br>Socioeconomic status: food insecurity (16.7%) and residential instability (5.3%)	Personal characteristics (Sexual orientation)	Gender/sex (male)
Bennett (2015)	USA	18 - 26<br>(M 21, SD NR)	Hispanic 30 of 661 (4.5%); Asian 98 of 661 (14.8%); Non-Hispanic black 36 of 661 (5.5%); Non-Hispanic white 445 of 661 (67.3%); Other/multiracial 52 of 661 (7.9%).<br>Health insurance: UM SHIP or GradCare (University plans): 16.0% Parent s private insurance: 66.3% Medicaid: 1.5% Other insurance: 6.2% Full time: 8.3% Part time: 48.9% Unemployed: 42.4%<br>Ever had sexual intercourse 463 of 661 (70.1%); age at sexual debut median 18.0 years; ever had a Pap test 292 of 661 (44.2%); Pap test in past 12 months 205 of 661 (31.0%).	Gender/sex (female)	N/A
Biello (2021)	USA	15 - 24<br>(M 20.7, SD 2.4)	Latinx: 21%, Black, non-Latinx: 14%, White, non-Latinx: 51%, Multiracial/other: 14%<br>Less than high school educated: 16%, High school diploma/GED: 4%, some college/technical or vocational school: 53%, Four-year college graduate or more: 27%, currently in school: 70%	Personal characteristics (Sexual orientation)	Gender/sex (male)

			Insurance status: None: 17%, Public: 17%, Private: 65%<br>Cisgender men 100% (eligibility criterion).		
Biello (2022)	USA	15 - 24<br>(M 21.5, SD 2.1)	White, non-Hispanic: 58.3%, Hispanic/Latino: 15.0%, Asian, non-Hispanic: 11.7%, Black, non-Hispanic: 5.0%, Other/Multiple: 8.3%, Declined to answer: 1.7%<br>Currently employed: 62.1%<br>Current student: 63.8%<br>Low income: 36.2%, Middle income: 46.6%, High income: 10.3%<br>100% cisgender men.	Personal characteristics (Sexual orientation)	Gender/sex (male)
Biello (2025)	USA	15 - 29<br>(M 22.4, SD 3.17)	Race and ethnicity (total N=381): Asian, non-Hispanic 9% (n=34); Black, non-Hispanic 17.7% (n=67); Latino or Hispanic 17.7% (n=67); White, non-Hispanic 51.2% (n=194); Other or Mixed 4.5% (n=17).<br>Employment status (total N=381): Unemployed 31.9% (n=121); Employed part-time 33.3% (n=126); Employed full-time 34.8% (n=132).<br>Student status (total N=381): Currently enrolled 61.8% (n=235); Not currently enrolled 38.2% (n=145).<br>Insurance status (total N=381): Uninsured 11.3% (n=43); Own insurance 35.8% (n=136); Parent's insurance 51.6% (n=196); Do not know 1.3% (n=5).<br>All participants identified as cisgender men<br>Sexual orientation (total N=381): Bisexual 18.1% (n=69); Gay or homosexual 59.8% (n=228); Queer or same sex or other 20.2% (n=77); Heterosexual or straight 1.8% (n=7).<br>Condomless anal or vaginal sex in past 3 months 39.4% yes (n=150), 60.6% no (n=231).	Personal characteristics (Sexual orientation)	Gender/sex (male)
Bull (2012)	USA	16 - 25<br>(M 19.8, SD 2.1)	African American: 28.1% (Intervention) and 45.0% (Control)<br>Hispanic/Latino: 12.1% (Intervention) and 16.0% (Control) Asian: 27.5% (Intervention) and 7.4% (Control) White: 29.7% (Intervention) and 30.4% (Control) Oth American Indian/Alaskan Native 0.7% (intervention) and 0.8% (control), Pacific Islander 1.1% (intervention) and 0.5% control, Other Race 9.6% (intervention) and 12% (control)	Race/ethnicity/culture/ language (Latina and African American youth)	N/A
Carvaldho (2016)	Portugal	18 - 25<br>(M 19.8, SD NR)	100% male<br>Majority had 10-12 years of schooling (65%), - 33% had 9 years, ~2% had less than 9 years.<br>Mean number of sexual partners 5.88	Gender/sex (male)	N/A
Bull (2017)	USA	14 - 18<br>(M 14.9, SD 1.02)	Hispanic: 45.5% at baseline (both arms), Black 40.8% (TOP) and 40.5% (YAE!), White 32.2% (TOP) and 33.7% (YAE!), Other 41.2% (TOP) and 39.7% (YAE!)<br>Female: 53.2% (TOP) vs 50.9% (YAE!) at baseline; 47.3% vs 50.3% at 12 month follow up.	Race/ethnicity/culture/ language (Hispanic youth)	Gender/sex (female)

			Mother’s education (mean): 2.42 (TOP) vs 2.36 (YAE!) at baseline; 2.53 vs 2.34 at 12 months. Father s education (mean): 2.21 vs 2.04 at baseline; 2.20 vs 1.97 at 12 months.		
Chernick (2017)	USA	14 - 19 (M & SD, NR)	Hispanic: 83.7% (TM arm), 91.8% (SR arm)<br>79.6% in both arms had some type of insurance	Place of residence (Youth accessing care from an urban emergency department)	Race/ethnicity/culture/ language
Chernick (2022)	USA	14 - 19 (M 17.74, SD 1.27)	Hispanic: 62 (87.3%) intervention and 63 (86.3%) control, African American: 20 (28.6%) intervention, 14 (19.4%) control; Native Hawaiian or Pacific Islander Intervention 0 (0) Control 3 (4.2%); White 2 (2.8%) 4 (5.6%); Other 39 (53.9%) 40 (55.6%)<br>Female only (all participants were adolescent females)<br>Population described as publicly insured and of low socioeconomic status	Place of residence (Youth accessing care from an urban emergency department)	Race/ethnicity/culture/ language<br>Gender/sex (female)
Chernick (2025)	USA	14 - 21 (M 17.9, SD NR)	Hispanic or Latino: 77.3% (92/119) American Indian/Alaskan Native 2.5% (3/119); Asian 0.8% (1/119); Black or African American 30.3% (36/119); Native Hawaiian/Pacific Islander 4.2% (5/119); White 7.6% (9/119); More than one race 29.4% (35/119); Unsure/prefer not to answer 31.1% (37/119)<br><br>Male (sex at birth)100% (119/119)<br><br>Straight/heterosexual 97.5% (116/119); Bisexual/questioning/other 4.2% (5/119); Transgender/gender nonconforming 1.7% (2/119)	Place of residence (Youth accessing care from an urban emergency department)	Race/ethnicity/culture/ language<br>Gender/sex (male)
Christensen (2013)	USA	18 - 24 (M 21.3, SD 1.7)	White/Caucasian: 71.1% (n = 349) in control; 76.1% (n = 338) in treatment, Latino/Hispanic: 15.5% (n = 76) in control; 12.4% (n = 55) in treatment Black/African American: 13.4% (n = 66) in control; 11.5% (n = 51) in treatment<br><br>All participants were male (men who have sex with men).<br><br>Postsecondary education: 80.2% (n = 394) in control; 83.6% (n = 371) in treatment<br><br>Gay/homosexual: 36.6% (n=376) in control; 74.5% (n=74.5%) in treatment, Bisexual: 11.4% (n=56) in control; 14.7% (n=65) in treatment, Other: 11.6% (n=57) in control; 10.4% (n=46) in treatment	Personal characteristics (sexual orientation)	Gender/sex (male)
Cordova (2020)	USA	13 - 21 (M 18.82, SD 2.1)	46% non-Hispanic White, 42% Black 2% Native American 8% more than one race 2% Other<br><br>82% female, 8% male 8% trans male, 1 refused to respond	Place of residence (Youth accessing care from Community Health Clinics)	Gender/sex (female)

			36% completed some college, 30% completed high school 34% completed grades 7 11		
Schnall (2022)	USA	13 - 18 (M 16.2, SD 1.4)	Hispanic or Latino: 40.9%, not Hispanic or Latino: 59.1%<br><br>Some high school: 71.3% High school diploma/GED: 12.2% Some college: 6.6%<br><br>Male: 97%, Nonbinary: 1.7%, Genderqueer: 0.8%, Other 0.5%<br><br>Only gay or homosexual: 53.5%, mostly gay or homosexual 21.9%, Bisexual 21.2%, mostly heterosexual: 0.5%, only homosexual 0.3%. other 2.6%	Personal characteristics (sexual orientation)	Gender/sex (male)
Fiellin (2017)	USA	11 - 14 (M 12.9, SD 1.1)	Hispanic: 172 participants (55.5%), Non-Hispanic: 138 participants (44.5%)<br><br>Male: 177 participants (53.2%), Female: 156 participants (46.8%)	Race/ethnicity/culture/ language (Black/African American and Hispanic youth)	N/A
Gerend (2021)	USA	18 - 25 (M 22.78, SD 2.03)	White: 54%, Black or African American: 21% Asian: 7% Multiracial: 5% Latino ethnicity: 32%<br><br>Some high school/high school degree/GED: 32% (intervention), 26% (control)Some college or trade school certificate: 37% vs 39% College degree: 21% vs 21% Some graduate school/graduate degree: 10% vs 15%<br><br>All participants were assigned male at birth and identified as male.<br><br>Gay: 74%, Bisexual: 22%, Other (e.g., queer, pansexual) 4%	Personal characteristics (sexual orientation)	Gender/sex (male)
Hebert (2018)	USA	15 - 29 (M 22, SD NR)	African American (76.3%), Latina/Hispanic (21.7%), Other (1.9%)<br><br>100% Female (young women)<br><br>High school graduate or less: 33.8%, Some college: 52.9% College degree or higher: 13.2%<br><br>Cohabiting with partner or spouse: 19.0% In a relationship, not cohabiting: 34.2% Dating/casual partners: 22.0% Single: 24.9%	Race/ethnicity/culture/ language (African American and Latina youth)	Gender/sex (female)
Hightow-Weidman (2019)	USA	18 - 30 (M 24.33, SD 3.22)	All participants self-identified as Black (n = 474 total)<br>High school educated: 43 (9.1%). High school/GED/some technical/college: 316 (66.7%). College degree or more: 115 (24.2%).<br><br>All participants were biologically male.	Personal characteristics (sexual orientation)	Race/ethnicity/culture/ language<br>Gender/sex (male)

			Gay 316 (66.7%), Bisexual 95 (20.0%), Other 63 (13.3%). HIV positive 199 (42%), HIV negative 275 (58%)<br>Homeless (last 6 months): 104 (21.9%); Arrested (last 3 months): 28 (5.9%).		
Jones (2013)	USA	18 - 29<br>(M 22,0, SD NR)	African American/Black: 210/238 (88.2%), Latina or Caribbean: 8.0%<br>Unemployed: 147/238 (61.8%)<br>Female: 238/238 (100%)<br>11th grade education or less: 26.0%, 12th grade education: 86/238 (36.1%) One or two years of college: 78/238 (32.8%)<br>Any male partner had sex with other women: 237/238 (99.6%) Any male partner had sex with men: 85/238 (35.7%) Any male partner injected drugs: 68/238 (28.6%) Any unprotected vaginal sex with a high risk partner (past 3 months): 236/238 (99.2%) Any unprotected anal sex with a high risk partner (past 3 months): 105/238 (44.1%)	Race/ethnicity/culture/ language (African American youth)	Gender/sex (female)
Kissinger (2023)	USA	18 - 29	All participants identified as Black/African American, with 3.6% also reporting a second race/ethnicity (1.7% Hispanic, 1.3% American Indian, 0.2% Asian, 1.1% other non-specified).<br><br>All participants were female.<br><br>37.4% employed; 18 participants worked full time (all in control arm).<br><br>98.8% completed high school or equivalent; 97.6% were in school at baseline.	Race/ethnicity/culture/ language (Black/African American youth)	Gender/sex (female)
Mustanksi (2013)	USA	18 - 24<br>(M 21.362, SD 1.97)	White–Latino 46.0% vs 46.2%; White–Non-Latino 24.0% vs 26.9%; African American 14.0% vs 11.5%; Other 16.0% vs 15.4%.<br><br>Employed 56.0% (KIU!) vs 73.1% (control); currently a student 46.9% vs 59.6%.<br><br>Some high school or graduate 24.0% (KIU!) vs 21.2% (control); some college or graduate 76.0% vs 78.8%.<br><br>All participants were assigned male at birth and identified as male<br><br>Gay/homosexual 78.0% (KIU!) vs 86.5% (control); bisexual/other 22.0% vs 13.5%.	Personal characteristics (sexual orientation)	Race/ethnicity/culture/ language<br><br>Gender/sex (male)
Mustanski (2018)	USA	18 - 29<br>(M & SD NR)	White 330/901 (36.6%); Black 219/901 (24.3%); Hispanic/Latino 260/901 (28.9%); Other 92/901 (10.2%).<br><br>High school or less 113/901 (12.5%); Some college 252/901 (27.9%); College 418/901 (46.4%); Graduate degree 118/901 (13.1%).	Personal characteristics (sexual orientation)	Race/ethnicity/culture/ language<br><br>Gender/sex (male)

			Eligibility required being assigned male at birth with current male gender identity;<br><br>Sexual orientation Gay 777/901 (86.2%), Bisexual 104/901 (11.5%), Straight/Other 20/901 (2.2%).		
Manlove (2020)	USA	18 - 20<br>(M 18.8, SD NR)	76% Black or Latinx<br><br>100% female (self-identified)<br><br>>70% had completed some college, technical school, or more	Race/ethnicity/culture/ language (Black and Latinx youth)	Gender/sex (female)
Martinez-Garcia (2023)	USA	14 - 18<br>(M & SD NR)	Hispanic: 20.6%, Non-Hispanic White: 52.1% Non-Hispanic Black: 10.9% Non-Hispanic Other: 10.8%<br><br>Female adolescents only (100%)<br><br>Mother s education: High school graduate or less: 22.6% Some college: 18.4% College graduate: 52.1%<br><br>Heterosexual: 62.0% LGBTQ (combined): 32.1%	Gender/sex (female)	N/A
Mustanski (2025)	USA	13 - 18	Hispanic/Latinx: 33.7% (ER1), 40.1% (ER2), 33.5% (ER3), 38.3% (ER4); Total: 36.4%, Non-Hispanic/Latinx: 66.3% (ER1), 59.9% (ER2), 66.5% (ER3), 61.7% (ER4); Total: 63.6%<br><br>Male: 94.2% Female: 0.9% Gender nonconforming: 1.0% Genderqueer: 0.8% Nonbinary: 1.9% Transgender: 0.6% Other: 0.6% Gay: 67.6% Bisexual: 24.4% Pansexual: 3.6% Queer: 1.5% Unsure/questioning: 2.4% Lesbian: 0.2% Asexual: 0.1% Other: 0.3%<br><br>Language preference: English: 96.2% Spanish: 3.8%	Personal characteristics (sexual orientation)	Gender/sex (male)
Patel (2024)	USA	13 - 24<br>(M 17, SD 4)	Control arm: Black 68%, White 4%, Hispanic 22%, Asian 2%, Other 5%. Intervention arm: Black 67%, White 3%, Hispanic 28%, Asian 0%, Other 2%<br><br>Female 100% (males were excluded)<br><br>Described as low socioeconomic backgrounds among patients	Gender/sex (female)	Race/ethnicity/culture/ language
Reback (2024)	USA	16 - 24<br>(M & SD, NR)	African American/Black 10.2%, Latine 7.1% White 57.9% Others 24.8%<br><br>Gender identity at baseline: nonbinary AFAB 44.5%, Nonbinary AMAB 13.0% Trans man/masculine 34.6% Trans woman/feminine 7.9%	Gender/sex (transgender and gender expansive youth)	N/A

			High school/GED or less 22.8%, Currently enrolled in college or graduate school 51.6% Completed some college or higher 25.2% Decline to answer 0.4%<br><br>Income in the past month $0 $999 53.5%, $1,000 $4,999 38.2% $5,000+ 2.4% Don t know 3.9% Decline to answer 2.0%		
Santa (2021)	USA	18 - 25 (M 21.2, SD 2.1)	White or Caucasian: 3.1%, Black or African American: 57.7% Hispanic or Latino: 9.3% American Indian/Asian or Pacific Islander/other: 14.4% Multiracial: 15.5%<br><br>Male: 57.7%, Female: 35.1%, Transgender/genderqueer/other/missing: 7.2%<br><br>Sexual orientation: Gay 8.2%; Lesbian 6.2%; Straight 52.6%; Bisexual 23.7%; Asexual/pansexual/other 9.3% Involved with the juvenile justice system: 40.2%<br><br>Experiencing homelessness: 100%	Place of residence (homeless youth)	Race/ethnicity/culture/ language
Schnall (2025)	USA	18 - 29 (M 24.1, SD 3.28)	Hispanic/Latino: 160 (30.6%), Not Hispanic/Latino: 361 (69.0%)<br><br>Male: 475 (90.9%), Trans feminine: 13 (2.5%) Genderqueer: 9 (1.7%) Nonbinary: 23 (4.4%) Other: 3 (0.6%)<br><br>Did not finish high school: 40 (7.6%), Graduated high school/GED: 77 (14.7%) Some college/technical training: 125 (23.9%) Graduated from a 4year college: 135 (25.8%) Advanced graduate degree: 136 (26.0%) Does not apply: 7 (1.3%)<br><br>Sexual orientation: Bisexual 72 (13.8%); Gay/homosexual 380 (72.7%); Queer 57 (10.9%); Straight/heterosexual 5 (1.0%); Other 9 (1.7%)	Personal characteristics (sexual orientation)	Gender/sex (male)
Starosta (2016)	USA	College students (M 19.02, SD NR)	Caucasian 59%, African American 19%, Hispanic or Latino 14.9%, Asian 7.6%, Other 5.5%.<br><br>100% college students.<br><br>100% female;<br><br>100% heterosexual.	Gender/sex (female)	N/A
Stephenson (2020)	UK	15 - 30 (M 24, SD NR)	White 67.0% (n = 306) intervention; 71.0% (n = 326) control, Mixed 11.6% (n = 53); 10.5% (n = 48) Asian 10.3% (n = 47); 8.1% (n = 37) Black 9.0% (n = 41); 8.3% (n = 38) Other 2.2% (n = 10); 2.2% (n = 10)	Gender/sex (female)	N/A

			Female (all participants were women aged 15 30 years)<br><br>Degree 51.3% (n = 238) intervention; 49.9% (n = 231) control, Diploma in higher education 10.6%; 9.7% A/AS levels 21.3%; 23.5% O levels/GCSE 9.7%; 7.8% Other 5.4%; 5.8% None 1.7%; 3.2%<br><br>Language (first language): English 80.8% (n = 375) intervention; 84.2% (n = 390) control Not English 19.2% (n = 89); 15.8% (n = 73		
Tan (2022)	Singapore	18 - 29 (M 24.0, SD 2.98)	Chinese: Intervention 75.3% (113/150); Control 82.7% (124/150) Educational attainment below college: Intervention 61.3% (92/150); Control 63.3% (95/150)<br><br>Monthly income below SGD 5000: Intervention 48.7% (73/150); Control 54.7% (82/150)<br><br>All participants were of male gender (self-reported).<br><br>Sexual orientation: Gay Intervention 77.3% (116/150); Control 84.7% (127/150)<br><br>Housing type (proxy for socioeconomic status): Public housing Intervention 84.0% (126/150); Control 74.7% (112/150)	Personal characteristics (sexual orientation)	Gender/sex (male)
Whiteley (2018)	USA	15 - 24 (M 18.6, SD NR)	Black/African American 52% total; Intervention 50%; Control 54%., Hispanic 36% total; Intervention 37%; Control 36%.<br><br>Male 62% total; Intervention 65%; Control 59%.<br><br>Sexual orientation: Nonheterosexuality orientation 47% total; Intervention 45%; Control 48%.	Race/ethnicity/culture/ language (Black/African American and Hispanic participants)	Gender/sex (male)
Ybarra (2017)	USA	14 - 18	White: 69.3% (intervention) 65.1% (control); African American: 16.8% (intervention) 13% (control); Other races: 13.9% (intervention) 21.9% (control) Hispanic ethnicity: 20.4% (intervention) 24.7% (control)<br><br>Cisgender males only<br><br>Low income: 20.4% (intervention) 27.4% (control); Middle income: 59.1% (intervention) 48.0% (control) High income: 20.4% (intervention) 24.7% (control)<br><br>Gay: 74.5% (intervention) 69.2% (control), remainder bisexual	Personal characteristics (sexual orientation)	Gender/sex (male)
Ybarra (2021)	USA	14 - 18	24% Hispanic ethnicity; majority non-Hispanic.<br><br>cisgender girls 99%	Personal characteristics (sexual orientation)	Gender/sex (female)

			Self-appraised household income: Lower than average: 27 30%<br><br>Similar to average: 51 52% Higher than average: 15 16%<br><br>Lesbian (43 45%), Bisexual (41 43%), Pansexual (25 30%), others (queer, questioning, etc.).		
Kauffman (2025)	USA	16 - 20 (M 18.02, SD 1.39)	American Indian and Alaska Native (AIAN) Intervention: 49.5% AIAN only, 50.5% AIAN multi race Comparison: 43.0% AIAN only, 57.0% AIAN multi race<br><br>Grade 11 or less: 32.1% (comparison), 37.2% (intervention), Grade 12 or GED: 32.1% (comparison), 35.3% (intervention) Some college/college grad: 35.7% (comparison), 27.5% (intervention)<br><br>Receive government assistance: 46.2% (comparison), 51.2% (intervention), Economic problems past month: 70.3% (comparison), 63.8% (intervention)<br><br>Female: 86.0% (comparison), 89.0% (intervention) Gender queer/nonbinary: 8.1% (comparison), 3.7% (intervention) Two spirit: 4.1% (comparison), 5.5% (intervention) Other/not sure: 1.8% (both)	Race/ethnicity/culture/ language (American Indian and Alaskan Native youth)	Gender/sex (female)
Reiter (2023)	USA	18 - 25	No Hispanic White = 47%; No Hispanic Black = 11%; Hispanic = 29%; No Hispanic Other = 14%<br><br>High school or less = 31%; Some college or more = 69%<br><br>All participants were cisgender male (100%)	Personal characteristics (sexual orientation)	Race/ethnicity/culture/ language<br>Gender/sex (male)

M = mean, SD = standard deviation, NR = not reported

b) Substance use interventions

**Author (year)**	**Country**	**Participant age**	**Headline participant demographics**	**Focal equity relevant characteristic**	**Other equity relevant characteristics (>50% trial sample)**
Fang (2010)	USA	10 - 14 (M & SD NR)	Asian-American adolescent girls<br>Mothers' highest education at baseline): High school 32.0% (control) and 22.2% (intervention), college 40.0% (control) and 24.6% (intervention), graduate school 28.0% (control) and 35.2% (intervention)<br>Participants were largely from socioeconomically advantaged backgrounds<br>100% Female adolescent girls.	Race/ethnicity/culture/ language (Asian American youth)	Gender/sex (female)

			Single-parent households 18.0% (control) and 13.0% (intervention).		
Mason (2015)	USA	14 - 18 (M 164.4, SD 1.38)	90.8% black or African American; 5.3% white; 3.9% unknown or other.<br>56.6% male.<br>68.4% reported living with a current cigarette smoker in the household.	Race/ethnicity/culture/ language (African American youth)	Gender/sex (male)<br>Features of relationships (living with cigarette smoker)
Mason (2016)	USA	14 - 18 (M 16.2, SD 1.39)	90.5% Black or African American, 6.5% White, 3% Other/Unknown<br>52.5% female<br>74.2% reported living with a current cigarette smoker	Race/ethnicity/culture/ language (African American youth)	Gender/sex (female)<br>Features of relationships (living with cigarette smoker)
Prokhorov (2008)	USA	High school students	Hispanic: 58.9% in intervention group vs. 41.6% in comparison group (p < .01).<br>Schools located in socioeconomically disadvantaged communities (qualitative description).<br>Peer smoking: Initiators had 73% close friends who smoked vs. 42% among non-initiators (p < .05). Mother smokes: 8.2% initiation in comparison group vs. 0% in intervention group (p < .01). Father smokes: 9.1% initiation in comparison group vs. 2.0% in intervention group (p < .01).	Place of residence (Inner-city schools serving predominantly low SES, minoritized ethnic participants)	
Schinke (2005)	USA	9 - 13 (M & SD NR)	54% Black, 30% Hispanic, 11% White, 5% Asian or other.<br>Agencies served economically disadvantaged youths; household incomes at or below the US federal poverty guideline for a family of five.<br>51.4% female.<br>Divorced or separated households: 46.3% in intervention families and 35.0% in control families.<br>Language preference noted among parents of Hispanic youths; 11.5% preferred Spanish for parental consent and materials.	Place of residence (Afterschool agencies serving urban, economically disadvantaged areas)	Race/ethnicity/culture/ language
Schinke (2009)	USA	Range NR (M 12.76, SD 1.0)	Black 40.6%; White 23.2%; Latina 23.1%; Asian 10.8%; Other 1.7%.<br>Mothers' education: below high school 6.3%; high school 9.1%; some college 28.3%; associate or bachelor's degree 42.6%; graduate degree 13.7%. [44. Schinke 2009 | PDF]<br>100% Adolescent girls.<br>Single-parent 43.7%; two-parent 56.3%.	Gender/sex (female)	Race/ethnicity/culture/ language
Schwartz (2014)	Canada	13 - 15 (M & SD NR)	Aboriginal: 49/605 (8.1%), Non-Aboriginal: 558/608 (91.8%)	Gender/sex (female)	N/A

			All participants were female (nonsmoking adolescent girls).<br>Family income: Below average: 31/574 (5.4%) Average: 463/579 (80.0%) Above average: 85/578 (14.7%)<br>Parent(s) smoke(s): 141/562 (25.1%) Friends smoke: 84/488 (17.2%) Daily or almost daily smoking in home: Every day: 21/600 (3.5%) Almost every day: 65/607 (10.7%)		
Schwinn (2010)	USA & Canada	13 - 14 (M & SD NR)	White 60.6%, Black 16.5%, Latina 7.2%, Asian 6.8%, Other 8.9%.<br>Female only<br>Living arrangement at baseline: Mother and father 56.8%, Two-parent step family 11.9%, Single parent 25.4%, Grandparents 3.4%, Foster or other	Gender/sex (female)	N/A
Schwinn (2015)	USA	Range NR (M 16.08, SD 0.58)	White 62.1%, Hispanic 13.2%, Black 9.6%, Asian 7.4%, Other 7.5%<br>Male 32.7%, Female 50.9%, Queer/fluid/other 16.4%<br>Sexual attractions: Same sex 38.7%, Both sexes 49.3%, Opposite sex 6.2%, Not sure 5.8%.	Personal characteristics (sexual orientation)	Gender/sex (female)
Schwinn (2021)	USA	12 - 15 (M 14.1, SD NR)	All participants identified as Hispanic.<br>Female: 60% of participants.<br>2 years of college: 71.1% (control); 63.4% (intervention)<br>Language spoken at home: Spanish only or mostly Spanish: 33.3% (control); 35.4% (intervention) Spanish and English equally: 27.6% (control); 30.1% (intervention) English only or mostly English: 39.0% (control); 34.5% (intervention)	Race/ethnicity/culture/ language (Hispanic youth)	Gender/sex (female)
Wall (2024)	Sweden	15 - 19 (M 17.0, SD 1.23)	Female 89.3% (control), 86.1% (intervention)<br>Mother: Employed 78.6% (control), 84.2% (intervention)<br>Father: Employed 69.9% (control), 71.3% (intervention<br>Family member with perceived alcohol problems: Father 69.9% (control), 74.3% (intervention) Mother 34% (control), 27.7% (intervention)<br>Living with both parents: 28.2% (control), 28.7% (intervention) With one parent: 24.3% (control), 19.8% (intervention) Part time with mother and father: 12.6% (control), 12.9% (intervention) Not living with any parent: 8.7% (control), 15.8% (intervention)	Features of relationships (Youth with parents with alcohol use problems)	Gender/sex (female)

Walton (2013)	USA	12 - 18 (M 16.3, SD 1.6)	African American: 60.7%, Hispanic ethnicity: 11.0%<br>Male: 33.5%<br>Failing grades: 25.9%, Dropped out of school: 5.8%	Place of residence (Youth accessing support from federally qualified community health clinics in medically underserved areas)	Gender/sex (female)
Walton (2014)	USA	12 - 18 (M 14.9, SD 1.9)	African American: 63.7%, Hispanic ethnicity: 9.2%<br>Male: 43.0%<br>Failing grades: 17.1%	Place of residence (Youth accessing support from federally qualified community health clinics in medically underserved areas)	Gender/sex (female)
Walton (2010)	USA	14 - 18 (M 16.8, SD 1.3)	African American 55.9%, White 39.1%, Other 5.0%; Hispanic ethnicity 6.5%.<br>Male 43.5%.<br>Failing grades (some Ds/Fs): 44.1%; Dropped out of school: 10.1%.<br>Family receipt of public assistance: 57.4%.<br>Gang involvement 7.3%.<br>Discharged from emergency department on day of recruitment 93.0%.	Place of residence (Youth accessing support from federally qualified community health clinics in medically underserved areas)	Race/ethnicity/culture/language<br>Gender/sex (female)
Gilmore (2023)	USA	14 - 18 (M 15.63, SD 1.31)	Black or African American: 117/123 (95.1%), Asian: 1/123 (0.8%) White: 3/123 (2.4%) Not listed/prefer not to answer: 2/123 (1.6%)<br>Boy: 58/123 (47.2%), Girl: 62/123 (50.4%) Nonbinary: 3/123 (2.4%)	Place of residence (Youth accessing support from Medicaid-serving paediatric primary care clinics)	Race/ethnicity/culture/language

M = mean, SD = standard deviation, NR = not reported

c) Mental health interventions

**Author (year)**	**Country**	**Participant age**	**Headline participant demographics**	**Focal equity relevant characteristic**	**Other equity relevant characteristics**
Aboody (2020)	Israel	19 - 28 (M 23.52, SD 1.45)	100% female<br>100% heterosexual<br>Hebrew main spoke language	Gender/sex (female)	N/A
Ampatzoglou (2024)	Spain	11-14 (M 2.57, SD 0.39)	100% female	Gender/sex (female)	N/A
Bauermeister (2022)	USA	13 - 19 (M 16.5, SD 1.5)	77.8% identified as racial or ethnic minorities (Black, Hispanic/Latinx, Asian, multiracial, etc.)<br>mostly middle/high school students (6th 11th grade).<br>subjective SES reported (upper middle, middle, working class, low income).<br>Sex at birth reported (77.4% female, 22.6% male)	Personal characteristics (sexual orientation)	Race/ethnicity/culture/ language<br>Gender/sex (female)

			Sexual and gender minority status.		
Egan (2021)	USA	14 - 18	White: 62.1% Latinx: 20.8% Asian or Pacific Islander: 3.8% Black: 3.3% Multiracial: 10.0%<br>Parents highest education level: Did not finish high school: 10.0% Graduated high school: 15.8% Attended college but did not complete: 17.9% Graduated from college: 54.2% Don t know: 2.1%<br>Free or reduced priced lunch: Yes: 36.7% No: 50.0% Unsure: 13.3%<br>Cisgender girl: 16.3% Cisgender boy: 36.7% Gender minority: 47.1%<br>Sexual and gender minority identity (eligibility criterion).	Personal characteristics (sexual orientation)	N/A
Glass (2022)	USA	18 - 24	Latina: 14.53% (control), 12.50% (intervention)<br>Participants were women (including cisgender and transgender women).<br>Year in school: Freshman: 24.39% (control), 17.61% (intervention) Sophomore: 23.16%, 25.00% Junior: 20.90%, 25.00% Senior: 24.86%, 27.84% Graduate student: 6.78%, 4.55%<br>Partner gender male: 92.70% (control), 90.91% (intervention)<br>Relationship status: Ex partner: 71.91% (control), 65.14% (intervention)	Gender/sex (female)	Features of relationships (75.9% of youth experienced intimate partner violence)
Glass (2024)	USA	15 - 17	Latino or Hispanic 21.8%. Race: White 60.9%, Black or African American 28.6%, Asian 8.4%, American Indian or Alaskan Native 4.3%, Native Hawaiian or Pacific Islander 1.5%, Other races 9.2% (multiple selections permitted). .<br>Education: Not in school 5.1%, Freshman 10.7%, Sophomore 19.0%, Junior 36.9%, Senior 28.2%.<br>Gender identity. Female 63.9%, Male 16.7%, Trans male or female 4.9%, Gender diverse 10.2%, Something else or not sure 4.3%.<br>At baseline, 64.7% were no longer in a relationship with the abusive partner; 23.0% were in a boyfriend, girlfriend, or partner relationship; 6.2% in the “talking” phase; 6.1% in a casual or nonexclusive relationship.	Features of relationships (Youth who had experienced domestic violence victimisation in the last 6 months)	Gender/sex female)
Rushing (2021)	USA	15 - 24	American Indian and Alaska Native (AI/AN) 100%<br>Female: 66.3% (552/833) , Male: 28.9% (241/833)<br>Gender fluid: 1.1% (9/833) Two spirit: 2% (14/833) Other: 0.7% (6/833)<br>Most planned to go to college: 52.8% (440/833); some undecided: 10.6% (88/833).	Race/ethnicity/culture/language (American Indian and Alaskan Native youth)	Gender/sex (female)

			Straight or heterosexual: 75.8% (628/833) Bisexual: 9.4% (78/833) Lesbian/gay/homosexual: 5.1% (42/833) Pansexual: 2.9% (24/833) Others (including asexual, queer, two spirit): smaller percentages.		
Jeong (2024)	South Korea	(M 21.6 SD 3.4)	Education; all university students.<br>Socioeconomic status (perceived economic status, total sample): Very low 7.1% Low 35.7% Moderate 45.2% High 10.7% Very high 1.2% Sex: female 100%.<br>Marital status unmarried 100%.	Gender/sex (female)	N/A
Shen (2022)	USA	(M 15.06, SD 0.97)	White/Caucasian: 48.14%, Multiracial: 20.45% Hispanic/Latinx: 12.08% Black/African American: 8.92% Asian: 7.43% American Indian or Alaska Native: 1.12% Native Hawaiian or Other Pacific Islander: 0.56% Other: 0.74% Prefer not to say: 0.56%<br>Assigned female at birth: 89.22%, Assigned male at birth: 10%<br>Gender identity diversity:<br>Woman/Girl: 36.62% Nonbinary: 30.86% Transgender identities and other gender diverse categories also reported. Female to male transgender: 11.83%; Male to female transgender: 1.91%; Trans male/Transmasculine: 17.18%; Trans female/Trans feminine: 3.05%; Genderqueer: 11.07%; Gender expansive: 3.44%; Androgynous: 11.07%; Nonbinary: 30.92%; Two-Spirited: 0.76%; Third Gender: 0.0%; Agender: 4.58%; Other: 14.12%; Not Sure: 9.54%<br>Sexual orientation diversity: Bisexual: 24.35% Gay/Lesbian/Homosexual: 24.16% Pansexual: 16.73% Queer: 13.57% Asexual: 7.06% Other: 4.83% Unsure/Questioning: 9.11%	Personal characteristics (sexual orientation)	Gender/sex

M = mean, SD = standard deviation, NR = not reported

Intervention details

a) Sexual health interventions

**Author (year)**	**Mode of delivery**	**Theoretical model**	**Intervention**	**Control**	**Background support to both groups**
Bauermeister (2019)	Website	Cognitive affective dual process decision making	myDEx, a tailored online HIV prevention intervention for single young men who have sex with men, structured into six sessions that cover	Attention control information only site that mirrored the CDC HIV Risk Reduction Tool.	Online delivery with six sessions available on a study website; participants in both arms had access for 90 days

		framework	sexuality and relationships, desires and behaviours, comprehensive sex education, sexual wellbeing, sexual communication, and health resources. Each session includes a core message, in depth content, and interactive activities such as roleplays, quizzes, and a diary to reflect on partner seeking behaviours. Access to Session 1 was required before the remaining sessions and features could be used.	Content was divided into six sessions to match the myDEX structure, ensuring all participants received HIV prevention information while avoiding confounding due to non-HIV content	and completed follow-up assessments at 30, 60, and 90 days.<br><br>Incentives were provided at each assessment to promote retention, and both arms delivered HIV prevention content appropriate for the study population.
Bauermeister (2024)	Website	No specific behavioural theory named	GCPLUS (full, individually and systems tailored)<br><br>Individually tailored educational content based on participant baseline and follow up psychosocial indicators, and systems level tailoring ranked sites using mystery shopping data and participant feedback.	GCTLO (test locator only). Participants received the testing site locator without the tailored educational content.	Access to an HIV or STI testing site locator.<br><br>Follow-ups at 1, 3, 6, 9 and 12 months with e-gift card incentives, and participants had access to the WebApp for the full 12 months.
Bennett (2015)	Website	Based on tailored health communication principles,	MeFirst – an individually tailored online educational website configured based on participants baseline survey responses. It included seven topic pages with factual information on HPV and the vaccine, tailored to demographics, attitudes, and perceived barriers<br>Individually tailored website with up to 160 personalized elements, including name, doctors name, racial background images, and content addressing endorsed barriers.	A single webpage containing the CDC Vaccine Information Statement for the quadrivalent HPV vaccine.	Educational content about HPV and vaccination, aiming to inform participants about risks, benefits, and vaccine safety.
Biello (2021)	Smart phone application	LYNX was developed using the Information–Motivation–Behavioural Skills (IMB) model and principles of gamification	Participants randomised to receive either the LYNX or MyChoices mobile app, which included the ability to order HIV self-test kits and STI self-collection kits, plus broader sexual health content (PrEP information, resources, etc.). Intervention exposure lasted for the 6-month follow-up period (participants	Standard of care (CDC pamphlets with information on HIV testing and PrEP). Exposure also lasted for the 6-month follow-up period.	All participants received screening, informed consent/assent, and study assessments. All arms were supported by study staff, IRB oversight, and confidentiality protections.

		MyChoices was developed using the Social Cognitive Theory (SCT) and the Health Belief Model (HBM)	completed assessments at baseline, 3 months, and 6 months).		Compensation was provided for assessments (baseline and follow-up).
Biello (2022)	Smartphone application	Social Cognitive Theory	MyChoices mobile app intervention, adapted from an app previously shown to increase HIV testing and PrEP uptake among adult MSM. It included features such as tailored HIV testing plans, sexual health information, free home HIV/STI testing kits, condoms, lubricants, geolocation-based notifications, and maps of local HIV/STI testing sites and PrEP providers. Participants had access to the app throughout the 6-month follow-up period.	Standard of care<br>Participants received CDC fact sheets on HIV testing and PrEP uptake and had access to any services available at each study site. Standard of Care was remained available throughout the 6-month follow-up period.	CDC fact sheets on HIV testing and PrEP uptake and had access to any services available as standard of care at each study site.
Biello (2025)	Smart phone application	MyChoices: social cognitive theory.<br><br>LYNX: information–motivation–behavioural skills model	MyChoices - Primary components included: sexual health information (infographics, videos, links), access to free home HIV and STI testing kits and condoms and lubricant, tailored HIV prevention recommendations and testing plans via monthly check-ins, reminders for HIV testing including geolocation-based notifications, and GPS-enabled maps of local HIV or STI testing locations and PrEP providers. Study staff supported downloading and provided a brief, structured demonstration.<br><br>LYNX - Mobile app guided by the information–motivation–behavioural skills model. Primary components included: personalised Sex Pro score, sexual diary to track partners and encounters, testing reminders, ability to order free home HIV and STI testing	Receipt of CDC fact sheets on HIV testing and PrEP and access to any services available as standard of care at each site. No study app was provided.	All participants received CDC fact sheets on HIV testing and PrEP and had access to services available as standard of care at each study site.<br><br>Assessments were self-administered via a secure web system.<br><br>Smartphone ownership or regular access was an eligibility criterion for all arms.<br><br>Access to all apps and materials until 6-month endpoint.

			kits and condoms and lubricant, geolocation map of testing sites and PrEP clinics, PrEP information and navigation roadmap, trends and insights with infographics of partner types and sexual activities, and a two-way chat feature for real-time PrEP navigation assistance. Study staff supported downloading and provided a brief, structured demonstration.		
Bull (2012)	Social media	The intervention content was based on two fundamental ideas -<br>Sexual health is a human right and a function of social justice, and youth need a space to share ideas and concepts with peers and professional experts.	Just/Us, a Facebook page developed with youth input, featuring sexual health topics, videos, quizzes, games, and discussions. It featured eight broad sexual health topics (for e.g., communication about sexual history, healthy relationships, condom negotiation skills, and STI testing). Content was delivered through daily updates including videos, quizzes, games, and threaded discussions led by youth facilitators.	1824 News, a Facebook page with current events and no sexual health content.	Facebook content for two months and were incentivised for participation and follow up assessments.<br><br>Recruitment and eligibility criteria were identical, including liking the assigned Facebook page to receive content in their newsfeed.
Bull (2017)	Text messaging, website	Integrated Theory of mHealth, which combines psychosocial messaging related to antecedents of behaviour change (for e.g., attitudes, norms, self-efficacy).<br>Strategic health communication approaches.	Teen Outreach Program (TOP) plus the Youth All Engaged! (YAE!) text messaging programme.<br><br>YAE! delivered five to seven automated text messages weekly, with 40% being bidirectional (requiring a response). Messages were designed using the Integrated Theory of mHealth, focusing on attitudes, norms, self-efficacy, and cues to action. Content included polls, myth/fact questions, reminders, and motivational quotes to reinforce TOP content.	Teen Outreach Program (TOP) alone, without any text messaging component.	25 weekly TOP sessions over 9 months and completed 20 hours of community service learning.

			Teen Outreach Program (TOP), an evidence based teen pregnancy prevention curriculum focused on positive youth development, service learning and comprehensive sexual health education.		
Carvalho (2016)	Computer software	Health Action Process Approach, behaviour change techniques	Experimental group 1 - motivational intervention for nonattenders (those who do not intend to use condoms). Intervention content used decisional balance (generating pros and cons), and focus on past success (to trigger self-efficacy). Participants read four positive outcome expectancies for condom use. Participants then write four additional benefits of condom use, read a written testimony from a peer role model and write about a situation where they overcame self-doubt and succeeded (to boost self-efficacy).<br><br>Experimental group 2 - volitional intervention for intenders. Participants were encouraged to form action plans by specifying and writing exactly when, where, and with whom they planned to use condoms, and for having condoms available when needed. Examples were presented to participants. Barriers to condom use were also presented, such as being under the influence of substances (drugs and/or alcohol). Participants were asked to form strategies to overcome these difficulties and to write them.	Control group only received basic informational website links containing information about condom use. No tailored content, interactive tasks, or behavioural change techniques were applied.	NR
Chernick (2017)	Text messaging Website	Based on a modified Health Belief Model, incorporating education and action components to address barriers	Text messaging (TM) arm received 3 months of unidirectional educational and motivational texts in English or Spanish. Messages included educational and motivational content based on the modified Health Belief Model.	Wallet card advertising a walk in family planning clinic and a standardised monologue by ED physicians about the need for reproductive care.	Written or spoken discharge instructions at the discretion of the attending physician.

		and enablers to contraceptive use.			
Chernick (2022)	Website, videos, text messaging	Social Cognitive Theory Motivational Interviewing	Dr. Erica - a multimedia digital intervention consisting of: An ED based brief intervention (introductory video and 7 minute contraception education video) Followed by 10 weeks of personalised, interactive multimedia text messaging (minimum 56 texts, maximum 121 texts, including interactive algorithms and visual content including GIFs and instagram style content) Optional LIVE Office Hours for real-time Q&A with a sexual health counsellor.	Usual care - participants received only an introductory text message to confirm texting capability for follow up. No additional education or intervention beyond standard ED care.	An introductory text message from the Dr. Erica automated platform to ensure texting capability and facilitate follow-up.
Chernick (2025)	Smart phone application, Text messaging	Social Cognitive Theory, written using the language of Motivational Interviewing, and designed within a user centred design framework using design thinking methodology and agile sprints.	Dr. Eric - an ED based app with five tailored educational sexual health modules, an animated video explaining the texting component, interactive text messages sent once every 10 days for up to 40 texts over 10 weeks. Access to Live Office Hours for questions.	Usual care - standard medical care as determined by the ED provider and an introductory text message for follow up	Standard medical care as determined by the emergency department provider, which typically consisted of referral to a primary care or adolescent provider.
Christensen (2013)	Computer software	Theory of Planned Behaviour and Social Cognitive Theory, while also incorporating insights from neuroscience about the role of emotions in decision making.	SOLVE - a downloadable simulation video game using virtual intelligent agents and interactive narrative. Immerses MSM in a virtual world simulating common obstacles to safer sex. Includes avatar customisation, interactive choice points, and ICAP process (Interrupt, Challenge, Acknowledge, Provide skills). Messages are sex positive, nonjudgemental, and designed to reduce shame by normalising desires and promoting HIV risk reduction skills.	Waitlist control - participants did not play the game during the intervention period; they only completed baseline and immediate post-test measures.	Same baseline measures (for e.g., shame, UAI) and immediate post-test measures.

Cordova (2020)	Smart phone application	Eco developmental theory and empowerment theory	Storytelling 4 Empowerment (S4E) multilevel mobile health intervention.<br><br>Youth completed the S4E intervention on a tablet in the clinic before their health appointment. The app delivered interactive, tailored prevention content based on the participants risk profile, including animated storytelling scenarios and interactive diagrams. It covered three modules: Alcohol and drug use Tobacco STI/HIV prevention The intervention also included a clinician facing app to facilitate risk communication using motivational interviewing scripts and local resources	Enhanced usual practice - the clinics usual services and were given a binder with a printed PDF version of the S4E tobacco module content (epidemiological statistics and prevention health information related to tobacco and cigarette use) to review before their appointment.	Both arms received the clinics usual services, which included primary care, mental health, sexual and reproductive health, substance use prevention, and gender affirming care for youth aged 12-25 years.
Fiellin (2017)	Video game	Social learning theory, self-efficacy, and principles from message framing grounded in prospect theory.	PlayForward - an interactive, two-dimensional, roleplaying adventure video game designed to promote sexual health and reduce risk behaviours. It consists of approximately 16 hours of gameplay. It includes roleplaying narratives and five skill based minigames aimed at reducing sexual risk behaviours and improving knowledge, attitudes, and intentions.	The control condition consisted of 12 commercially available video games (for e.g., Angry Birds, Dragonbox, Subway Surfer) with no content related to sexual health or HIV prevention.	Both arms were delivered on iPad tablets in structured community-based settings.<br><br>Participants played games onsite under observation, and assessments were conducted at baseline, 6 weeks, and at 3, 6, and 12 month
Gerend (2021)	Text messaging Website	Information, Motivation and Behavioural Skills (IMB) model guided intervention development.	Arm A focused primarily on HPV vaccination (information, motivation, behavioural skills to foster uptake).<br><br>Txt2protect (intervention): A text messaging programme centred on HPV vaccination for young sexual minority men, delivering IMB based content:<br>Week1 (Information): Basics of HPV/HPV vaccination (safety, efficacy, dosing), recommendation from study physician, where/how to get the first dose. Week2 (Motivation): Overcoming barriers (misinformation, lack of	Arm B focused on a variety of sexual health practices (for e.g., condom use, PrEP, HIV/STI testing) with only a brief mention of HPV vaccination. Message number and length were matched across arms to equate attention.<br><br>Attention matched control: Text messaging programme on general sexual health (condom use, HIV/STI testing, PrEP), with only brief HPV vaccination mention; content mirrored the structure and volume of Arm A, but HPV was not the central	Daily text messages were sent for the first 3 weeks (Phase1), followed by monthly messages for approximately 8 months (Phase2). Messages were similar in number and length across arms to ensure attention matching. A condition tailored website accompanied the text messages in both arms. Participants completed surveys at baseline, 3week, and 9month follow-ups, and

			provider recommendation), norms, reasons peers chose vaccination, anticipated regret. Week3 (Behavioural skills): Cost/insurance, local clinics and pharmacy finder, talking with providers/parents, action planning for vaccination. Phase2: Monthly booster texts reinforcing Phase1 content. A condition tailored website supported messages (for e.g., essential HPV information, local clinic contacts).	focus. A condition tailored website accompanied messages.	could earn up to US$75 in gift cards for completing these surveys.
Hebert (2018)	Smart phone application	Transtheoretical Model of Behavioural Change and the Theory of Planned Behaviour	miPlan mobile contraceptive counselling app plus routine contraceptive clinic visit. Participants were oriented to a tablet and given time to engage with the miPlan app in the waiting room before their contraceptive clinic visit. The app covered all contraceptive methods, presented effectiveness rates, side effects, images, and short videos.	Routine contraceptive clinic visits without the app.	Routine clinic visit comprising contraceptive counselling by a reproductive health assistant and a clinician. Baseline survey (tablet based) and 3month telephone follow-up survey. Chart review used to document method received at end of the clinic visit. Participant incentives: $10 at baseline and $20 at 3month follow up.
Hightow Weidman (2019)	Website	Integrated Model of Behaviour Theory, which incorporates constructs from several health behaviour theories (for e.g., Theory of Planned Behaviour, Social Cognitive Theory, Health Belief Model).	HealthMpowerment.org (HMP) a mobile optimised, interactive, online intervention including chats with an HIV expert, interactive quizzes, personalised journals, articles and decision support tools.	Information only control website offering culturally tailored HIV/STI prevention articles without interactive features.	Both arms received access to HIV/STI prevention information online. Participants were given an orientation to their assigned website at baseline and could access the site for the full study duration.
Jones (2013)	Smart phone application<br>Text messaging	Sex Script Theory and Barretts Power as Knowing Participation in Change Theory	Love, Sex, and Choices (LSC) a 12-week soap opera video series streamed to smartphones. The series portrayed archetypical women facing high risk relationship dilemmas. Sexual health promotion messages were	12 weekly HIV prevention messages in text format delivered to smartphones.<br>Messages provided guidance on sexual health, condom use, and HIV testing, reviewed for clarity and	NR

		(PKPCT).	embedded in emotionally engaging narratives to model behaviour change.	cultural relevance.	
Kaufman (2025)	Smart phone application	Motivational interviewing, Transtheoretical Model (Stages of Change). Indigenous learning heuristics: the 7 Grandfather Teachings and the Medicine Wheel to structure content around spiritual, emotional, mental, and physical wellness.	Native WYSE CHOICES mobile app (culturally tailored AEP prevention), six interactive sessions. Content included education, risk assessment, decisional balance and readiness to change exercises, goal setting, and culturally affirming materials, interactive quizzes, reflective prompts and vignettes.	Skills focused mobile app (resume writing, financial literacy/budgeting, professional development) delivered over five sessions.	Both interventions narrated by Auntie Amanda (community leader)<br><br>Sessions targeted about 30 minutes each; participants asked to download and engage with their assigned app over the following month.<br><br>Same survey schedule, and incentives (baseline and 1month surveys; compensation for surveys and app engagement).
Kissinger (2023)	Text messaging, Website, telephone calls and email for reminders and support	Social Cognitive Theory; Theory of Gender and Power.	BUtiful - internet delivered unintended pregnancy and STI prevention programme. Delivered over eight successive, interactive online sessions delivered asynchronously via a website. Content includes contraception options, STI and HIV information and prevention, communication and negotiation skills, relationships, and values and goals, presented through videos, text, interactive activities, message boards, and five female characters including a moderator providing medically accurate information.	Attention control - general health and nutrition curriculum comprising eight sequential interactive online sessions, with matched delivery format and activities, including video content with four female characters and an older moderator.	Password protected website access with technical assistance. Staff contact for reminders and logistics via text messages, telephone calls, and email during the four-week intervention period.<br><br>Quarterly newsletters reinforcing session material after the four-week access window. Financial incentive of 5 dollars per completed session for both arms; visit compensation at baseline, two months, six months, and twelve months.

Manlove (2020)	Smart phone application<br>Text messaging	Theory of Planned Behaviour Constructs of Self efficacy and Vicarious Learning from Social Learning Theory	Pulse reproductive health app compising six interactive sections covering birth control methods, healthy relationships, sexual consent, anatomy and physiology, pregnancy, STIs, and clinic access Includes videos, appointment reminders, and a clinic locator	General health app (focused on topics such as exercise, healthy eating, sleep, and emotional health). Similar design and aesthetics to Pulse but with no sexual or reproductive health content	Preprogrammed text messages every few days with approved content Reminder messages to view the app and complete follow-up surveys
Martinez Garcia (2023)	Smart phone application	Theory of Planned Behaviour, plus self-efficacy and vicarious learning (a key construct within Social Cognitive Theory).	Crush - self-led, web based mobile sexual and reproductive health application for adolescent girls delivering bilingual (English/Spanish) content on contraception methods, STIs, health clinic navigation, healthy relationships, and pregnancy via animations, videos, audio dialogues, comic stories, graphics, and quizzes; content reviewed for scientific accuracy by CDC subject matter experts; self-led use on smartphones.	An existing free nutrition and physical fitness application designed for adolescent women accessed via smartphone.	Participants retained access to their assigned app for 6 months; online surveys at baseline, 3 months, and 6 months.<br><br>Multimedia messages every three days for 6 months with study information, motivational messages, content boosters, and reminders to use the assigned tool. Incentives: Gift cards for survey completion ($10, $10, $15) and an inspirational bracelet at enrolment.
Mustanski (2013)	Website	Information–Motivation–Behavioural Skills model of HIV risk behaviour change, e-learning principles.	Keep It Up! interactive, tailored online intervention. Seven online modules completed across three sessions at least 24 hours apart, using videos, animations and interactive games; content tailored to young men who have sex with men with goal-setting and a 6-week online booster.	Active HIV knowledge control delivered online with the same number of modules across three sessions; didactic, non-interactive, not tailored, presenting general HIV/STI information and condom-use facts.	Online delivery across three sessions; HIV and STI educational content in both arms, including steps for correct condom use; trial conducted after participants received an HIV-negative test result; assessments at baseline and follow-up.
Mustanski (2018)	Website	Information–Motivation–Behavioural Skills (IMB) model of HIV risk behaviour change, informed by principles of e-learning and	Keep It Up! 2.0 interactive online HIV prevention intervention. Seven modules use videos, interactive animations, games, and scenario-based content to increase HIV knowledge, motivate and teach safer behaviours, and build self-efficacy.	eHealth control condition providing HIV/STI information through static text and images. Content reflects generally available online materials and is not tailored to YMSM.	Same number of modules delivered across three sessions at least 24 hours apart.<br><br>Booster sessions at 3 months and 6 months to reinforce content.

		qualitative work with ethnically diverse young men who have sex with men	Three sessions at least 24 hours apart, totalling about one hour, plus booster sessions at 3 and 6 months.	Three sessions at least 24 hours apart with similar time duration to KIU.	Information on biomedical prevention strategies included during booster sessions for both arms.
Mustanski (2025)	Website, Videoconferencing	Information motivation behavioural skills model of HIV risk reduction.	Four intervention arms (embedded regimes):<br><br>**ER1:** Starts with Sex Ed 1.0 - comprehensive sexuality education delivered as an eHealth programme. It was adapted from Queer Sex Ed and aligns with CDC sexual health learning objectives. Content is organised into modules covering: Sexual and gender identity Sexual activity, pleasure, and consent Sex education in the real-world Healthy relationships. Then, SMART Squad an online web series, games, self-assessments, interactive exercises, and resource referrals focussed on changing HIV risk reduction motivation and behavioural skills. This was followed by a Squad booster module if still a nonresponder. Finally, a single booster module.<br><br>**ER2:** Starts with Sex Ed 1.0 (as reported above). Then, SMART Squad (as reported above). Finally, SMART Sessions - motivational interviewing delivered via videoconferencing.<br><br>**ER3:** Starts with Sex Ed 1.0 (as reported above). Then, Sex Ed 2.0 adapted from Queer Sex Ed, covering CDC sexual health objectives. Finally, SMART Sessions (as reported above).<br><br>**ER4:** Starts with Sex Ed 1.0 (as reported above). Then Sex Ed 2.0 (as reported above). Finally, SMART Squad (as reported above).	No control.	Universal first step for all participants: SMART Sex Ed 1.0.<br><br>Stepped care design with eHealth delivery and bilingual English/Spanish content across interventions.

Patel (2024)	Website	Peer based, narrative communication approach	Get It? – a peer based sexual health intervention delivered on an electronic tablet during the clinic visit in a private clinic room.<br><br>The intervention consists of four short videos, each featuring a teenage narrator describing a true peer story about LARC selection, insertion, benefits, and adverse effects.	usual care in the Teen Health Clinic without exposure to Get It?<br><br>Usual care at the Teen Health Clinic without exposure to Get It? participants completed the study surveys and received routine care with providers trained (in both arms) to defer contraceptive counselling until the end of the visit.	Providers trained to delay routine contraceptive counselling until the end of the visit to allow participants time to initiate LARC discussion;<br><br>Face-to-face preintervention survey; written informed consent (parental consent for minors);<br><br>Participants reimbursed. Sixmonth chart review for LARC uptake.
Reback (2024)	Smart phone application<br>Text messaging<br>Website	Stepped care design.	Text+ stepped care. Participants received three months of tailored text messaging. At the three-month assessment, those who did not improve on primary outcomes were stepped up to virtual coaching in addition to continuing text messaging. Responders continued with text messaging only. Access to the initial text condition was maintained for the duration of the study	Webapp+ stepped care. Participants received three months of access to a smartphone optimised web-based intervention. At the three-month assessment, those who did not improve on primary outcomes were stepped up to virtual eCoaching in addition to continuing webapp access. Responders continued with the webapp only. Access to the initial webapp condition was maintained for the duration of the study.	All participants completed baseline and follow up ACASI surveys, with follow-ups scheduled at three, six and nine months. HIV and STI testing were integrated into study procedures, shifting to mailed self-collection kits during the pandemic. Participants received compensation for timely return of HIV testing samples. Regardless of response status, participants maintained continued access to their initially assigned condition throughout the study.
Reiter (2023)	Text messaging<br>Website	Protection Motivation Theory; Information Motivation Behavioural Skills Model; Minority Stress Model	Experimental group 1 - Interactive intervention:<br>Outsmart HPV online content via a project website plus monthly interactive text message reminders. These reminders allowed participants to respond, triggering additional automated tailored messages and resources. Some messages included memes or GIFs to enhance	Standard online HPV vaccine information modelled on the CDC Vaccine Information Statement. No vaccination reminders were provided.	Both arms received identical, population targeted, individually tailored Outsmart HPV web content delivered via the project website.<br><br>Participants could access the website content throughout the study period, except while completing surveys.

			engagement. Participants could also submit questions for manual responses from the study team.<br><br>Experimental group 2 - Unidirectional intervention:<br>Outsmart HPV online content plus monthly unidirectional text message reminders. These were automated messages providing information about HPV and vaccination steps, addressing barriers and concerns, but participants could not reply.		Reminders in both arms were theoretically informed and served as cues to action.
Santa (2021)	Smart phone application	Information, Motivation, and Behavioural Skills model.	MYRID delivered tailored, EMA responsive HIV prevention messages via smartphone for six weeks.<br><br>Intervention participants set an HIV prevention goal at baseline after review of HIV prevention strategies. Following each EMA, they received tailored messages addressing their selected goal areas: safer sexual behaviours, alcohol or drug use, PrEP interest, and HIV testing. Message selection was algorithmic and responsive to current risk predictors captured by EMAs, including sexual urge, stress, and substance use. A graphic goal interface in the app provided real-time behavioural feedback and goal attainment information accessible at any time.	Attention control – participants set a general health behaviour goal at baseline. Following each EMA, they received nontailored messages related to healthy nutrition, physical activity, sleep hygiene, and tobacco prevention. These control messages were sent in a random order and were not responsive to EMA reported risk factors.	Android smartphone with unlimited data<br><br>Orientation to ecological momentary assessment procedures, including setting wake and sleep times to avoid disturbance.<br><br>Daily EMAs over six weeks: three prompts per day in weeks 12, two per day in weeks 34, one per day in weeks 56. Behavioural goal setting at baseline; goals differed by arm but the process of setting a goal was common.<br><br>Incentivised participation with app-tracked compensation contingent on EMA completion.
Schnall (2022)	Smart phone application, web enabled device	Based on an adapted social personal theoretical framework for young MSM, building on social	MyPEEPS Mobile intervention for 3 months. Participants completed 21 online psychoeducational and skill building modules delivered through games, scenarios, and roleplays. Content included HIV/STI education, condom use skills, emotion regulation,	Delayed intervention - no access during the first 9 months; received access after the 9-month follow-up assessment.	Both arms received standard study procedures (baseline and follow up assessments, incentives, and remote contact via study staff). No additional HIV prevention counselling or external

		learning theory and focusing on psychosocial and contextual risk factors related to HIV risk among MSM.	communication strategies, and goalsetting activities.		intervention was provided during the trial period.
Schnall (2025)	Smart phone application<br>Text messaging	NR	Experimental group 1 -<br>mLab App intervention (app + HIV home test kits). The app promoted self-testing and delivered HIV prevention information via email, text and push notifications. It included an embedded smartphone image processing algorithm to interpret OraQuick home HIV self-tests, automated data collection and real-time reporting to the research team, prompts to encourage repeat testing, and linkage messages for confirmatory testing and treatment after reactive results or PrEP uptake after nonreactive results<br><br>Experimental group 2 - Standard of care (HIV prevention information only) No app, image processing, or home test kits	Control - Participants received mailed HIV home test kits only. No mLab App and no additional HIV prevention information beyond what is standard with the test kit.	Common study procedures: informed consent, scheduled assessments at baseline, 6 months and 12 months, and participant compensation at each visit. Procedures were applied to all arms; arm specific supports differed by condition
Starosta (2016)	Website	Theory of Planned Behaviour.	Participants engaged in a decisional balance exercise focused on condom use. They reviewed each sexual act reported and listed reasons for condom use and non-use. Rated the importance of these reasons. Compared ratings for use vs. nonuser. Finally, participants wrote a testimonial encouraging high school girls to insist on condom use, using CDC fact sheets for support	Participants completed the same decisional balance exercise, but focused on binge drinking (defined as more than four drinks on a single occasion). They then wrote an essay encouraging young girls to engage in safe drinking behaviours, using CDC fact sheets for support	NR
Stephenson (2020)	Website	Human-centred design and collaboration with target users.	Interactive website - Contraception Choices. Key components included: Method pages with succinct pros and cons; tips for use. An interactive	Waiting list control receiving usual care. Participants were offered access to the Contraception Choices	Usual contraceptive care from recruiting services (general practice; sexual and reproductive health clinics;

		Tailoring to increase relevance for learning, engagement and behaviour change. Health Belief Model. Social Cognitive Theory. COMB model (capability, opportunity, motivation). Social determinants of health framework.	effectiveness infographic showing typical use pregnancy risk. Videos of young women and clinicians addressing concerns and misperceptions. Did you Know? And FAQs sections tackling common worries and benefits. Tailored decision aid (What's right for me?) that elicits seven preference attributes, highlights suitable methods, ranks top three choices side by side and allows export of results by email or text. Links to NHS resources and clinic finders	website offered after completion of six-month data collection.	maternity service; community pharmacy; abortion service).<br><br>Baseline online questionnaire; follow-up online outcome surveys at three and six months covering current method, satisfaction, pregnancy and STI diagnoses. Automated contact to maximise follow up (emails, text messages, telephone reminders) and electronic vouchers (£5 at three months; £15 at six months).
Tan (2022)	Website, videos	NR	Participants received a link to a series of six online videos from People Like Us, each about ten minutes in duration, plus a link to an English language online sexual health pamphlet tailored for GBMSM in Singapore.	Online sexual health pamphlet only.<br><br>During the trial period, no web drama videos were provided. At the six-month survey, the control group was shown links to all six episodes to assess prior viewing and potential contamination.	Both arms received their assigned materials within one week after completing the baseline survey. Both arms were asked to complete a quiz one week after assignment to verify exposure to their allocated materials. Follow up surveys at three and six months were administered online, with participant contact coordinated by Action for AIDS Singapore and reimbursements provided for survey completion.
Whiteley (2018)	Text messaging, Website	Information Motivation Behavioural skills (IMB) model.	Participants received emails with links to publicly available online HIV/STI prevention content selected for urban minority youth. Delivery comprised eight emails, each containing 23 links, sent twice weekly over four weeks. Content drew from nine different websites and four YouTube videos, totalling 19 links, covering topics such as puberty, anatomy, HIV/STI information, contraception, PrEP/PEP,	Waitlist - No intervention emails during the study period. The control group received the same online materials by email only after the final assessment.	ACASI assessments conducted in research offices at baseline and 12 weeks after the intervention period, monitoring by trained research assistants, and reimbursement for pretest and post-test assessments.

			personal risk assessment, peer norms, condom skills, communication skills, and substance use risks. Participants texted back an acceptability rating after opening the emails.		
Ybarra (2017)	Text messaging	Information Motivation Behavioural Skills Model of HIV preventive behaviour	Guy2Guy (G2G) - a text messaging-based HIV prevention programme tailored for sexual minority adolescent males. Intensive HIV prevention programme delivered via text messages, including:<br>HIV information (what it is, how to prevent it) Motivation (reasons for condom use) Behavioural skills (correct condom use) Additional topics: HIV testing, healthy relationships, coming out, bullying Interactive features: badges, level up quizzes, Text Buddy, and G2Genie (OnDemand advice)	Attention matched Healthy Lifestyle programme general health topics such as self-esteem, positive body image, and bullying. No interactive features (badges, quizzes, Text Buddy, G2Genie) were included.	Both arms utilised text messaging as the delivery method, based on evidence that adolescents are prolific users of mobile phones and text messaging for behaviour change interventions.
Ybarra (2021)	Text messaging	Information Motivation Behavioural Skills (IMB) model	Girl2Girl – a 20 week text messaging-based pregnancy prevention and healthy sexuality programme aimed at reducing teen pregnancy risk among cisgender sexual minority girls.<br>Content included: Pregnancy prevention information (how pregnancy happens and can be prevented)<br>Motivations to avoid pregnancy (reasons to use condoms) Behavioural skills (how to negotiate condom use)<br>Topics relevant to sexual minority girls (for e.g., sexual identity development)<br>Interactive features: Text Buddy peer support, badges for achievements, links to videos, and G2Genie (OnDemand advice).)	Attention matched control programme - messages on general health topics such as exercise and bullying, with two days of pregnancy prevention content to maintain study blinding. No interactive components (for e.g., Text Buddy, badges) were provided.	Both arms received a similar number of messages over a similar duration to maintain equivalence in attention and engagement

b) Substance use interventions

Author (year)	Mode of delivery	Theoretical model	Intervention	Control	Background support to both groups
Fang (2010)	Computer software Website	Family Interaction Theory	Nine-session, web-based substance use prevention programme for mother–daughter dyads, with voice-over narration, animated graphics, games, skill demonstrations, and interactive exercises. One session per week, each 45 minutes.	Test-only control arm; participants completed baseline and follow-up surveys but received no intervention.	Online baseline and follow-up surveys; incentives provided for completing measures.
Gilmore (2023)	Website	Social norms theory combined with a motivational interviewing approach.	Teen Well Check (TWC) web based integrated prevention programme. web-based programme consisting of three interactive modules addressing: Substance use (personalised normative feedback, psychoeducation, video content). Sexual risk behaviours (communication, condom use, impact of substances). Sexual assault prevention (comic art scenarios, consent education, bystander intervention).	Assessment only control - participants completed surveys without receiving any intervention content.	Both arms received baseline and follow-up assessments at 1, 3, and 6 months.
Mason (2015)	Text message	Motivational Interviewing (MI) integrated with a social network counselling model, adapted into a text-based format.	Automated, personalised, interactive text messaging intervention based on motivational interviewing and social network counselling. Messages focused on likes/dislikes about smoking, normative comparisons, network quality, and plans for behaviour change.<br><br>5 consecutive days of text messaging, plus optional booster messages on request.	Attention control condition<br><br>30 health-based text messages covering topics such as diet, exercise, and study habits, matched in length and frequency to the intervention arm.<br><br>5 consecutive days of text messaging. No booster messages.	All participants were provided with smartphones (with unlimited texting, Internet, and limited voice minutes) for the duration of the study and were trained on responding to texts and completing follow-up surveys.
Mason (2016)	Text message	Motivational Interviewing (MI) principles and integrated a social network counselling or peer network counselling	Automated, personalised text messaging intervention integrating motivational interviewing and social/peer network counselling. Messages focused on rapport building, feedback on tobacco use, normative	Attention control condition<br><br>30 health-based text messages covering topics such as diet, exercise, and study habits, matched in length and frequency to the intervention arm.	All participants were provided with smartphones (with unlimited texting, Internet, and limited voice minutes) for the duration of the study and were trained on responding to

		approach.	comparisons, peer network reflection, and planning for behaviour change.<br><br>Booster messages available upon request.	5 consecutive days of text messaging. No booster messages.	texts and completing follow-up surveys.<br><br>Both arms received 30 text messages over 5 days, ensuring equal contact time.
Prokhorov (2008)	Computer software CD ROM	Social Cognitive Theory and the Transtheoretical Model of Change.	ASPIRE, a computer based interactive, multimedia smoking prevention and cessation curriculum delivered on classroom desktop computers.<br><br>It tailors content to each participants smoking status and stage of acquisition or cessation at the start of each session, then provides activities designed to move smokers toward cessation and nonsmokers toward reduced likelihood of initiation. It uses embedded animations, video, and interactive activities.	Standard care - National Cancer Institute's Clearing the Air self-help booklet.	Baseline assessment used an 87item self-report questionnaire; primary follow-up at 18 months.<br><br>Conducted within routine school settings; no additional cointerventions beyond the assigned condition were reported.
Schinke (2005)	Computer software CD ROM<br><br>Parent component included videotape, print materials, newsletters, workshop, and podcasts.	Youth components used Social cognitive theory, problem behaviour theory.<br><br>Parent component used family interaction theory.	Youth CD-ROM intervention plus parent component (CDP). Youths completed a 10 x 45-minute sessions using an interactive CD-ROM programme covering goal setting, coping, peer pressure, refusal skills, norm correcting, self-efficacy, problem solving, decision making, communication, and time management.<br><br>Parents received a 30-minute videotape and print materials initially, plus newsletters and two booster sessions (workshop and interactive CD-ROM with their child).<br>Boosters provided over follow-up years.	Youth CD-ROM intervention only without parent component. Annual booster sessions for youths only.	All arms received pretest and post-test measurements
Schinke (2009)	Website and CD-ROM	Family interaction theory	Computer-delivered prevention programme for adolescent girls and their mothers. Mother–daughter dyads engaged in nine initial interactive sessions delivered via computer (Internet or CD-ROM), plus annual booster sessions. Content included	Test-only control - baseline and follow-up measures only, no intervention content.	All participants completed baseline measures and two annual follow-up assessments; incentives (online/in-store coupons and gift certificates) were provided

			communication, conflict management, substance use education, refusal skills, coping, body image, and self-efficacy.<br><br>Nine weekly sessions, each 45 minutes; annual booster sessions also 45 minutes.		after each data collection occasion.
Schwartz (2014)	Website	Health behaviour theory and the notion of a teachable moment during puberty, when girls may be more receptive to risk information about breasts and femininity.	Targeted, gender and Aboriginal status sensitive, breast cancer specific web message. Visuals showed four girls holding bras with the headline Smoking affects more than your lungs. Core message: Calls to action included avoiding places with second-hand smoke and considering quitting if the recipient smokes. For girls self-identifying as Aboriginal, the identical message included a feather watermark.	Standard Health Canada comparator message about second-hand smoke containing more than 50 cancer causing agents.<br><br>The content was general carcinogen information and did not mention breast cancer specifically.	Immediate post message prompt asking if the participant wanted more information; those answering Yes received additional written information at the end of the survey. The prompt was worded to match each arms topic focus, but the support procedure was the same.<br><br>Follow up survey conducted six months after baseline.
Schwinn (2010)	Website.	Social Learning Theory with a social competence and skills-building approach.	RealTeen - gender-specific, web-based prevention programme.<br><br>Content delivered through interactive sessions covering goal setting, decision making, coping with stress and puberty, self-esteem, assertion and communication, media influences, peer pressure, drug knowledge and refusal skills. Sessions included brief lessons, interactive exercises, quizzes and were guided by an animated young adult female. The site offered optional social features such as personal blogs, a pen-pal pairing and a moderated chat forum.<br><br>Twelve sessions, about 25 minutes each, typically completed in about 1 month, with most completing within 2 months.	Assessment only.	Online pretest and scheduled follow-up measurements administered on a secure study website. ,<br>Routine reminders to complete surveys, for example postcards, emails and telephone calls or equivalent online prompts.<br>Participant reimbursement for completing outcome measurements at specified time points. ,

Schwinn (2015)	Website	Social competency skill-building strategy and minority stress theory guided the intervention content and activities.	Tailored, web-based drug abuse prevention programme for sexual-minority youths, delivered in three sessions led by an animated young adult narrator, with interactive games, role-playing and writing activities. Sessions covered identifying and managing stress, decision-making, drug use rates and refusal skills. Three sessions, each approximately 14 minutes.	Assessment only	Online pretest, posttest and 3-month follow-up assessments, email contact for study links, and incentive e-gift cards at each measurement point.
Schwinn (2021)	Smart phone application	Social Learning Theory, Motivational Interviewing (MI) principles, Bicultural Competence framework	"Vamos" skills-based prevention programme. Sessions focused on refusal skills, goal setting, media literacy, coping, managing mood, and self-efficacy. Each session included: skill description by narrators (two agemate peers), practice exercises and feedback on exercises	Assessment only.	Baseline and follow-up online surveys (post-test, 1, 2, and 3year follow-ups)<br><br>Incentives for completing surveys ($25 for baseline/post-tests; $50 for each follow-up) Identical correspondence for measurement completion and tracking.
Wall (2024)	Website	Psychoeducational principles, combining structured alcohol education with coping skills training.<br>CBT informed. The alcohol component is based on the Brief Alcohol Screening and Intervention for College Students (BASICS) harm reduction approach, and Individual Coping and Alcohol Intervention Program (ICAIP)	Alcohol and Coping - web based, therapist assisted, self-directed intervention.<br><br>Three film-based lectures on family alcohol problems, dependency and risks, family adaptation patterns, attitudes to alcohol and physiological effects (each lecture about 8-15 minutes). Self-assessment of personal drinking (frequency and intoxication) with automated tailored feedback; participants were asked to take a 12-day break. Twenty item coping questionnaire with automated tailored feedback from a prewritten feedback library targeted to responses. Five-minute lecture on emotion and problem focused coping in families with alcohol problems. Four vignette exercises (each introduced by a 12-minute film),	Waitlist control. Participants completed the same assessment schedule but did not receive the intervention during the trial; they were informed they would gain access to Alcohol and Coping after the last follow up assessment.	All participants received signposting to external support resources: a health information website (drugsmart.se) with general and specific content about alcohol and drugs, plus telephone numbers and contact information for organisations and primary health care facilities

			asking participants to propose coping strategies for fictional cases, followed by a reflective exercise on their own family situation and coping.<br><br>Therapist composed personalised feedback (sent after a short break), including reinforcement, coping suggestions, encouragement to seek social support and signposting to services; those disclosing risky alcohol use were encouraged to view two additional short lectures on intoxication (about 4 minutes) and alcohol use/dependency (about 5 minutes)		
Walton (2010)	Computer software	Motivational interviewing, implemented with harm reduction principles, and incorporating normative resetting plus skills practice in alcohol refusal and conflict resolution.	Experimental group 1 – a therapist delivered brief intervention facilitated by a tablet laptop showing tailored feedback. Contents included: goals, personalised feedback on alcohol, violence and weapon carriage, decisional balance exercises, tailored role plays (anger management, conflict resolution, alcohol refusal, not drinking and driving), and referral.<br><br>Experimental group 2 - a standalone interactive computer programme. Guided by an animated character that provided audio feedback and walked participants through the same core components: goals, personalised feedback, decisional balance, and interactive roleplay scenarios requiring behavioural choices about drinking and fighting, plus referral.	Brochure providing community resources, received during the emergency department visit. No brief intervention was delivered beyond receipt of materials.	Brochure with community resources.<br><br>Sessions were delivered prior to emergency department discharge, with privacy ensured, and could be paused and restarted to avoid interference with medical care.<br><br>Computerised baseline assessment for all trial participants, with follow up assessments at 3 and 6 months.
Walton (2013)	Computer software	Motivational Interviewing was the core theoretical model for both active interventions. Therapist delivery used an elicit	Experimental group 1 -<br>Therapist delivered Brief Intervention (TBI): Conducted by research therapists trained in Motivational Interviewing, facilitated by onscreen prompts to standardise content. Therapists reviewed tailored feedback	Enhanced usual care control: A trifold brochure handed to participants containing warning signs of cannabis problems, resources including substance use treatment, suicide hotlines, employment services and leisure activities, and cannabis	All participants completed a computerised baseline survey and post-test during the primary care visit, and computerised follow-ups at 3, 6 and 12 months, with remuneration and urine drug

		framework and cognitive behavioural coping skills manuals.	for cannabis, alcohol and other drug use, consequences and driving under the influence; guided a decisional balance exercise specific to cannabis; and ran role plays to elicit and strengthen refusal skills, safe travel planning, coping with boredom, anger or sadness, and problem identification and help seeking. Session fidelity was monitored via audiotaping and competency/adherence rating.<br><br>Experimental group 2 -<br>Computer based Brief Intervention (CBI): A standalone interactive animated programme on a tablet with touch screen. A virtual buddy guided the participant, delivered audio feedback, and tailored content. During the decisional balance, participants selected reasons to avoid cannabis and received affirmations and summaries. Animated roleplay scenarios asked for behavioural choices, prompted reflection on consequences when a risky option was chosen, then modelled the positive choice once selected. Six characters illustrated progression of medical, social and legal consequences.	information websites.	screens conducted to support data validity.<br><br>A trifold brochure of warning signs, resources and cannabis information websites is listed as content in the BI overview and is the control condition handout, indicating this brochure was a standard resource within the study context.
Walton (2014)	Computer software	Motivational Interviewing, harm reduction orientation and SBIRT context.	Experimental group 1 -<br>Computer brief intervention, CBI. A standalone, interactive, animated programme on a touchscreen tablet, guided by a virtual buddy with audio via headphones. The CBI delivered tailored feedback on cannabis, alcohol, and other drugs, decisional balance exercises, normative resetting, and six gender and risk profile selected roleplays that modelled refusal skills, coping with boredom or negative affect, avoiding delinquency, and safe	Enhanced usual care control, brochure. Participants were given a trifold brochure with warning signs of cannabis problems, community resources, and credible information websites. Clinics did not routinely provide this information prior to the study.	A brochure listing warning signs of cannabis problems and local resources.<br><br>Interventions were conducted in primary care clinics, in private rooms, and could be paused for clinical care; baseline and follow up assessments were self-administered on computers, with staff blinded to condition at follow up

			transport decisions. If a negative choice was selected, the programme linked consequences back to the participants goals and prompted a better choice; summaries reinforced goals and reasons to avoid cannabis, alongside the community resources handout<br><br>Experimental group 2 -<br>Therapist brief intervention, TBI. Delivered by research therapists trained in Motivational Interviewing, facilitated by a computer that displayed tailored feedback and prompted sections. Therapists used summaries, and open questions to evoke change talk, scaling rulers to support self-efficacy and commitment, and conducted roleplays that practised refusal skills, coping with peer pressure and negative affect, harm reduction strategies for transport, and problem identification with options to seek help.		

c) Mental health interventions

Author (year)	Mode of delivery	Theoretical model	Intervention	Control	Background support to both groups
Aboody (2020)	Smart phone application<br>Self-guided	Cognitive Behavioural Therapy (CBT) model targeting maladaptive beliefs associated with body image disturbance.	GGBI mobile app which provided CBT-based cognitive training including psychoeducation focussing on challenging beliefs (e.g. perfectionism, over importance of appearance, body objectification), promoting positive body image, game like exercises and quizzes.<br><br>Push notifications for reminders and progress checks on days 4 and 10.	Waitlist control. No app use during the study period<br><br>Course credits for participating in follow up assessment.	All participants received six course credits for being involved in the trial.<br><br>Two lab sessions for assessments (post-test and 1 month follow up).

			Extra course credits for completing all levels and lottery to win a two-person massage.		
Ampatzoglou 2024	Virtual reality (VR) technology	Cognitive behavioural principles integrated with gamification theory and immersive learning frameworks.	Gamified VR cognitive training program<br><br>Tasks were designed to improve attention, memory, and emotional regulation through interactive scenarios. Intervention delivered via VR headsets with games like challenges and feedback loops.	Standard psychoeducation sessions focused on anxiety and depression management.	Baseline psychoeducation and mental health support resources (e.g. informational materials and access to school counsellors).
Bauermeister 2022	Text messaging and website	Minority Stress Model and the Transactional Model of Stress and Coping.	Full imi web application and weekly text reminders to log in.<br><br>Designed to affirm LGBTQ+ identity and teach coping skills for minority stress, including:<br>Interactive guides: Gender, Queerness, Stress, and Stigma. Activities: guided breathing, positive reframing, planning exercises, chat interfaces for pronouns, and personalized affirmations.<br>Community content: stories, videos, and images from LGBTQ+ youth.<br><br>External resources integrated within the app.	Asterix - a resource only version of the imi site.<br><br>It provided: Curated links to crisis and noncrisis LGBTQ+ resources (for e.g., Trevor Project, local LGBTQ+ centers, moderated chat spaces). Safety tips for online browsing. No interactive guides or identity affirming activities.	Access to curated crisis and noncrisis resources, including:<br>National Suicide Prevention Lifeline ,Trevor Project resources and LGBTQ+ community support tools.
Egan (2021)	Computer software - web accessible roleplaying game	Theory based and community informed, designed to encourage help seeking behaviours, productive coping, and awareness of web-based resources.	Web accessible, roleplaying game Singularities.<br><br>Players assume the role of a superhuman student facing prejudice. The game objectives include identifying problems of nonplayable characters (NPCs), finding resources, and helping NPCs communicate or use resources. The game promotes help seeking, coping skills, and resource awareness.	List of national sex and gender minority (SGM) inclusive resources via email on the day after randomisation.<br><br>After completing the final follow-up survey, they were offered a free download of the game, but no additional follow-up was initiated.	List of national SGM inclusive resources for bullying, child abuse, dating violence, suicide, mental health, substance use, homelessness, and crisis services.
Glass (2022)	Smart phone application and website	Empowerment and on safety planning, informed by prior decision aid trials for abused women,	myPlan – a free, interactive, tailored safety decision and planning tool delivered via mobile app or website.	Access to a secure study website offering a static emergency safety plan and two national resources (a national IPV hotline and a national	Safety planning content online, completed baseline measures, and were assessed immediately after

		evidence that decision aids support complex health and safety decision making.	It includes:<br>Awareness content on myths and healthy relationships, self-assessment with immediate feedback on red flags and danger assessment, interactive visual aid for pairwise comparison of safety priorities (privacy, feelings for partner, safety, children's wellbeing, academics, social support/status), personalised safety plan with information and referrals tailored to user inputs and risk.	LGBTQ hotline). No tailored or interactive components.	first use and again at 6 and 12 months.<br><br>Data collection conducted via the app/website; recruitment, randomisation procedures, and compensation were identical across arms.<br><br>Both arms completed the Preparation for Decision Making Scale after first use and at 12 month follow-up.
Glass (2024)	Smartphone application; Website	Intervention adaptation using a human-centred design approach in partnership with adolescents and youth-serving agencies, guided by essential elements recommended for violence prevention.	myPlan Teen app: A digitally delivered intervention guiding adolescents through app safety features, interactive assessments, activities, and personalised resources for healthy relationships and safety planning.	Standard teen health and safety website offering tools to learn about health topics, including dating relationships.	Access to digital resources and completed surveys at baseline, 3, 6, and 12 months using validated measures.<br><br>Recruitment and retention strategies were applied equally across arms.<br><br>Continuous access after enrolment.
Jeong (2024)	Smart phone application and wearable device (Fitbit Flex FB401)	Concepts of emotional support and informational support as mechanisms to influence depressive symptoms and physical activity.	Emotional and informational support (Experimental Group 1):<br><br>Participants were connected as a group via Fitbit app, exchanged supportive messages daily (for e.g., Cheer up, Fighting), reviewed Fitbit data daily, checked rankings, captured and shared step count screenshots, and searched for records to overcome depressive symptoms.<br><br>Informational support only (Experimental Group 2): Reviewed Fitbit data daily, captured and shared step count screenshots, and searched for records to address depressive symptoms. No group interaction or emotional messages.	Fitbit used without access to data or group interaction.<br><br>Downloaded Fitbit app and connected to Fitbit band.<br>Researchers erased the app and withheld login credentials, preventing participants from accessing their own Fitbit data.	Downloaded the Fitbit application initially.<br><br>Wore a Fitbit device during the intervention.<br><br>Checked CESD scores weekly using the research application.

Rushing (2021)	Text messaging	Community based participatory research with AI/AN youth and experts.	BRAVE intervention: SMS messages designed to improve mental health, help seeking skills, and cultural resilience, including role model videos, mental wellness strategies, and links to culturally relevant resources, plus prompts to engage social support from family and friends	STEM control: SMS messages designed to elevate and reaffirm Native voices in STEM and medicine, closely mirroring BRAVE in content format and frequency so as not to interfere with help seeking skills being modelled in BRAVE.	SMS messages three times per week for 8 weeks, with a similar number of messages and a similar mix of information, role model videos, images, and engagement prompts such as replies for more information, Q&A, and links to resources.<br><br>Recruitment and delivery leveraged the We R Native platform and channels,<br><br>Surveys administered at the same time points.
Shen (2023)	Website	Minority Stress Theory	Project RISE - a single 20-30-minute self-guided online session, including: An introduction to minority stress, privilege, and marginalisation. Psychoeducation on effects of minority stress. Stories from other youths about minority stress experiences. Interactive reflection exercises (identities, emotions, cognitions, values-based needs). Coping statement creation and an action card summarising strategies.	Information only control - illustrated document explaining minority stress theory in age-appropriate language, open ended reflection questions and links to additional online educational resources.<br><br>Received delayed access to Project RISE after completing follow-up.	A resource guide with mental health and LGBTQ+ support resources.

Outcomes

a) Sexual health interventions

**Author (year)**	**Outcome domain**	**Outcomes**
Bauermeister 2019	STI prevention, HIV testing	Mean total number of sexual partners reported at the 90-day follow-up (myDEx (N = 95; M = 1.15(SD = 1.14) versus Control (N = 28; M = 1.50(SD = 2.05); $t_{(153)}$ = − 0.75, *p* = 0.45; Cohen's d = 0.16).<br><br>Condomless receptive anal sex during the 3-month trial period (myDEx26.7% vs. control 45.7%, = 4.40, p = 0.04; Odds Ratio 0.43; 95% CI (0.20, 0.94)).

		Condomless receptive sex with serodiscordant or serounknown partners not known to be on PrEP or virally suppressed (myDEx 8.3% vs. control 17.1%, = 2.18, p = 0.14; Odds Ratio 0 .44; 95% CI (0.15, 1.31)).<br><br>Insertive CAI (myDEx 33.3% vs. control 42.9%, = 1.19, p = 0.27; Odds Ratio 0.64; 95% CI (0.28, 1.44)).<br><br>Insertive CAI with serodiscordant or serounknown partners not known to be on PrEP or virally suppressed (myDEx 10.8% vs. control 20.0%, = 1.86, p = 0.16; Odds Ratio 0.49; 95% CI (0.17, 1.33)).<br><br>HIV testing during 3 month follow up; forty-five percent of the sample (N = 70) with no differences observed between the arms (myDEx 46.7% vs. Control 40.0%; = 0.49, p = 0.49). There were no HIV-positive diagnoses among those tested for HIV.<br>At the 90-day follow-up, participants who did not get an HIV test during the trial (N = 56) did not differ in their intentions to get an HIV test in the following 30 days (myDEx: N = 41; M = 2.59 (SD = 0.97) versus control: N = 15; M = 2.47(SD = 1.06); $t_{(54)} = 0.60$; *p* = 0.70; Cohen's d = 0.18).<br><br>Nine participants reported PrEP uptake during the trial period, with a non-significant trend suggesting those assigned to myDEx were more likely to start PrEP than control (8.9% vs. 3.7%; = 0.91, p = 0.39; Odds Ratio 2.54; 95% CI (0.30, 21.24)).
Bauermeister 2024	STI testing; Health service use	Routine HIV testing (≥2 tests ≥3 months apart); self-report testing behaviour; 12 months; Intervention: 116; Control: 113; OR 1.11; 95% CI [0.69, 1.80]; 51.7% vs 40.9%<br><br>Routine STI testing (≥2 tests); self-report testing behaviour; 12 months; Intervention: 116; Control: 113; OR 0.90; 95% CI [0.54, 1.48]; 31.2% vs 33.6%<br><br>Routine HIV testing (Philadelphia); self-report testing behaviour; 12 months; Intervention: 116; Control: 113; OR 2.48; 95% CI [1.15, 5.33]; 53% vs 31%<br><br>Routine HIV testing (Atlanta); self-report testing behaviour; 12 months; Intervention: 116; Control: 113; OR 0.50; 95% CI [0.24, 1.05]; NR<br><br>Routine HIV testing (Houston); self-report testing behaviour; 12 months; Intervention: 116; Control: 113; OR 1.25; 95% CI [0.37, 4.18]; NR<br><br>Routine STI testing (Atlanta); self-report testing behaviour; 12 months; Intervention: 116; Control: 113; OR 0.35; 95% CI [0.15, 0.81]; 20% vs 41%<br><br>Routine HIV testing moderated by engagement (GC-PLUS, per login); self-report testing behaviour; 12 months; Intervention: 116; Control: NR; OR 1.09; 95% CI [1.01, 1.18]; NR<br><br>Routine STI testing moderated by engagement (GC-PLUS, per login); self-report testing behaviour; 12 months; Intervention: 116; Control: NR; OR 1.11; 95% CI [1.02, 1.21]; NR<br><br>Among those who tested for STIs over the 12-month period:<br>- 41 reported a chlamydia diagnosis (GC-PLUS: *n* = 18; 36.0%; GC-TLO: *n* = 23; 52.3%; $X^2_{(1)} = 2.52$, *p* =.11);

		- 39 were diagnosed with gonorrhea (GC-PLUS: *n* = 23; 46.0%; GC-TLO: *n* = 16; 36.4%; $X^2_{(1)} = 0.90$, *p* =.34);<br>- 21 had a syphilis diagnosis (GC-PLUS: *n* = 10; 20.0%; GC-TLO: *n* = 11; 25.0%; $X^2_{(1)} = 0.09$, *p* =.76);<br>- 13 were diagnosed with herpes (GC-PLUS: *n* = 8; 16.0%; GC-TLO: *n* = 5; 11.4%; $X^2_{(1)} = 0.42$, *p* =.52),<br>- 4 received a genital warts diagnosis (GC-PLUS: *n* = 2; 4.0%; GC-TLO: *n* = 2; 4.5%; $X^2_{(1)} = 0.02$, *p* =.90)
Bennett 2015	HPV vaccination	HPV vaccine uptake (≥1 dose); self-report item; 3 months; Intervention: 330; Control: 331; chi-squared = 0.09, p = 0.76;<br><br>HPV vaccine uptake (≥1 dose) adjuted for covariates; self-report item; 3 months; Intervention: 330; Control: 331; OR 0.92; 95% CI [0.34, 2.54]; uptake 8.4%<br><br>HPV knowledge–uptake association; self-report knowledge questionnaire (10 items); 3 months; Intervention: 330; Control: 331; OR 0.92; 95% CI [0.78, 1.09];
Biello 2021	STI testing	HIV testing (any test during follow-up); self-report; follow-up period NR; App users: 65% ordered HIV self-test kits; Control/Comparator: NR; 75% of those ordered used ≥1 kit; HIV self-tests comprised 70% of all HIV tests;<br><br>STI testing (self-collection kits ordered); self-report; follow-up period NR; App users: 54% ordered ≥1 STI kit;<br><br>STI testing (kits returned to laboratory); self-report/lab records; follow-up period NR; App users: 13% returned ≥1 kit;<br><br>STI positivity (syphilis, gonorrhoea, chlamydia); laboratory confirmed; follow-up period NR; App users: 5.3% of returned samples positive
Biello 2022	STI testing; STI prevention	HIV testing uptake (any test); self-report; 6 months; Intervention: 40, Control:20; MyChoices: 79%; Standard care: 65%; Risk Ratio 1.22; 95% CI [0.85, 1.76]; p = 0.243<br><br>PrEP initiation; self-report; 6 months; Intervention: 40, Control:20; MyChoices: 10.3%; Standard care: 16.7%; Risk Ratio 0.62; 95% CI [0.14, 2.75]; p = 0.662<br><br>Condomless anal sex with non-primary male partners; self-report; 3 months; Intervention: 40, Control:20; MyChoices: 35.1%; Standard care: 40.0%; $\chi^2 = 0.132$; p = 0.716<br><br>Condomless anal sex with non-primary male partners; self-report; 6 months; Intervention: 40, Control:20; MyChoices: 36.4%; Standard care: 35.0%; $\chi^2 = 0.010$; p = 0.920
Biello 2025	Sexual health STI testing; STI prevention	HIV testing (any test); self-report; 6 months; Intervention: 116, Control: 124; MyChoices: 74.4%; Standard care: 58.5%; RR 1.27; 95% CI [1.06, 1.53]; p = .01<br><br>HIV testing (any test); self-report; 6 months; Intervention: 111, Control: 124; LYNX: 70.0%; Standard care: 58.5%; RR 1.19; 95% CI [0.98, 1.44]; p = .08<br><br>PrEP initiation; self-report; 6 months; Intervention: 116, Control: 124; MyChoices: 21.3%; Standard care: 15.6%; RR 1.37; 95% CI [0.79, 2.37]; p = .26<br><br>PrEP initiation; self-report; 6 months; Intervention: 116, Control: 124; LYNX: 19.6%; Standard care: 15.6%; RR 1.26; 95% CI [0.72, 2.21]; p = .42

		HIV testing (time to first test); self-report; up to 12 months; MyChoices vs Standard care; HR 1.45; 95% CI [1.07, 1.95]; p = .015<br><br>HIV testing (time to first test); self-report; up to 12 months; LYNX vs Standard care; HR 1.34; 95% CI [1.00, 1.81]; p = .052<br><br>HIV testing frequency; self-report; 12 months; MyChoices vs Standard care; IRR 1.25; 95% CI [1.01, 1.54]; p = .04<br><br>HIV testing frequency; self-report; 12 months; LYNX vs Standard care; IRR 1.13; 95% CI [0.91, 1.40]; p = .26
Bull 2012	STI prevention	Condom use at last sex; self-report item; 6 months; Intervention: 427; Control: 377; timexgroup interaction F=3.30, p=0.037<br><br>Condom use (simple effects analyses) showed difference between the intervention and control groups at the 2-month follow-up (F=5.37, p=0.02) but that groups did not differ at baseline (F=0.01, p=0.94) or the 6-month follow-up (F=0.03, p=0.86). Condom use at 2 months Cohen's d=0.18.<br><br>Proportion of sex acts protected by condoms; self-report behavioural measure; 6 months; Intervention: 427; Control: 377; timexgroup interaction F=3.63, p=0.027<br><br>>2 sex partners in 2 months; self-report behavioural measure 6 months; Intervention: 427; Control: 377; timexgroup interaction F=1.05,p=0.35<br><br>Drunk or high during sex; self-report behavioural measure 6 months; Intervention: 427; Control: 377; timexgroup interaction F=0.87, p=0.42
Bull 2017	Contraception use; STI prevention; Other	Condoms in past 3 months (sexually active at baseline) Intervention: 27, mean 80.7 SD ±34.2, Control: 28, mean 70.2 SD ±39.0, d= 0.30 (95% CI 0.20, 0.39)<br><br>Condoms in past 3 months (includes abstainers at baseline) Intervention: 217, mean 94.1. SD ±22.1, Control: 200, mean 92.7, SD ±24.1, d= 0.06 (95% CI 0.04, 0.08)<br><br>Contraception in past 3 months (sexually active at baseline) Intervention: 24, mean 92.8, SD ±22.4, Control: 26, mean 80.4 SD ±40.0, d= 0.39 (95% CI 0.30, 0.48)<br><br>Contraception in past 3 months (includes abstainers at baseline), Intervention: 248, Control: 253; mean 97.5. SD ±14.8, Control: 253, mean 95.9 SD ±19.5, d=0.09 (95% CI 0.07, 0.11)<br><br>Access to contraceptive or STD services, Intervention: 313, 5.75%, Control: 311, 8.04%, OR 0.75 (95% CI 0.35, 1.62)<br><br>Ever pregnant or caused pregnancy, Intervention: 256, 3.13%, Control: 255, 3.92%, OR 0.73 (95% CI 0.17, 3.09)
Carvalho 2016	STI prevention	Condom use behaviour; self-report items (6-point scale); 4 weeks; Intervention: 113; between T1 and T3 F = 5.01, partial η^2 = 0.05; between T1 and T2, F = 0.12, ns; between T2 and T3, F= 4.5, partial η^2 = 0.04; Control group; 46, no significant behaviour change between any timepoints.<br><br>Condom use Intervention: 113; Control: 46 mean difference .429 vs. mean difference .477, t, t(97) = 2.21, p .03.

Chernick 2017	Contraception use; Health service use; Other	Effective contraception initiation; EMR documentation and self-report; 3 months; Intervention: 50; Control: 49; 12.0% vs 22.4%<br><br>Family planning clinic attendance; EMR documentation and self-report; 3 months; Intervention: 50; Control: 49; 32.0% vs 30.6%<br><br>Contraception counselling received; EMR documentation and self-report; 3 months; Intervention: 50; Control: 49; 48.0% vs 46.9%<br><br>Pregnancy; EMR documentation and self-report; 3 months; Intervention: 50; Control: 49; 8.0% vs 10.2%
Chernick 2022	Contraception use; Health service use; Other	Highly effective contraception initiation (ITT, imputed); self-report; 12 weeks; Intervention: 72; Control: 74; ARD −0.5%; 95% CI [−13.3, 12.3]; 19.4% vs 18.9%<br><br>Highly effective contraception initiation (ITT, completed follow-up); self-report; 12 weeks; Intervention: 57; Control: 64; ARD 2.7%; 95% CI [−12.4, 17.8]; 24.6% vs 21.9%<br><br>Highly effective contraception initiation (per-protocol); self-report; 12 weeks; Intervention: 48; Control: 64; 27.0% vs 22.0%<br><br>Emergency contraception use; self-report; 12 weeks; Intervention: 38; Control: 50; ARD −20.9%; 95% CI [−37.8, −3.9]; 13.1% vs 34.0%<br><br>Any sex over past 3 months; self report; 12 weeks; Intervention: 49; Control: 59; ARD -3.4; 95% CI [-18.3, 11.3]; 79.6% vs 83%<br><br>Consistent condom use; self report; 12 weeks; Intervention: 39; Control; 49; ARD 7.2 95% CI [-10.2, 24.7], 25.6% vs 18.4%<br><br>Condom use at last intercourse; self report; 12 weeks; Intervention: 39; Control: 49; ARD -1.2, 95% CI [-22.2, 19.7] 53.8% vs 55.1%<br><br>Pregnancy; self-report; 12 weeks; Intervention: 49; Control: 59; ARD -1.0 95% CI [-10.6, 8.6], 5.1% vs 6.1%<br><br>Follow up to medical care; self-report; 12 weeks; Intervention: 52; Control: 58; ARD -5.1, 95% CI [-22.3, 12.1] 67.2% vs 72.4%
Chernick 2025	STI prevention	Condom use in prior 4 weeks; self-report behavioural measure; 6 weeks; Intervention: 47; Control: 50; OR 3.57; 95% CI [1.93, 6.60]; p < .001; 82.5% vs 47.5%. At 13 weeks; OR 0.68; 95% CI [0.42, 1.11]; p .12; 34.6% vs 37.3%<br><br>Consistent condom use in prior 4 weeks; self-report behavioural measure; 6 weeks; Intervention: 47; Control: 50; OR 0.95; 95% CI [0.26, 3.49] p .93. At 13 weeks; OR 1.24; 95% CI [0.34, 4.46]; p .75<br><br>Condom use at last intercourse; self-report behavioural measure; 6 weeks; Intervention: 47; Control: 50; OR 0.84; 95% CI [0.21, 3.35] p .81. At 13 weeks; OR 0.60; 95% CI [0.18, 1.99]; p .41<br><br>Abstinent over past 4 weeks; self report; at 6 week follow up; Intervention: 36; Control; 46; 33.3% vs 30.4% p .78. At 13 week follow up; Intervention: 41; Control: 46; 12.2% vs. 19.6% p .35<br><br>Tested for an STI over past 6 weeks; self report; at 6 week follow up; Intervention: 35; Control: 45; 25.7% vs 40%, p .18. At 13 week follow up; Intervention: 42; Control: 46; 63.9% vs 63%, p .93.

		Tested for HIV over past 6 weeks; self report; at 6 week follow up; Intervention: 35; Control: 46; 22.9% vs 28.3%, p .58. At 13 week follow up; Intervention: 41; Control: 46; 22% vs 32.6%, p .27
Christensen 2013	STI prevention	Unprotected anal intercourse; self-report count (past 3 months); 3 months; Intervention: 437; Control: 484; indirect effect point estimate -0.10; 95% CI [0.01, 0.23]. Direct effect not significant
Cordova 2020	STI prevention; STI testing<br><br><br><br><br><br><br><br><br><br>Smoking; Alcohol; Cannabis	Condomless sex (past 30 days); self-report item; 30 days; Intervention: 21; Control: 21; reduction 10% vs 5%; (χ^2_2=0.2; *P*=.91) Cohen *h*=0.18, 95% CI -0.38 to 0.74<br><br>Alcohol use before sex; self-report item; 30 days; Intervention: 21; Control: 21; reduction 5% vs 0%; (χ^2_2=2.3; *P*=.32) Cohen *h*=0.44, 95% CI -0.12 to 1.00<br><br>Drug use before sex; self-report item; 30 days; Intervention: 21; Control: 21; reduction 10% vs 10%; χ^2_2 NR; Cohen *h* NR<br><br>STI/HIV testing uptake; self-report item; 30 days; Intervention: 25; Control: 25; 52% vs 44%; (χ^2_1=0.3; *P*=.57) Cohen *h*=0.16, 95% CI −0.39 to 0.72<br><br>Any substance use (ATOD); self-report item; 30 days; Intervention: 25; Control: 25; reduction 12% vs 0%; Cohen h=0.71, 95% CI 0.15 to 1.27<br><br>Alcohol use; self-report item; 30 days; Intervention: 25; Control: 25; reduction 12% vs 0%; (χ^2_2=3.9; *P*=.14) Cohen *h*=0.71, 95% CI 0.15 to 1.27<br><br>Tobacco use; self-report item; 30 days; Intervention: 25; Control: 25; reduction 8% vs 4%; (χ^2_1=0.4; *P*=.50), Cohen *h*=0.17, 95% CI -0.39 to 0.73<br><br>Other drug use (including cannabis); self-report item; 30 days; Intervention: 25; Control: 25; reduction 12% vs increase 8%; (χ^2_2=2.9; *P*=.23) Cohen *h* =1.28, 95% CI 0.72 to 1.84
Fiellin 2017	STI prevention;	Delay of initiation of sexual intercourse; self-report item; 12 months; Intervention: 129; Control: 129; RR 0.99; 95% CI [0.94, 1.05]; 94.6% vs 95.4%. Sensitivity analysis suggested no difference in these proportions (missing=delay, P=.90; missing=no delay, P=.78).
Gerend 2021	HPV vaccination	HPV vaccine uptake (≥1 dose); self-report item; 9 months; Intervention: 72; Control: 76; OR 3.43; 95% CI [1.17, 10.08]; p .025; 19.4% vs 6.6%<br><br>HPV vaccine completion (3 doses); self-report item; 9 months; Intervention: 72; Control: 76; OR 1.14; 95% CI [0.08, 16.95]; p .923
Hebert 2018	Contraception use	LARC uptake (any method); chart review; clinic discharge; Intervention: 104; Control: 103; 3.8% vs 1.0% p = .37<br><br>LARC uptake (any method); self-report/chart review; 3 months; Intervention: 88; Control: 78; NR; NR; 8.0% vs 1.0% p = .34<br><br>Implant uptake; self-report/chart review; 3 months; Intervention: 88; Control: 78; 6.8% vs 1.3%, p = .12<br><br>IUD uptake; self-report/chart review; 3 months; Intervention: 88; Control: 78; 1.1% vs 2.6%, p = .60

Hightow Weidman 2019	STI prevention	Condomless anal intercourse (CAI); self-report item; 3 months; Intervention: 238; Control: 236; IRR 0.68; 95% CI [0.43, 0.93]; 32% lower CAI rate in intervention; 6 months; IRR 1.41; 95% CI [0.88, 1.94]; 12 months; IRR 1.09; 95% CI [0.68, 1.50]<br><br>Serodiscordant CAI; self-report item; 3 months; Intervention: 238; Control: 236; IRR 1.37; 95% CI [0.64, 2.10]; 6 months; IRR 1.59, 95% CI [0.74, 2.44]; 12 months IRR 1.23, 95% CI [0.57, 1.89]
Jones 2013	STI prevention	HIV sexual risk behaviour (Vaginal Episode Equivalent, VEE); composite behavioural index; 6 months; Intervention (text): 117; Control (video): 121; reduced VEE in both groups, no between-group difference (Text mean = 1.78, central tendency = 5.93 vs Video mean = 1.58, central tendency = 4.85)
Kaufman 2025	Contraception use	Effective contraception use; self-report item; 1 month; Intervention: 159; Control: 166; (95.9% vs. 89.6%, $\chi2[1] = 6.40$, p = 0.01)<br><br>Past month always used a condom with vaginal sex; self-report item; 1 month; Intervention: 159; Control: 166; (79.8% vs. 71.2%, $\chi2[1] = 4.35$, p = 0.04)<br><br>Past month sexual activity; self-report item; 1 month; Intervention: 159; Control: 166; (77% vs. 90%, $\chi2[1] = 1.54$, p = 0.21)<br><br>Alcohol use with sexual activity; self-report item; 1 month; Intervention: 159; Control: 166; (24% vs. 31%, $\chi2[1] = 0.91$, p = 0.34)
	Alcohol	Alcohol use; self-report item; 1 month; Intervention: 159; Control: 166; (86% vs. 82%, $\chi2[1] = 0.22$, p = 0.64)<br><br>Binge drinking; self-report item; 1 month; Intervention: 159; Control: 166; (43% vs. 47%, $\chi2[1] = 0.18$, p = 0.67)<br><br>Heavy drinking; self-report item; 1 month; Intervention: 159; Control: 166; (11% vs. 21%, $\chi2[1] = 3.27$, p = 0.07)<br><br>Risky drinking; self-report item; 1 month; Intervention: 159; Control: 166; (43% vs. 48%, $\chi2[1] = 0.29$, p = 0.59)
Kissinger 2023	Contraception use; STI prevention; STI testing	Initiation of reliable contraception; self-report item; Intervention: 315; Control: 322; 6 month follow up: RR 1.45; 95% CI [0.99, 2.12], p .06; 12-month follow-up: RR 1.33; 95% CI [0.92-1.91], p .13<br><br>Initiation of dual method use; self-report item; Intervention: 315; Control: 322; 6 month follow up: RR 1.45; 95% CI [1.00, 2.11], p.05; 12-month follow-up; RR 1.31, 95% CI [0.82-2.04], p.3<br><br>Pregnancy; biological/self-report; 12 months; Intervention: 315; Control: 322; HR 0.96; 95% CI [0.44, 2.03];<br><br>STI incidence (chlamydia or gonorrhoea); biological/self-report; 6 or 12 months; Intervention: 315; Control: 322; 6 months RR 0.60, 95% C.I. [0.33-1.1]; at 12 months (6.4% vs.4.7%, RR 1.19 95% C.I. [0.81-1.76]
Manlove 2020	Contraception use; Health service use;	Sex without hormonal/LARC method; self-report; 6 weeks; Total sample: 1087; Intervention: 22.1%; Control: 29.7%; −7.56 percentage points; p = .001<br><br>Sex without any method; self-report; 6 weeks; Total sample: 1086; Intervention: 22.7%; Control: 25.1%; -2.40 percentage points; p = .265;<br><br>Current LARC use (amongst sexually experienced at baseline); self-report; 6 weeks; Total sample 763; Intervention 48.9%, Control 49.1%; -.16 percentage points; p.9

		LARC use at last sex (amongst sexually experienced at baseline); self-report; 6 weeks; Total sample 578; Intervention 49.1% Control 51.7%; -2.62 percentage points, p.4
Martinez Garcia 2023	Contraception use; Health service use	Clinic utilisation (visited clinic in last 3 months for SRH services); self-report; 3 months; Intervention: 595; Control: 615; aOR 1.6 (95% CI 1.0–2.6); p = .05; 6 months; aOR 0.8 (95% CI 0.5–1.3); p = .35;<br><br>Hormonal/IUD use at last sex; self-report; 3 months; Intervention: 595; Control: 615; aOR 0.9 (95% CI 0.4–1.8); p = .67; No significant difference; 6 months; aOR 0.7 (95% CI 0.3–1.6); p = .37; No significant difference<br><br>Engagement in unprotected sex at last sex; 3 months; Intervention: 595; Control: 615; aOR 1.4 (95% CI 0.7–2.9); p = .38; 6 months; aOR 0.9 (95% CI 0.5–1.9) p = .87
Mustanski 2013	STI testing; STI prevention	Unprotected anal sex acts; self-report; 12 weeks; Intervention; 50, Control: 52 KIU!: 44% lower rate; Rate Ratio 0.56; 95% CI NR; p < .05
Mustanski 2018	STI/HIV testing; STI prevention	Any STI (gonorrhoea/chlamydia, urethral or rectal); self-collected specimens; 12 months; Intervention: 359; Control: 374<br>40% (95% CI=5%, 63%, p=0.01) lower in the intervention arm<br><br>Condomless anal sex with casual male partner in the prior 3 months; 12 months; Intervention: 366; Control: 391; 37% vs 44%; prevalence ratio=0.83, 95% CI=0.70, 0.99, p=0.04;<br><br>Number of male casual CAS acts; 12 months; no significant differences<br><br>Number of CAS partners; 12 months; no significant differences<br><br>HIV diagnoses (self reported); 12 months; Intervention: NR; Control: NR; 2.1% (17/793 person years [PY], 95% CI=1.25, 3.43), with no difference between control (8/410 PY, diagnosis rate=2.0%, 95% CI=0.84, 3.85) and intervention arms (9/384 PY, rate=2.3%, 95% CI=1.07, 4.45).
Mustanski 2025	HIV testing; STI prevention	ER1; 252, ER2; 252, ER3; 251, ER4; 253<br><br>Condomless anal sex (self report); baseline to 12 months; ER1: mean difference 0.03 [90% CI −0.02, 0.07], p NR; ER2: mean difference −0.02 [90% CI −0.06, 0.02], p NR; ER3: mean difference 0.04 [90% CI 0.01, 0.08], p < .05; ER4: mean difference 0.03 [90% CI −0.01, 0.07]; p NR<br><br>Casual condomless anal sex (self report); baseline to 12 months; ER1: mean difference −0.05 [90% CI −0.09, −0.02], p < .05; ER2: mean difference −0.04 [90% CI −0.09, −0.01], p < .05; ER3: mean difference 0.0 [90% CI −0.03, 0.04], p NR; ER4: mean difference −0.03 [90% CI −0.08, 0], p < .1<br><br>HIV testing (self report); baseline to 12 months; ER1: mean difference 0.09 [90% CI 0.06, 0.13], p < .05; ER2: mean difference 0.07 [90% CI 0.04, 0.11], p < .05; ER3: mean difference 0.06 [90% CI 0.03, 0.11], p < .05; ER4: mean difference 0.07 [90% CI 0.03, 0.1], p < .05

		PrEP use (self report); baseline to 12 months; ER1: mean difference 0.03 [90% CI 0.03, 0.07], p < .05; ER2: mean difference 0.04 [90% CI 0.03, 0.06], p < .05; ER3: mean difference 0.09 [90% CI 0.06, 0.11], p < .05; ER4: mean difference 0.06 [90% CI 0.04, 0.09], p < .05
Patel 2024	Contraception use	LARC uptake (within 6 months); chart review; 6 months; Intervention: 99; Control: 134; p = .06; 27% vs 9%<br><br>Initiation of LARC discussion with provider; self-report/chart review; 6 months; Intervention: 99; Control: 134; NR; p < .001; 25% vs 9%
Reback 2024	STI prevention	Cumulative condomless encounters (estimated 6 month difference); Text: 71; Webapp: 67; Control: 67; Text+ vs control −0.27 (95% CI −0.68, 0.13), p .181; Webapp + vs. control −0.33 (−0.67, 0.01), p .057<br><br>Cumulative condomless encounters whilst high on drugs or alcohol (estimated 6 month difference); Text: 71; Webapp: 67; Control: 67; Text+ vs control −0.06 (95% CI −0.24, 0.12), p .531; Webapp + vs. control −0.04 (−0.16, 0.24), p .714<br><br>PrEP uptake (estimated 6 month difference); Text: 71; Webapp: 67; Control: 67; Text+ vs control 0.00 (95% CI −0.08, 0.09), p .913; Webapp + vs. control −0.06 (−0.13, 0.01), p .083
Reiter 2023	HPV vaccination	HPV vaccine initiation (≥1 dose); self-report; 9 months; Interactive intervention: 408; Unidirectional intervention: 407 Control: 412; Interactive vs control OR 1.47; 98.3% CI [1.03, 2.11]; uptake 36% vs 28%; Unidirectional vs control OR 1.36, 98.3% CI [0.94–1.95] p .045; uptake 34% vs 28%; Interactive vs unidirectional group OR 1.09, 98.3% CI [0.76–1.54], p .580; uptake 36% vs. 34%;<br><br>HPV vaccine completion (3 doses); self-report; 9 months; Interactive intervention: 408; Unidirectional intervention: 407 Control: 412; Interactive vs control OR 3.70 98.3% CI [1.75, 7.83], p < .001; completion 12% vs 3%; Unidirectional vs control OR 2.26, 98.3% CI [1.02 – 5.00] p .014; completion 7% vs 3%; Interactive vs unidirectional group OR 1.64, 98.3% CI [0.91–2.94], p .45; completion 12% vs 7%
Santa 2021	STI prevention<br><br>Alcohol, drug use	Sexual intercourse (6 week period); self-report; Intervention: 48; Control: 49; timexgroup interaction OR 1.41 95% CI 0.77, 1.43<br><br>Drug use (6 week period); self-report; Intervention: 48; Control: 49; timexgroup interaction OR 0.62, 95% CI 0.39, 0.97<br><br>Alcohol use (6 week period); self-report; Intervention: 48; Control: 49; timexgroup interaction OR 0.87 95% CI 0.55, 1.34
Schnall 2022	STI prevention; HIV/STI testing	Condomless anal sex acts (past 3 months); self-report; 3 months; Intervention: 382; Control (delayed): 381; interventionxtime IRR 0.56; 95% CI [0.32, 0.99]; 6 months; IRR 0.61; 95% CI [0.34, 1.08]; 9 months; IRR 0.83; 95% CI [0.47, 1.47];<br><br>Number of sex partners (past 3 months); self-report; 3 months; Intervention: 382; Control (delayed): 381; interventionxtime IRR 1.16 (95% CI 0.74-1.81); 6 months IRR 0.99 (95% CI 0.63-1.54); 9 months; IRR 1.20 (95% CI 0.76-1.87)<br><br>Number of condomless anal sex partners (past 3 months); self-report; 3 months; Intervention: 382; Control (delayed): 381; interventionxtime IRR 1.09 (95% CI 0.64-1.87); 6 months IRR 0.73 (95% CI 0.43-1.26); 9 months IRR 1.27 (95% CI 0.74-2.17)<br><br>Sex acts using substances (past 3 months); self-report; 3 months; Intervention: 382; Control (delayed): 381; interventionxtime IRR 0.91 (95% CI 0.41-1.99); 6 months IRR 0.91 (95% CI 0.41-1.99); 9 months IRR 1.51 (95% CI 0.71-3.22)<br><br>HIV testing; self-report; 3 months; Intervention: 382; Control (delayed): 381; interventionxtime OR 1.18 (95% CI 0.59-2.35); 6 months OR 1.28 (95% CI 0.64-2.60); 9 months OR 1.72 (95% CI 0.85-3.47)

		STI testing; self-report; 3 months; Intervention: 382; Control (delayed): 381; interventionxtime OR 1.45 (95% CI 0.69-3.02); 6 months OR 1.26 (95% CI 0.59-2.69); 9 months; OR 1.54 (95% CI 0.72-3.28)<br><br>PrEP use; self-report; 3 months; Intervention: 382; Control (delayed): 381; interventionxtime OR 1.65 (95% CI 0.12-23.19); 6 months OR 5.31 (95% CI 0.34-83.90); 9 months OR 3.70 (95% CI 0.23-58.41)
Schnall 2025	STI testing; STI prevention	HIV testing (any test in past 6 months); self-report; 6 months; mLab: 211; Standard care: 208; OR 0.29; 95% CI [0.14, 0.60] p .001; 88.5% vs 65.1%; 12 months; OR 0.17; 95% CI [0.08, 0.37]; p < .001; 90.0% vs 57.7%<br><br>HIV testing (any test in past 6 months); self-report; 6 months; mLab: 211; Mailed test: 104; OR 0.66; 95% CI [0.27, 1.63]; p .37; 88.5% vs 72.3%; 12 months; OR 0.63; 95% CI [0.25, 1.62]; p .34; 90.0% vs 73.9%
Starosta 2016	STI prevention	Condom use (percentage of vaginal sex acts); self-report (TLFB-SS); 3 months; Intervention: 215; Control: 207; no significant overall change from baseline (F(1,160) = 1.759, p = 0.187)
Stephenson 2020	Contraception use; STI testing; Other	Contraception use (LARC use); self-report; 6 months; Intervention: 388, Control: 398; Intervention: 30.4%; Control: 31.0%; aOR 0.87; 95% CI [0.60, 1.28]; p = 0.48<br><br>Contraception use (effectiveness category of method); self-report; 6 months; Intervention: NR; Control: NR; aOR 1.15; 95% CI [0.87, 1.52]; p = 0.33<br><br>Contraception use (change to more effective method); self-report; 6 months; Intervention: 23.8%; Control: 20.5%<br><br>STI diagnosis; self-report; 3–6 months; Intervention: 5.3%; Control: 4.7%; aOR 1.14; 95% CI [0.55, 2.36]; p = 0.72<br><br>Pregnancy; self-report; 6 months; Intervention: 3.3%; Control: 4.1%; aOR 0.90; 95% CI [0.45, 1.79]; p = 0.76
Tan 2022	STI testing; STI prevention	HIV testing (regular testing ≥ yearly); self-report; 6 months; Intervention: 109; Control: 123; Intervention: 60.6%; Control: 44.7%; Risk difference 15.8%; 95% CI [3.2, 28.6]; p = .02;<br><br>HIV testing (tested in the last 6 months); self-report; 6 months; Intervention: 38.5%; Control: 33.3%; Risk difference 5.2%; 95% CI [-7.1, 17.5]; p = .41;<br><br>HIV testing (ever tested); self report; 6 months; Intervention: 76.2%; Control: 77.2%; Risk difference -1.1%; 95% CI [-11.9, 9.9]; p = .85<br><br>Chlamydia/gonorrhoea testing (regular testing ≥ yearly); self-report; 6 months; Intervention: 109; Control: 123; Intervention: 35.8%; Control: 20.3%; Risk difference 15.5%; 95% CI [4.2, 26.9]; p = .009;<br><br>Chlamydia/gonorrhoea testing (tested in the last 6 months); self-report; 6 months; Intervention: 22.9%; Control: 17.9%; Risk difference 5.1%; 95% CI [-5.3, 15.3]; p = .34;<br><br>(Chlamydia/gonorrhoea testing ever tested); self report; 6 months; Intervention: 51.4%; Control: 41.5%; Risk difference 9.9%; 95% CI [-2.9, 22.7]; p = .13

		Syphillis testing (regular testing ≥ yearly); self-report; 6 months; Intervention: 109; Control: 123; Intervention: 39.5%; Control: 29.3%; Risk difference 10.2%; 95% CI [-2.0, 22.4]; p = .010;<br><br>Syphillis testing (tested in the last 6 months); self-report; 6 months; Intervention: 27.5%; Control: 25.2%; Risk difference 2.3%; 95% CI [-9.0, 13.6]; p = .69;<br><br>Syphillis testing (ever tested); self report; 6 months; Intervention: 63.3%; Control: 52.0%; Risk difference 11.3%; 95% CI [-1.4, 24.0]; p = .08<br><br>Inconsistent condom use in last 6 months with casual partners; self report; 6 months; Intervention: 109; Control: 123; Intervention: median 15 (IQR 13.8); Control; median 20 (IQR 16.3); difference -2.5%; 95% CI [–11.7 to 6.7]; p = .60<br><br>Incidence of sexually transmitted infections in last 6 months; self report; 6 months; Intervention: 109; Control: 123; Intervention: median 4.0 (IQR 3.7); Control: median 8.0 (IQR 6.5); difference -2.8%; 95% CI [–8.4 to 2.8]; p = .33
Whiteley 2018	STI prevention	Unprotected vaginal/anal sex (past 3 months); self-report; 12 weeks; Intervention: 31; Control: 29; aOR 7.77; 95% CI NR; p < .05<br><br>Alcohol or drug use before/at sex (past 3 months); self-report; 12 weeks; Intervention: 31; Control: 29; aOR 3.12; 95% CI NR; p NR
Ybarra 2017	HIV testing; STI prevention	Condomless sex acts (past 90 days), all participants; self-report; 90 days; Intervention: 137; Control: 146; aIRR 1.02; 95% CI [0.51, 2.04]; p NR; (intervention end) aIRR 0.58; 95% CI [0.22, 1.50]; p NR;<br><br>Abstinence (past 90 days), all participants; self-report; Intervention: 137; Control: 146; aOR 0.63; 95% CI [0.36, 1.12]; p NR; (intervention end) aOR 1.12; 95% CI [0.60, 2.09]; p NR<br><br>HIV testing, sexually experienced; (n=144) self-report; (intervention end); aOR 3.39; 95% CI [1.52, 7.58]; p < 0.001; (3 month post intervention) aOR 3.42; 95% CI [1.65, 7.09]; p < 0.001
Ybarra 2021<br><br><br><br><br><br><br><br><br><br><br><br>Ybarra 2023 (long term follow up)	STI prevention; Contraception use; Other	Condom-protected penile–vaginal sex acts (past 3 months); self-report; intervention end (5 months); Intervention: 410; Control: 389; IRR 1.48; 95% CI [1.30, 1.68]; P < .001<br><br>Condomless penile–vaginal sex acts (past 3 months); self-report; intervention end (5 months); Intervention: 410; Control: 389; IRR 0.79; 95% CI [0.66, 0.94]; P = .007<br><br>Current use of birth control other than condoms; self-report; intervention end (5 months); Intervention: 410; Control: 389; aOR 1.60; 95% CI [1.08, 2.37]; P = .02<br><br>Abstaining from penile-vaginal sex (past 3 months); self report; Intervention: 410; Control: 389; aOR 0.82; 95% CI [0.55, 1.23]; p .34<br><br>Pregnancy since programme enrolment: self report; Intervention: 410; Control: 389; aOR 0.43; 95% CI [0.11, 1.70]; p .23<br><br>Condom-protected penile–vaginal sex events; self-report; Intervention: 394; Control: 372; (3 month post intervention) IRR 1.16; 95% CI [0.69,1.93] p NR; (6 month post intervention); IRR 1.89; 95% CI [1.07,3.34] P < .05; (9 month post intervention); IRR 1.16; 95% CI [1.06, 1.76] P < .05; (12 months post-intervention); IRR 1.39; 95% CI [1.09, 2.11]; P < .05.

		Use of birth control other than condoms; self-report; Intervention: 394; Control: 372; (3 month post intervention) OR 1.08; 95% CI [0.81,1.45] p NR; (6 month post intervention); OR 1.35; 95% CI [1.22, 1.39] P < .05; (9 month post intervention); OR 1.14; 95% CI [1.02, 1.34] P < .05; (12 months post-intervention); OR 1.07; 95% CI [1.01, 1.26]; P < .05<br><br>Abstinence (self report); Intervention: 394; Control: 372; (3 month post intervention) OR 0.78; 95% CI [0.50,1.12] p NR; (6 month post intervention); OR 1.04; 95% CI [0.30, 1.47] P NR; (9 month post intervention); OR 1.09; 95% CI [0.68, 1.21] P NR; (12 months post-intervention); OR 1.06; 95% CI [0.61, 1.08]; P NR<br><br>Pregnancy (self report); Intervention: 394; Control: 372; (3 month post intervention) OR 0.40; 95% CI [0.12,1.41] p NR; (6 month post intervention); OR 0.75; 95% CI [0.63, 1.84] P NR; (9 month post intervention); OR 0.50; 95% CI [0.15, 1.67] P NR; (12 months post-intervention); OR 0.80; 95% CI [0.30, 1.73]; P NR

b) Substance use interventions

**Author (year)**	**Outcome domain**	**Outcomes**
Fang 2010	Alcohol; smoking; drug use<br><br><br><br><br><br><br>Depression	Alcohol use (30-day use) self-report; post-test at 6 months; Intervention: 54; Control: 50; Wald χ^2 5.85, p .016<br><br>Cigarette use (30-day use) self-report; post-test at 6 months; Intervention: 54; Control: 50; Wald χ^2 3.54, p.06<br><br>Marijuana use (30-day use) self-report; post-test at 6 months; Intervention: 54; Control: 50; Wald χ^2 6.84, p.009<br><br>Prescription drug use (30-day use) self-report, post-test at 6 months; Intervention: 54; Control: 50; Wald χ^2 5.73, p.017<br><br>Depression; self-report scale; post-test (6 months); Intervention: 54; Control: 50; Wald χ^2 3.97; p .045
Gilmore 2023	Substance use	Substance misuse risk (CRAFT score of >2) Intervention: 61; Control 62 1 month follow up Wald χ^2 0.2 (df 1), Cramer V 0.041, p .79; 3 month follow up Wald χ^2 0.9 (df 1), Cramer V 0.104, p .35; 6 month follow up Wald χ^2 1.8 (df 1), Cramer V 0.159, p.17
Mason 2015	Smoking	Cigarettes smoked (past 30 days); self-report; 6 months; Intervention: 37; Control: 35; Significant condition×time interaction ($F(1,55)=4.39$, $p<.01$); partial $\eta^2=0.17$; Mean change: 3.3→2.4 vs 3.3→2.9<br><br>Days smoked (past 30 days); self-report; 6 months; Intervention: 37; Control: 35; No significant condition×time interaction; partial $\eta^2=0.04$
Mason 2016	Smoking	Days smoked cigarettes past 30 days; Intervention 87; Control 85; 1 month follow up intervention group mean (SD) 4.5 (1.7), control 4.4 (1.8), 3 month follow up intervention mean (SD) 4.1 (1.9), control 4.1 (2.0), 6 month follow up intervention mean (SD) 3.4 (1.9), control 3.9 (2.0), eta2 0.02, p <.05<br><br>Cigarettes smoked per day past 30 days Intervention 87; Control 85; 1 month follow up intervention mean (SD) 3.1 (1.2), control 3.0 (1.1), 3 month follow up intervention mean (SD) 3.3 (1.3), control 3.0 (1.3), 6 month follow up intervention mean (SD) 2.7 (1.2), control 3.1 (1.3), eta2 0.10, p <.01

Prokhorov 2008	Smoking	Smoking initiation (baseline non-smokers); self-report item; 18 months; Intervention: 610; Control: 550; OR 2.87; 95% CI 1.08, 7.61, p .05 (1.9% vs 5.8%, $p < .05$)<br><br>Smoking cessation (baseline smokers); self-report item; 18 months; Intervention: 610; Control: 550; OR 1.00; 95% CI 0.30, 2.70; p NR (60.7% vs 61.8%, p NR)<br><br>Smoking intensity; Minnesota Smoking Index (MSI); 18 months; Intervention: 610; Control: 550; lower adjusted mean score in intervention; 0.7 95% CI 0.00, 1,4 vs. 1.7 95% CI 0.9, 2.5, but with overlapping confidence intervals.
Schinke (2005)<br>Schinke (2010)<br>Schwinn (2010)	Alcohol; smoking; drug use	Alcohol use (past-month frequency, heavy drinking, alcohol-related consequences); self-report; post-test to long-term follow-up (up to 6–7 years); Intervention: NR; Control: NR; Significant intervention × time effects and regression coefficients favouring intervention (e.g. alcohol use $B = -1.31$, $p < .01$; binge/heavy drinking $B = -0.57$, $p < .01$; $\beta = -0.48$, $p < .05$);<br><br>Smoking (past-month cigarette use); self-report; post-test to long-term follow-up; Intervention: NR; Control: NR; Lower cigarette use in intervention at long-term follow-up ($B = -1.68$, $p < .05$; $\beta = -4.20$, $p < .05$);<br><br>Cannabis (past-month marijuana use); self-report; post-test to long-term follow-up; Intervention: NR; Control: NR; Smaller increases / lower use in intervention arms over time (MANOVA interaction $p < .001$; χ^2 and regression $p \leq .01$ across reports);
Schinke 2009	Alcohol; smoking; drug use<br><br><br><br><br><br><br><br><br><br><br><br><br><br>Depression	Cigarette use (past 30 days); self-report; 2 years; Intervention: 458; Control: 458; No significant between-group difference F 1.11, p NS<br><br>Alcohol use (past 30 days); self-report; 2 years; Intervention: 458; Control: 458; Significant time×intervention interaction F 5.20, p .006; lower use in intervention arm<br><br>Marijuana use (past 30 days); self-report; 2 years; Intervention: 458; Control: 458; Significant time×intervention interaction F 4.12, p .016; lower use in intervention arm<br><br>Illicit prescription drug use (past 30 days); self-report; 2 years; Intervention: 458; Control: 458; Significant time×intervention interaction F 3.58, p .03; lower use in intervention arm<br><br>Inhalant use (past 30 days); self-report; 2 years; Intervention: 458; Control: 458; Significant time×intervention interaction F 3.72, p .024); lower use in intervention arm<br><br>Depression 5-point survey adapted from Children's Depression Inventory; self-report; 2 years; Intervention: NR; Control: NR; No significant between-group difference F 1.79, p NS
Schwartz 2014	Smoking	Tried smoking between baseline and follow-up; self-report item; ~6 months; Intervention: 242; Control: 376; RR 1.14; 95% CI 0.48, 2.69; p NR

Schwinn (2010)	Alcohol; smoking; drug use	Alcohol; self-report past-month use and binge drinking; 6 months; Intervention: NR; Control: NR; F 4.00, df 1,190, p < .05; Cohen's d ≈ 0.20<br><br>Cigarette use; self-reported past month use; 6 months Intervention: NR; Control: NR, F 0.06, df 1,190, p .82<br><br>Marijuana; self-report past-month use; 6 months; Intervention: NR; Control: NR; F 5.92, df 1,189, p .002<br><br>Poly drug use (cigarettes, marijuana, cocaine, inhalants, methamphetamines, ecstasy) 6 months Intervention: NR; Control: NR, F 6.85, df 1,189, p .01
Schwinn (2015)	Alcohol; smoking; drug use<br><br><br><br><br><br><br><br><br><br>Mental wellbeing	Past 30-day cigarette use; 3 months; Intervention: 97; Control: 103; intervention group mean .72 (SE 1.97) control group mean .90 (SE 2.21) t .59, No significant between-group difference<br><br>Past 30-day alcohol use; 3 months; Intervention: 97 Control: 103; intervention group mean 1.29 (SE 2.15) control group mean 1.10 (SE 2.05) t ,66, No significant between-group differences<br><br>Past 30-day marijuana use; 3 months; Intervention: 97; Control: 103; Intervention group mean 1.63 (SE 1.64) control group mean 1.74 (1.98) t .41, No significant between-group difference<br><br>Past 30-day other drug use (inhalants, club drugs, steroids, cocaine, methamphetamines, prescription drugs, heroin); 3 months; Intervention: 97; Control: 103; intervention group mean 1.03 (SE .12) control group mean 1.09 (SE .22) t 2.16 (p < .05);<br><br>Perceived stress, 3 months; Intervention: 97; Control: 103; d .34 (p < .05)<br><br>Coping skills, 3 months; Intervention: 97; Control: 103; d .32 (p < .05)<br><br>Problem-solving skills, 3 months; Intervention: 97; Control: 103; d .34 (p < .05)
Schwinn (2021)	Alcohol; cigarette; drug use	Posttest: Intervention 255; Control 299; 1 year follow up: Intervention 262; Control 286; 2-year follow-up; Intervention 250; Control 287; 3-year follow-up Intervention; 269, Control 303<br><br>Past-month marijuana use: Posttest OR 0.55, 95% CI 0.23, 1.31, p NR; 1 year follow up OR 0.37, 95% CI 0.13, 1.07, p NR, 2 year follow up OR 0.33, 95% CI 0.12, 0.93, p < .05; 3 year follow up OR 0.27, 95% CI 0.09, 0.80, p < .05<br><br>Past-month use of alcohol from baseline to postest, 1 year, 2 year and 3 year follow up: Posttest OR 0.62, 95% CI 0.31, 1.23; 1 year follow up OR 0.55, 95% CI 0.26, 1.15, p NR, 2 year follow up OR 0.47, 95% CI 0.23, 0.99, p < .05; 3 year follow up OR 0.60, 95% CI 0.29, 1.24, p NR<br><br>Past month cigarette use: Posttest OR 2.14, 95% CI 0.22, 20.94, p NR; 1 year follow up OR 1.01, 95% CI 0.17, 6.58, p NR; 2 year follow up OR 1.14, 95% CI 0.29, 6.81, p NR; 3 year follow up OR 1.12, 95% CI 0.36, 9.19, p NR<br><br>Past-month polydrug use: Posttest OR 0.78 95% CI 0.48, 1.27, p NR; 1 year OR 0.56, 95% CI 0.34, 0.93, p < .05; 2 year follow up OR 0.54, 95% CI 0.32, 0.89, p < .05; 3 year follow up OR 0.55, 95% CI 0.32, 0.93, p < .05

	Depression; anxiety	Depression: Posttest F(1, 548) = 4.67, p < .05; 1 year follow up F(1, 542) = 0.95, p NR; 2 year follow up F(1, 531) = 0.59, p NR; 3 year follow up F(1, 536) = 0.45, p NR<br><br>Anxiety: Postest F(1, 548) = 1.98, p NR 1 year follow up F(1, 542) = 0.85, p NR; 2 year follow up F(1, 531) = 0.38, p NR; 3 year follow up F(1, 536) = 1.07, p NR
Wall (2024)	Substance use<br><br><br><br><br><br>Depression;<br><br><br>Mental wellbeing	Alcohol consumption among drinkers (AUDIT-C, continuous); self-report; 2 months; Intervention: 101; Control: 103; Ratio 0.62; 95% CI [0.44, 0.88]; p NR; self-report; 6 months; Ratio 0.54; 95% CI [0.38, 0.76]; p NR<br><br>Alcohol abstinence (AUDIT-C, binary no drinking); self-report; 2 months; Intervention: 101; Control: 103; OR 1.97; 95% CI [0.25, 19.52]; p NR; self-report; 6 months; OR 3.07; 95% CI [0.40, 40.79]; p NR<br><br>Depression symptoms (CES-DC); self-report; 2 months; Intervention: 101; Control: 103; Cohen's d −0.04; 95% CI [−0.31, 0.23]; p.76; self-report; 6 months; Cohen's d −0.21; 95% CI [−0.48, 0.07]; p .11<br><br>Mental well-being (life satisfaction – Ladder of Life, past/present/future); self-report; 2 months; Intervention: 101; Control: 103; Cohen's d range −0.22 to −0.12; p NS; self-report; 6 months; Cohen's d range −0.08 to 0.06; p NS
Walton (2010)	Alcohol	Alcohol misuse (Audit-C ≥3) baseline to 3 months. Therapist group 104, Computer group 111, Control group 126. % change baseline to 3 months; Therapist –15.6%, Computer – 12.9%, Control – 9.6%. % change baseline to 6 months; Therapist –16.9%, Computer – 12.6%, Control – 12.6%.<br><br>Any binge drinking (self-report) baseline to 3 months. Therapist group 104, Computer group 111, Control group 126. % change baseline to 3 months; Therapist –18.4%, Computer – 19.7%, Control – 19.4%. % change baseline to 6 months; Therapist –20.3%, Computer – 15.5%, Control – 19.9%.<br><br>Alcohol consequences (self-report) baseline to 3 months. Therapist group 104, Computer group 111, Control group 126. % change baseline to 3 months; Therapist –26.6%, Computer – 22%, Control – 17.5%. % change baseline to 6 months; Therapist –32.2%, Computer – 29.1%, Control – 17.7%.<br><br>Alcohol misuse (Audit-C ≥3) Computer Group by Time (baseline to 3 months) RR 0.90 95% CI 0.65,1.24 (baseline to 6 months) RR 0.93 95% CI 0.66, 1.30<br><br>Any binge drinking (self-report) Computer Group by Time (baseline to 3 months) RR 0.93 95% CI 0.66, 1.30 (baseline to 6 months) RR 1.06 95% CI 0.76, 1.47;<br><br>Alcohol consequences (self-report) Computer Group by Time (baseline to 3 months) RR 0.82 95% CI 0.54, 1.23; (baseline to 6 months) RR 0.62 95% CI 0.39, 0.98
Walton (2013)	Alcohol, smoking, drug use	Cannabis use (frequency, past 3 months); self-report; 3 months; CBI: 82; TBI 101 Control: 96 group×time effect estimate –0.08 (SE 0.15) p .57; 6 months; CBI: 79, TBI: 102; Control: 97. Groupxtime effect estimate 0.08 (SE 0.16) p .62; 12 months; CBI: 77; TBI: 104; Control: 94 group×time effect estimate –0.03 (SE 0.16) p .85<br><br>Cannabis-related consequences; self-report; 3 months; CBI: 82; TBI 101 Control: 96 group×time effect estimate −0.24; SE 0.12; p < .05; 6 months; CBI: 79, TBI: 102; Control: 97. Groupxtime effect estimate –0.15 (SE 0.16) p .37; 12 months; CBI: 77; TBI: 104; Control: 94 group×time effect estimate 0.08 (SE 0.17) p .62

		Other drug use (illicit & non-medical prescription drugs); self-report; 3 months; CBI: 82; TBI 101 Control: 96 group×time effect estimate 1.82 (SE 0.68); p < .01, 6 months; CBI: 79, TBI: 102; Control: 97. Groupxtime effect estimate −1.41; (SE 0.52); p < .01; 12 months; CBI: 77; TBI: 104; Control: 94 group×time effect estimate 0.21 (SE 0.48) p .66<br><br>Alcohol use (frequency, past 3 months); self-report; 3 months CBI: 82; TBI 101 Control: 96 group×time effect estimate –0.19 (SE 0.20); p .34, 6 months; CBI: 79, TBI: 102; Control: 97. Groupxtime effect estimate −0.25; (SE 0.20); p .22; 12 months; CBI: 77; TBI: 104; Control: 94 group×time effect estimate -0.16 (SE 0.20) p .44
Walton (2014)	Alcohol; drug use	Any cannabis use (cumulative prevalence); self-report; 12 months; CBI: 16.82%; Control: 24.16%; RR 0.70; 95% CI [0.48, 1.00]; p < .05<br><br>Cannabis use frequency; self-report; CBI: 247; Control: 234; 3 months; IRR 0.55; 95% CI 0.30, 0.99; p < .05; 6 months; IRR 0.56; 95% CI 0.34, 0.91; p < .05; 12 months IRR 0.89, 95% CI 0.60, 1.31<br><br>Other drug use; self-report; CBI: 247; Control: 234; 3 month IRR 0.48; 95% CI 0.29, 0.79; p < .01; 6 months IRR 0.94 95% CI 0.60, 1.48,; 12 months; IRR 0.75, 95% CI 0.38, 1.49,<br><br>Alcohol use severity; self-report; CBI: 247; Control: 234; 3 months; IRR 0.86, 95% CI 0.48, 1.55; 6 months IRR 0.63, 95% CI 0.39, 1.01, 12 months IRR 0.82 95% CI 0.50, 1.34

c) Mental health interventions

Author (year)	Outcome domain	Outcomes
Aboody (2020)	Depression	Depressive symptoms; DASS-D; post-test; Intervention: 48; Control: 42; Mean difference −0.12; 95% CI [NR]; Cohen's d 0.42, p .012<br>(Baseline M = 1.51, Post-test M = 1.39 for intervention; Baseline M = 1.42, Post-test M = 1.39 for control)<br><br>Depressive symptoms; DASS-D; 1-month follow-up; Intervention: 48; Control: 42; Mean difference −0.11; 95% CI [NR]; Cohen's d 0.28, p .34<br>(Follow-up M = 1.40 for intervention; M = 1.46 for control)
Ampatzoglou (2024)	Depression; Anxiety	Total anxiety symptoms; Revised Child Anxiety and Depression Scale (RCADS), total anxiety scale; post-intervention; Intervention: NR; Control: NR; F = 1.16 (group×time), partial $\eta^2 = 0.026$; p .287<br><br>Total anxiety symptoms; RCADS, total anxiety scale; post-intervention; Intervention: NR; Control: NR; F = 7.37 (main effect of time), partial $\eta^2 = 0.146$; p .009<br><br>Depressive symptoms; RCADS, depression scale; post-intervention; Intervention: NR; Control: NR; F = 0.94 (group×time), partial $\eta^2 = 0.022$ p .336

		Depressive symptoms; RCADS, depression scale; post-intervention; Intervention: NR; Control: NR; F = 1.78 (main effect of time), partial $\eta^2 = 0.040$ p .188
Bauermeister (2022)	Depression; Anxiety	Depressive symptoms; Patient Health Questionnaire 8-item (PHQ-8); 4 weeks; Intervention: 135; Control: 135; Cohen's d 0.07, p .74<br><br>Anxiety symptoms; Generalised Anxiety Disorder 7-item scale (GAD-7); 4 weeks; Intervention: 135; Control: 135; Cohen's d 0.14, p .31
Egan (2021)	Anxiety; depression, suicide<br><br><br><br><br><br><br><br><br><br>Alcohol	Anxiety symptoms; (Generalized Anxiety Disorder Child Age 11-17) Intervention:120, Control: 120, 1 month follow up b -.19, 95% CI -0.42, .04, p .11, 2 month follow up b -.16, 95% CI -.40, .08, p .19,<br><br>Depression symptoms; (Patient Health Questionnaire-9 for Children Age 11-17) Intervention:120, Control: 120, 1 month follow up b -.03, 95% CI -.22, .16, p .76, 2 month follow up b - .06, 95% CI -.26, .14, p .55<br><br>Suicidal ideation; (3 items from Youth Risk Behavior Survey), 1 month follow up odds ratio 0.77, 95% CI .22, 2.87, p .70, 2 month follow up odds ratio 0.51 95% CI .12, 2.20, p .37<br><br>Suicide plan; (3 items from Youth Risk Behavior Survey), 1 month follow up odds ratio 2.15 95% CI .47, 9.74, p .32, 2 month follow up odds ratio .85, 95% CI .20, 3.70, p .83<br><br>Alcohol use frequency Intervention: 120; Control: 120; 1 month follow up 120; b −.56 95% CI −1.40, .28, p .19, 2 month follow up b −.52, 95% CI −1.38, .33, p .23<br><br>Binge drinking frequency; self-report item (YRBS); 1 month follow up; Intervention: 120; Control: 120; b = −0.39; 95% CI [−0.71, −0.06]; p .02, 2 month follow up b −.13, 95% CI −.46, .20, p .44<br><br>Binge drinking frequency (intensity-adjusted); self-report item (YRBS); 1–2 months; Intervention: 120; Control: 120; b = −0.50; 95% CI [−1.00, −0.01];<br><br>Marijuana use frequency; self-report item (YRBS);1 month follow up; Intervention: 120; Control 120: b −1.37, 95% CI −3.04, .30, p.11, 2 month follow up; b = −2.78; 95% CI [−4.49, −1.08];<br><br>Cigarette smoking frequency 1 month follow up; Intervention 120; Control 120: b .20 95% CI −.57, .96 p .62, 2 month follow up b .25 95% CI −.53, 1.03, p .53<br><br>Cigarettes smoked per day; 1 month follow up; Intervention 120; Control 120 b .30, 95% CI −3.37, 3.97, p .87, 2 month follow up b −.62 95% CI −4.37, 3.12, p .74
Glass (2022)	Depression; Suicide	Depression (CESD-R score); CESD-R scale; 12 months; Intervention: 175; Control: 171; Improvement overtime for both groups b −2.175; 95% CI −3.22, −1.23, p< .001. But change over time did not differ between groups p .653.<br><br>Suicide risk; CESD suicidal ideation items; 12 months; Intervention: 175; Control: 171; Greater improvement in intervention group b −0.799; 95% CI −1.159, −0.01, p .048

Glass (2024)	Depression;<br>Suicide;	PROMIS Paediatric Depression Scale–Short Form (8 items); 12 months; Intervention: 305; Control: 304; mean 60.8 (SD 10.2) vs mean 60.7 (SD 9.6). No significant between-group difference (group×time p .650); Both groups improved over time ($p < .001$)<br><br>Single self-report item (threatened or tried suicide in past 6 months); 12 months; Intervention: 305; Control: 304; Significant group×time interaction ($p = .006$); 12-month prevalence 13.1% vs 18.5% ($d = 0.228$)
Jeong (2024)	Depression	Depressive symptoms; CES-D (20-item); 15 days; Experimental group 1: 28; Control: 28; greater reduction in experimental group (−5.79 vs −3.54 by Day 8)<br><br>Depressive symptoms; CES-D (20-item); 15 days; Experimental group 2: 28; Control: 28; reduction in experimental group (−3.78 vs −3.54 by Day 8)<br><br>Significant difference in depressive symptoms among the groups ($F[2,810] = 3.03$, p .05), which decreased significantly at each time point ($F[2,162] = 27.83$, $p < .001$). No time x group interaction ($F[4,810] = .65$, $P = .60$).
Rushing 2021	Mental well-being<br><br><br><br><br><br><br><br><br>Alcohol	Resilience (self-reported survey, Child Youth Resilience Measure) 3 month follow up n = 833, mean treatment .03, mean control .02, mean difference .01, p .76<br><br>Positive coping (self-reported survey, Youth Coping Responses Inventory) 3 month follow up n = 833, mean treatment .07, mean control .05, mean difference .03, p .59<br><br>Self-efficacy (self-reported survey, self-efficacy scale) 3 month follow up n = 833, mean treatment .12, mean control .11, mean difference .002, p .98<br><br>Negative coping (use of alcohol) (self-reported survey, Youth Coping Responses Inventory) 3 month follow up n = 541, mean treatment .07, mean control .00, mean difference .07, p .35
Shen 2023	Depression;<br>anxiety	Depression symptoms; CDI 2:SR[S] self-report; 2 weeks; Intervention: 152; Control: 182; mean difference −0.04; 95% CI [−0.33, 0.10]; baseline mean: 1.10 vs 1.05; follow-up mean: 0.96 vs 0.92<br><br>Anxiety symptoms; GAD-7 self-report; 2 weeks; Intervention: 152; Control: 182; mean difference 0.10; 95% CI [−0.21, 0.22]; baseline mean: 1.74 vs 1.74; follow-up mean: 1.62 vs 1.52

Risk of bias assessments

**Author year**	**Random sequence generation**	**Allocation concealment**	**Blinding of participants and personnel**	**Blinding of outcome assessment**	**Incomplete outcome data**	**Selective reporting**	**Anything else, ideally prespecified**	**Overall risk of bias assessment**
Aboody 2020	Low risk.<br>Randomisation used a computer-generated list	Unclear.<br>Allocation via email; no details on concealment.	High risk.<br>Participants knew if they used the app or not.	Unclear.<br>Self report measures; no mention of assessor	Low risk.<br>only one dropout; handled with	Low risk.<br>Outcomes match protocol (OSF registration provided)	High risk.<br>One author is co-developer of the app (financial interest disclosed).	High

Author year	Random sequence generation	Allocation concealment	Blinding of participants and personnel	Blinding of outcome assessment	Incomplete outcome data	Selective reporting	Anything else, ideally prespecified	Overall risk of bias assessment
	(Randomizer.org).			blinding reported	last observation carried forward.			
Ampatzoglou 2024	Low risk. the study states participants were randomly allocated using a prespecified computer generated randomisation list	Unclear. No explicit mention of how allocation was concealed	Unclear. Described as single blind, but unclear who was blinded	Unclear Self-report measures were used. Unclear how single blinding may have affected outcome assessment.	Low risk. Follow up completion was very high ”98.9%, and missing data handled with last observation carried forward for one participant	Low risk. outcomes reported match those prespecified in the methods; no evidence of omission	Unclear. pilot nature and small sample size may introduce imprecision, but no other major bias identified	Unclear
Bauermeister 2019;	Low risk. Both studies used stratified block randomization with permuted blocks and a pseudo random number generator.	Unclear. Neither paper provided explicit details on how allocation was concealed.	High risk. Both interventions were online and participants knew their assigned condition; blinding was not feasible	High risk. Outcomes were self-reported via online surveys; no indication of blinded assessors.	Low risk. Bauermeister reported high retention (91.1% completed at least one follow up; 79.4% at 30 days, 83.3% at 60 days, 81.7% at 90 days).	Low risk. Both studies reported outcomes consistent with their protocols and objectives	High risk. Cross arm contamination occurred (25 participants exposed to intervention content in control arm), addressed by exclusion.	High risk
Bauermeister 2022	Low risk. Randomization was performed using a 1:1 allocation ratio and protocol was registered.	Unclear. No explicit description of concealment procedures.	High risk. Participants knew whether they were using the full imi app or resource only version; no blinding possible.	High risk. All outcomes were self-reported via online surveys; no blinding.	Low risk. High retention (90.4% at 4 week follow up); attrition analyses showed no systematic differences.	Low risk. Outcomes reported as per protocol; effect sizes and p values included.	Low risk. No major deviations from protocol; engagement analyses were exploratory and prespecified as secondary.	High risk

Author year	Random sequence generation	Allocation concealment	Blinding of participants and personnel	Blinding of outcome assessment	Incomplete outcome data	Selective reporting	Anything else, ideally prespecified	Overall risk of bias assessment
Bauermeister 2024	Low risk. Block randomisation with a random number allocation sequence, stratified by city, is explicitly described.	Unclear. Arm assignment used a random number generator, and participants were blinded to assignment, but concealment details at the point of enrolment are not described.	Unclear. Participants were blinded to their intervention assignment, but in practice the content differences between GC PLUS and GC TLO may have been noticeable; staff blinding is not detailed.	Unclear. Outcomes are self-reported via online surveys; no independent blinded assessors are described.	Low risk. Retention was high over 12 months, with participation rate 96.5 percent and final 12-month completion 87.3 percent; no differential attrition by arm or site is reported.	Low risk. Trial is registered on ClinicalTrials.gov (NCT03132415) and prespecified outcomes, including routine HIV and STI testing and SUS acceptability, are reported.	Low risk. The absence of a no treatment control and regional differences in service environments are acknowledged as limitations that could influence observed effects; COVID 19 disrupted Houston recruitment, potentially affecting site comparability.	Unclear risk
Bennett 2015	Low risk. Randomisation was performed via an automated algorithm, stratified by age group	Low risk. Randomisation was automated and participants were assigned immediately after baseline survey completion, minimizing investigator influence.	High risk. Participants knew whether they were viewing a tailored website or a CDC factsheet; blinding was not possible due to the nature of the intervention.	High risk. Outcomes were self-reported via online surveys; no blinding of assessors.	High risk. 50% loss to follow up at 3 months (332/661 responded). Authors noted significant differences between respondents and non-respondents (e.g., race, smoking status).	Low risk. Outcomes reported match those stated in the protocol (registered at ClinicalTrials.gov: NCT01769560).	Low risk. The study was registered on ClinicalTrials.gov (NCT01769560) and approved by an Institutional Review Board. Funding sources were disclosed, and authors stated that sponsors had no role in study design, conduct, or reporting. No evidence of early stopping, inappropriate influence, or deviations from protocol was reported.	High risk

Author year	Random sequence generation	Allocation concealment	Blinding of participants and personnel	Blinding of outcome assessment	Incomplete outcome data	Selective reporting	Anything else, ideally prespecified	Overall risk of bias assessment
Bull 2012	Low risk. Participants (networks) were randomly assigned to intervention or control using a cluster randomised design.	Unclear. The paper does not explicitly describe how allocation was concealed from recruiters or participants.	High risk. Participants knew which Facebook page they were interacting with (Just/Us vs 18 24 News), and facilitators were aware of group assignment.	Unclear. Outcomes were self-reported via online surveys; no mention of blinding of data analysts.	High risk. Retention dropped substantially at 6 months (45% intervention, 59% control), and attrition was higher among higher risk youth.	Low risk. Outcomes reported match those stated in the protocol (NCT00725959) and methods section.	Low risk. No major additional biases reported, but reliance on self-report and network recruitment could introduce bias.	High risk
Bull 2017	Low risk. The study used a cluster randomised trial design with 32 unique randomisation units assigned across clubs and years.	Unclear. The paper does not explicitly state how allocation was concealed from those enrolling participants.	High risk. Blinding was not mentioned and given the nature of the intervention (text messaging), participants and facilitators were likely aware of group assignment.	Unclear. The paper does not specify whether outcome assessors were blinded.	High risk. There was substantial attrition: Baseline: 852 participants 9 month follow up: 632 participants 21 month follow up: 387 participants Retention was lower among higher risk teens, which could bias results.	Low risk. Outcomes reported align with the stated aims and protocol.	Unclear. No major additional biases were discussed, but the authors note limitations such as small numbers of sexually active youth and potential analytic bias due to retention patterns.	High risk
Carvalho 2016	Low risk. Participants were randomly assigned to intervention or control groups using an algorithm	Unclear. The paper does not specify whether allocation was concealed from researchers or participants.	High risk. Participants were aware of whether they received an intervention or only links (control group). No mention of	High risk. Outcomes were self-reported (intention and behaviour), and there is no indication of blinding.	Low risk. Dropout analysis was conducted; differences were minimal, and reasons for dropout were reported	Low risk. Outcomes specified in the methods (intention and behaviour) were reported in the results.	Low risk. No major additional biases reported, but lack of intervention time measurement and reliance on self-report could introduce bias.	High risk

Author year	Random sequence generation	Allocation concealment	Blinding of participants and personnel	Blinding of outcome assessment	Incomplete outcome data	Selective reporting	Anything else, ideally prespecified	Overall risk of bias assessment
			personnel blinding.					
Chernick 2017	Unclear. The study reports block randomisation (1:1) but does not specify the exact method of sequence generation (e.g., computer generated random numbers).	Low risk. Authors state that allocation was concealed by a software programme, which is generally adequate to prevent foreknowledge of assignment.	High risk. Blinding was not feasible: intervention participants received text messages over 3 months, while controls received a wallet card and a standardised monologue at the ED visit. Participants and clinical staff would have been aware of group assignment.	Low risk. Outcome assessors were blinded to study arm, which mitigates detection bias for outcomes ascertained via telephone follow up and EMR review.	Unclear. Overall follow up was relatively high (88%), and an ITT approach imputed those lost to follow up as not initiated. However, loss to follow up was differential (TM 16.0% vs SR 8.2%), and there were technological failures (8 in TM received no texts). These factors could introduce bias despite ITT handling.	Low risk. The trial is registered (NCT02093884).	High risk. Intervention delivery failures and exposure variability: Only 70.8 to 70.9% of texts were successfully delivered, with >90% of failures from one carrier; 8 participants received no texts in the TM arm. Baseline imbalance in pregnancy intentions: More participants in TM were trying/wanting to be pregnant than in SR, which the authors acknowledge may have affected initiation. These factors could bias estimates of the intervention effect independently of randomisation.	

Author year	Random sequence generation	Allocation concealment	Blinding of participants and personnel	Blinding of outcome assessment	Incomplete outcome data	Selective reporting	Anything else, ideally prespecified	Overall risk of bias assessment
Chernick 2022	Low risk. Computer generated block randomisation used.	Unclear. Sequentially numbered envelopes were used, but opacity/sealing/tamper proofing not described.	High risk. Behavioural, multimedia texting intervention; blinding not described and unlikely feasible.	High risk. Outcomes assessed by self-report via web/phone follow up; assessor blinding not reported.	Low risk. Overall follow up 82.9%; differential loss (20.8% vs 13.5%) addressed with conservative ITT imputation (not initiated).	Low risk. Trial registered (NCT3866811)	High risk. Early termination due to COVID 19 and baseline imbalance (prior effective birth control use higher in intervention, p=0.02) with no adjustment in this pilot.	High risk
Chernick 2025	Low risk. Computer generated block randomisation was used.	Low risk. Sequentially numbered envelopes were used and opened after consent.	Unclear. No explicit statement about blinding participants; intervention nature makes blinding difficult. However, outcome assessors blinded.	Low risk. Outcome assessors were blinded (stated as outcome assessor blinded).	Low risk. Follow up completion was high (81.5%), and loss to follow up was analysed; no major imbalance between arms.	Low risk. Outcomes described in methods were reported in results; no evidence of omission.	Unclear. Single centre design and population characteristics may limit generalisability, but no major methodological flaws beyond those inherent to pilot trials.	Unclear risk
Christensen 2013	Low risk. The allocation sequence was automatically generated by the online data collection software (Qualtrics) with imbalanced 2:1 randomisation to anticipate technical losses.	Low risk. Researchers and staff were blind at enrolment to condition assignment, and allocation was system assigned by Qualtrics (i.e., concealed prior to enrolment decisions).	Low risk. Participants knew whether they played the SOLVE game versus wait list control; some staff were subsequently unblinded to prevent re enrolment.	Low risk. Outcomes were self-reported via online surveys (state shame immediately pre/post; UAI at 3 months). No blinding of participants is feasible for these subjective measures.	High risk. Retention = 69% at 3 months (under the CDCs 70% best evidence threshold). The authors report post allocation exclusions in the SOLVE arm when participants were unwilling/unable	Low risk. Outcomes were prespecified in the trial registry: primary outcome = change in UAI counts over three months; secondary outcome = change in shame from baseline to immediate post-test, and both were reported.	High risk. Technical barriers to receiving the intervention (download/execution problems) led to post randomisation exclusions and expected differential loss (the design used 2:1 randomisation to compensate). All outcomes rely on self-report without biological verification. These study specific	High risk

Author year	Random sequence generation	Allocation concealment	Blinding of participants and personnel	Blinding of outcome assessment	Incomplete outcome data	Selective reporting	Anything else, ideally prespecified	Overall risk of bias assessment
					to download or play the game due to hardware/software issues, and they used FIML to estimate follow up UAI change for those lost to follow up. The magnitude and nature of missingness pose risk despite mitigation.		factors introduce additional bias risk beyond the standard domains.	
Cordova 2020	Low risk. The study states that youth participants were 'randomised sequentially to either S4E or enhanced usual practice' in a '2 (group) 2 (time) small scale pilot RCT,' indicating a random allocation process was utilised.	Unclear. The paper describes randomisation but provides no explicit details about the method of allocation concealment (e.g., sealed opaque envelopes, central allocation).	High risk. Participants completed the youth app or reviewed printed materials; clinicians also used a clinician facing app in the intervention arm. Blinding in this context was not feasible and is not reported as implemented.	High risk. Outcomes were primarily self-reported surveys (e.g., substance use, sexual risk behaviours, testing uptake), and there is no indication of blinding of assessors or use of objective verification for outcomes at follow up.	Low risk. Retention at 30 day follow up was 98% (49/50), and the paper reports no differential attrition between arms.	Low risk. The trial was registered (ClinicalTrials.gov NCT03855410), and prespecified outcomes (mechanisms of change and behavioural outcomes) were reported. No key outcomes appear omitted given the studys stated pilot feasibility aims.	High risk. Potential contamination and non-random assignment of clinicians are acknowledged; clinicians provided usual practice to both arms, and clinicians were not randomised, which may bias effects toward the null or introduce imbalances. The small sample size and single site setting also raise concerns about baseline imbalances and precision typical of pilot trials.	High risk

Author year	Random sequence generation	Allocation concealment	Blinding of participants and personnel	Blinding of outcome assessment	Incomplete outcome data	Selective reporting	Anything else, ideally prespecified	Overall risk of bias assessment
Egan 2021	Low risk. Authors used permuted block randomisation with varying block sizes (2, 4, 6, 8, 10) generated using Stata.	Unclear. Randomisation was implemented in REDCap, but the paper does not explicitly state whether allocation was concealed from recruiters before assignment.	High risk. The study was non blinded; participants and researchers were aware of group assignments.	Unclear. The study was non blinded; participants and researchers were aware of group assignments.	High risk. Retention was lower than expected (T2: 67.9%, T3: 64.2%), and loss to follow up differed slightly by arm (T2: 75% control vs. 61% intervention).	Low risk. Outcomes reported align with those prespecified in the protocol (referenced in the paper).	Unclear. No major additional biases reported, but feasibility nature and reliance on self-reported measures may introduce social desirability bias.	High risk
Fang 2010;	Unclear. Randomisation stated, but method not described (e.g., no mention of computer sequence or blocks).	Unclear. No concealment mechanism described (e.g., central randomisation, sealed opaque envelopes).	Unclear Blinding not reported.	Hgh risk Behavioural, web-based programme delivered at home to mother daughter dyads, blinding not reported.	High risk. Outcomes self-reported online by girls; no assessor blinding described.	Low risk. Outcomes prespecified in Methods (psychological states, skills, parent child interactions, monitoring, family rules, substance use) are reported with statistics. Prespecified domains (individual, family, peer; substance use) reported with time Intervention statistics at 1 and 2 years.	Unclear. Intervention participants could not access post intervention measures unless all sessions were completed; may preferentially include adherent dyads, though attrition was balanced/minimal.	High risk
Fiellin 2017	Low risk. computerised single randomisation scheme in TrialDB; stratified by	Low risk. (centralised, computerised allocation via TrialDB; assignment retrieved after	High risk. (behavioural, unmasked trial; participants and staff knew allocated video game condition)	High risk. (outcomes were self-reported and assessments conducted face to face by	Low risk. (retention good and similar across arms: 6 weeks 82.7%, 12 months 81.6%; ITT	Low risk. (trial registered: ClinicalTrials.gov NCT01666496; prespecified outcomes reported; clarification of 12-	Low risk. (baseline characteristics balanced; exposure/fidelity documented; a priori	High risk

Author year	Random sequence generation	Allocation concealment	Blinding of participants and personnel	Blinding of outcome assessment	Incomplete outcome data	Selective reporting	Anything else, ideally prespecified	Overall risk of bias assessment
	gender and age; assignment under an investigator not involved with eligibility)	consent from the web-based system)		research staff; no assessor blinding reported)	primary analysis; sensitivity analyses with alternative assumptions for missing data did not change conclusions)	month primary endpoint timing occurred before any data analyses)	sample size/power calculation reported)	
Gerend 2021	Unclear. The paper states participants were randomized using a 1:1 allocation ratio ¦ and were blind to arm allocation, but does not describe the method of sequence generation	Unclear. No details are provided on the allocation concealment mechanism	Unclear risk. Although the authors state participants were blind to arm allocation, the content and emphasis of messages differed by arm (HPV focused intervention vs broader sexual health control), which likely made blinding ineffective in practice for participants; research staff delivering condition tailored messages and websites were also unlikely to be blinded. Behavioural, self-reported	High risk. Primary outcome (HPV vaccine initiation) was self-reported. The team attempted to verify all reports ¦ in I CARE, but not all could be verified. Moreover, pop up surveys asking about HPV vaccination were administered only to the intervention arm during Phase 2, while control arm pop ups did not assess vaccination, introducing differential measurement	Low risk. Retention was high and similar between arms: at 3 weeks, 93% (intervention) vs 96% (control), p = .486; at 9 months, 88% vs 91%, p = .601. For vaccine initiation, participants in the intervention who reported a dose earlier and did not complete the 9-month survey were still counted as having initiated, reducing bias from late attrition; withdrawals were minimal (n = 3) and	Low risk. The trial was registered (ClinicalTrials.gov NCT02994108) and the manuscript reports feasibility, acceptability, and initiation outcomes as described; psychosocial mediator analyses were presented as exploratory.	Unclear. Baseline characteristics were balanced (no significant differences, indicating successful randomisation). The study fell short of its recruitment goal and was under powered relative to the original target, but this affects precision rather than bias per se. A potential additional concern is the asymmetric interim measurement (intervention only vaccination pop ups) which may already be reflected under detection bias. No other clear sources of bias are evident.	High risk

Author year	Random sequence generation	Allocation concealment	Blinding of participants and personnel	Blinding of outcome assessment	Incomplete outcome data	Selective reporting	Anything else, ideally prespecified	Overall risk of bias assessment
			outcomes heighten concern.	opportunities across arms.	handling was described.			
Gilmore 2023	Low risk. Randomisation was conducted using block randomisation in Qualtrics.	Unclear No details are provided on the allocation concealment mechanism	Unclear. Principal investigator was blinded to condition assignment for the duration of the study. No explicit statement that participants or clinic staff were blinded.	Unclear. Outcomes were self-reported by participants; no mention of blinding of assessors.	Low risk. Retention was high (85.4% completed at least one follow up); attrition rates reported transparently; reasons for withdrawal described.	Low risk. Outcomes reported match those prespecified (feasibility, CRAFFT, descriptive norms); trial registered (ClinicalTrials.gov NCT03489434).	Low risk. No major additional biases noted, but study was underpowered for efficacy outcomes (acknowledged by authors).	Unclear risk
Glass 2022	Low risk. Randomisation was performed using an automated algorithm with stratification by state, presence of children, and type of college/university.	Low risk. Random assignment was automated and stratified; no indication of researcher involvement in allocation.	High risk. Participants were aware of whether they were using the myPlan app or the usual safety planning website; blinding was not possible due to the nature of the intervention	Unclear. Outcomes were self-reported via app/website; no explicit statement about blinding of outcome assessors.	Low risk. Retention was very high (98.3% at 6 months and 96.6% and 98.9% at 12 months); intention to treat analysis was used.	Low risk. Outcomes reported align with those specified in the published protocol	Low risk. Authors note limitations such as lack of geographic diversity and potential bias from baseline survey functioning as an intervention, but these do not indicate systematic bias in trial conduct	High risk
Hebert 2018	Low risk. Participants were randomised 1:1 to intervention vs control using a randomisation process;	Unclear. The paper does not explicitly state how allocation was concealed, no mention of sealed envelopes or electronic assignment.	High risk. Participants knew whether they used the app, personnel likely aware during app orientation. Providers were	Low risk. Chart review and follow up surveys were conducted by research staff; clinicians were blinded to group assignment. No	High risk. 18.1% lost to follow up at 3 months; per protocol analysis used instead of ITT; reasons for loss	Unclear. Outcomes stated in the Objective and Methods were reported in Results; no evidence of omission.	Unclear. No prespecified additional biases mentioned; potential imbalance at baseline in knowledge and current method use noted, but adjusted only descriptively	High risk

Author year	Random sequence generation	Allocation concealment	Blinding of participants and personnel	Blinding of outcome assessment	Incomplete outcome data	Selective reporting	Anything else, ideally prespecified	Overall risk of bias assessment
	described as randomised controlled trial.		blinded to group assignment, but participants were not.	indication of differential outcome assessment.	reported but no imputation.			
Hightow Weidman 2019	Unclear. Rrandomised in a 1:1 equal allocation but does not specify method (e.g., computer generated sequence)	Unclear. Concealment procedures not described	High risk. Both studies were behavioural interventions delivered online, blinding not mentioned and unlikely	High risk. Outcomes were self-reported via online surveys; no mention of blinding	Low risk. Retention 85.2% (3 months), 80.2% (6 months), 78.3% (12 months); IPW used to adjust for attrition	Low risk. Both papers report outcomes as described in methods; no evidence of selective reporting	Low risk. No major baseline imbalances reported; multiple retention strategies noted	High risk
Prokhorov 2008	Unclear. The paper states that 16 schools were randomly assigned to intervention or comparison conditions, but no details are provided on the method of sequence generation.	Unclear. No information is given on how allocation to conditions was concealed at the school or student level prior to assignment or recruitment.	High risk. Delivery differed markedly between arms (interactive CD ROM classroom sessions versus receipt of a booklet), and there is no indication that participants or school personnel were blinded to allocation.	High risk. Outcomes were self-reported via questionnaires; no blinding of assessors is described, and participants were aware of their allocation, which may influence self-report.	High risk. Only 1160 of 1574 baseline participants completed the 18 month follow up. Among baseline smokers, attrition was almost 50% in both groups. Although the authors report no differences between completers and dropouts on key characteristics, analyses were	Unclear. No protocol or registry is cited. Some planned interim follow ups at 6 and 12 months were not reported due to a school temporarily dropping out, and it is not clear whether all prespecified outcomes were reported.	High risk. Cluster randomisation by school with post assignment participant inclusion raises potential for selection imbalances at baseline; indeed, the intervention schools had a significantly higher proportion of Hispanic students than comparison schools (58.9% versus 41.6%). Outcomes were based on self-report without biochemical validation, and one schools temporary	High risk

Author year	Random sequence generation	Allocation concealment	Blinding of participants and personnel	Blinding of outcome assessment	Incomplete outcome data	Selective reporting	Anything else, ideally prespecified	Overall risk of bias assessment
					conducted among completers rather than using intention to treat.		withdrawal affected intermediate follow ups. These factors may introduce additional bias beyond the domains above.	
Jeong 2024	Low risk. Block randomisation was utilised, with severity stratification based on CES D scores. Randomisation was executed using an online randomiser, ensuring balanced allocation across the three arms.	Unclear. The method for concealing the allocation sequence was not described. There is no mention of sealed envelopes, central allocation, or equivalent procedures	High risk. Blinding was not implemented. Intervention groups received visible features such as group messaging and access to Fitbit data, whereas the control group had the app erased and no access to data, making blinding infeasible and unlikely.	High risk. No blinding of outcome assessors was reported. One primary outcome is subjective self-reported depressive symptoms via CES D, which is susceptible to assessment bias without blinding. Physical activity was recorded by device, but	High risk. Of 99 randomised participants, 84 were analysed; 5 per arm were excluded post randomisation for non-wear or missing questionnaire data. The study did not use intention to treat and analysed only completers, increasing risk of bias from	Unclear. Prespecified protocol or trial registration is not cited. The reported outcomes match those described in the methods, but without access to a protocol, selective reporting cannot be ruled out.	Low risk. Authors note several potential sources of bias: enrolment coincided with examination periods that may have influenced physical activity; the control group may have experienced a placebo effect from wearing the device; participants used two different applications rather than a single unified platform; short 2-week duration; and measurement focused	High risk

Author year	Random sequence generation	Allocation concealment	Blinding of participants and personnel	Blinding of outcome assessment	Incomplete outcome data	Selective reporting	Anything else, ideally prespecified	Overall risk of bias assessment
				the study reports combined analyses without specifying blinded assessment procedures.	differential adherence and missing data.		on step count only. These factors may introduce confounding and measurement limitations.	
Jones 2013	Low risk. Computer generated random assignments with varying block sizes (4 and 6), stratified by site.	Low risk. Assignments placed in sealed security envelopes in sequential order; research staff remained blinded until envelopes were opened later at the main office.	High risk. Participants and delivering personnel were not blinded to receipt of video versus text; intervention engagement was verified with arm specific questions after each episode/message.	Unclear. Outcomes were self-reported via ACASI, no explicit statement of blinded outcome assessment. ACASI and partner specific questioning were utilised to enhance validity, but awareness of assignment could influence self-report.	High risk. 295 participants were randomised; 238 completed follow ups at 3 and 6 months. Analyses and baseline characteristics are reported for N=238, with no intention to treat approach described and limited detail on handling of missing data by arm.	Unclear. Primary outcome (VEE) and planned statistical methods are reported, but no protocol/preregistration is cited to verify completeness of prespecified outcomes.	High risk. Authors note potential regression to the mean and the lack of a true control with equally attentive follow up in both arms, which could inflate observed reductions and obscure between arm differences.	High risk
Kaufman 2025	Low risk. Participants were randomly assigned to intervention or comparison arms using a 1:1 ratio; randomisation occurred	Unclear. Randomisation occurred within the app, but no explicit details on concealment procedures beyond automated assignment	High risk. Blinding was not possible due to the nature of mobile app content; participants knew which app they were using.	Unclear. Outcomes were self-reported via online surveys; no explicit statement about blinding of outcome assessors.	Low risk. Attrition was reported and analysed; 74% completed 1 month follow up, and intention to treat analysis was used.	Low risk. Outcomes were prespecified and reported according to CONSORT guidelines; trial registered (ClinicalTrials.gov NCT04376346).	Low risk. No major additional biases reported, but virtual design and recruitment via social media may introduce selection bias not fully addressed.	High risk

Author year	Random sequence generation	Allocation concealment	Blinding of participants and personnel	Blinding of outcome assessment	Incomplete outcome data	Selective reporting	Anything else, ideally prespecified	Overall risk of bias assessment
	automatically within the app or via Excel for non-engagers.							
Kissinger 2023	Low risk. Block randomisation generated by SAS statistical software; sealed randomisation cards prepared prior to enrolment.	Low risk. Sequentially numbered, sealed randomisation envelopes opened after baseline assessment.	High risk. Participants were aware of the intervention content (BUtiful vs. general health), and staff assisted with website access; no blinding reported.	Unclear. Outcomes were self-reported via ACASI and biological tests were objective, but no explicit statement about blinding of laboratory staff or data analysts.	Low risk. High follow up rates (6 months: 82.2% intervention vs. 83.9% control; 12 months: 80.6% vs. 77.6%) and use of multiple imputation for missing data.	Low risk. Outcomes reported match those prespecified (primary and secondary outcomes described in Methods and reported in Results).	Low risk. Financial incentives for session completion and frequent reminders could influence adherence and behaviour, but this was applied equally across arms. No other prespecified concerns noted.	High risk
Manlove 2020	Low risk. Permuted block randomisation with stratifiers for age (18, 19, 20) and race/Latinx ethnicity. Randomisation was automated immediately after the baseline survey, with no staff input.	Low risk. Participants were randomised automatically online and sent to the registration page for one of the two web-based apps. The process was system automated without staff involvement, which supports concealment up to the point of assignment.	High risk. Participants could not be blinded to content differences (reproductive health app vs general health app). Personnel interaction was minimal, but blinding was not feasible for participants in a self-led digital intervention.	High risk. Outcomes were self-reported via online surveys; there is no indication of assessor blinding beyond automated data collection. Because participants were not blinded and outcomes are subjective (e.g., recent contraceptive use), risk of detection bias is possible.	Low risk. (6 weeks): 85% intervention (565/661) and 87% control (559/643) completed. Minimal differential attrition: analyses were intention to treat with explicit handling of missing. .	Low risk. Outcomes were pre-registered (original study: ClinicalTrials.gov NCT03253783) and reported as specified (primary unprotected sex measures; secondary knowledge, attitudes, self-efficacy, intentions). Multiple testing adjustments are described; the paper notes when nominal p values did not survive correction.	Unclear. Online recruitment risk: Both studies identified and removed scammers/duplicates post randomisation (284 in original; 191 in replication). The authors acknowledge residual uncertainty about ineligible accounts and note differences in attitudes and usage that could affect external validity; internal validity is partly protected by randomisation, but residual selection/misclassifica	High risk

Author year	Random sequence generation	Allocation concealment	Blinding of participants and personnel	Blinding of outcome assessment	Incomplete outcome data	Selective reporting	Anything else, ideally prespecified	Overall risk of bias assessment
							tion risk cannot be fully excluded.	
Martinez Garcia 2023	Low risk. Participants were randomised using a computer-generated randomisation algorithm.	Unclear. The paper does not explicitly state how allocation was concealed (e.g., sealed envelopes or automated system).	High risk. Participants knew which app they were using (Crush vs. nutrition app), and blinding of personnel was not mentioned.	High risk. Outcomes were self-reported via online surveys; no mention of blinded assessors.	Unclear. Attrition was reported (retention 65% intervention, 63% control at 6 months), but no detailed handling of missing data (e.g., imputation) was described.	Low risk. Outcomes described in the methods were reported in the results; no evidence of omission.	Unclear. Potential selection bias due to social media recruitment and self-selection noted by authors but not prespecified as a risk domain.	High risk

Author year	Random sequence generation	Allocation concealment	Blinding of participants and personnel	Blinding of outcome assessment	Incomplete outcome data	Selective reporting	Anything else, ideally prespecified	Overall risk of bias assessment
Mustanski 2025	Low risk. Participants were randomised using a SMART design with equal allocation at each stage.	Unclear. The paper does not explicitly describe how allocation was concealed from staff or participants.	High risk. Blinding was not possible because interventions were behavioural and delivered via eHealth modules and videoconferencing.	Unclear. Outcomes were self-reported via online surveys; no mention of assessor blinding	Low risk. Retention was reported (84.9% at 6 months, 68.3% at 12 months), and missing data were handled using Bayesian multiple imputation and bootstrap methods.	Low risk. All pre specified outcomes (HIV testing, PrEP use, CAS, condom intentions, condom self-efficacy) were reported; trial registration noted (ClinicalTrials.gov NCT03511131).	Low risk. No major additional biases identified, but absence of a control group limits interpretation; acknowledged by authors as a design limitation.	High risk
Patel 2024	Low risk. Computer generated randomiser; block randomised by week across two clinics	High risk. Allocation by week is likely predictable; no concealment method described.	High risk. Authors state they were unable to blind responses; nature of the intervention makes blinding unlikely.	High risk. No blinding reported; providers documented who initiated discussion and most outcomes were self-reported; authors note inability to blind responses.	Unclear. Six-month chart review used; no numbers for loss to follow up or handling of missing data reported. Potential missed uptake outside the system acknowledged.	Unclear. No accessible protocol or pre-registration cited; stated primary and secondary outcomes are reported, but pre specification not documented.	High risk. Randomisation by week constitutes a cluster design, but analysis does not report cluster adjustment, risking a unit of analysis error; potential contamination and time effects; industry salary support disclosed.	High risk

Author year	Random sequence generation	Allocation concealment	Blinding of participants and personnel	Blinding of outcome assessment	Incomplete outcome data	Selective reporting	Anything else, ideally prespecified	Overall risk of bias assessment
Schinke 2005	Unclear. Randomisation is stated but the method of sequence generation is not described in any trial: Site level stratification and random division into groups are reported without method details	Unclear. None of the four reports describe any concealment procedures for group allocation	High risk. Behavioural, interactive CD ROM/computer interventions; blinding of participants and deliverers was not feasible and not reported	High risk. Self-reported outcomes collected online or by telephone; assessor blinding is not indicated: Telephone/online measurement procedures described without blinding	Low risk. Retention and handling of missing data are acceptable across trials, with checks for differential attrition and comparability of completers vs non completers: Three year trial reports modest attrition, fidelity high, and non-significant pretest differences for attritors vs non attritors	Low risk. Prespecified substance use and related psychosocial outcomes are reported with appropriate statistics and/or tables across timepoints: Longitudinal outcomes with tables and post hoc tests	Low risk. Unit of analysis concerns: cluster/site randomisation with individual level analyses acknowledged in 2004 and 2010 reports; site level tests were infeasible or yielded identical results to ignoring site	High risk
Schinke 2009	Unclear. Authors state participants were "randomly assigned" but provide no details on method.	Unclear. No description of concealment procedures.	High risk. Intervention required active mother–daughter participation; blinding was not reported.	High risk. Outcomes were self-reported online; no independent blinded assessors mentioned.	Unclear. Attrition reported (5.7% at one year, 4.2% at two years) but no details on handling missing data beyond stating leavers did not differ from completers.	Unclear. Outcomes broadly reported, but no protocol or trial registration cited to confirm prespecified outcomes.	Low risk. Baseline imbalances on two variables, incentives differed slightly for intervention arm, and reliance on self-report noted; not prespecified as risks.	High risk
Reback 2024	Low risk. The paper states: Participants	Unclear. No explicit description of how allocation was	High risk. The interventions (Text, Webapp,	Unclear. Outcomes were primarily self-reported via	Low risk. Retention was high: Follow up rates for all	Low risk. Outcomes and analyses align with the protocol (ATN	Low risk. COVID 19 disruptions led to changes in STI testing and PrEP	High risk

Author year	Random sequence generation	Allocation concealment	Blinding of participants and personnel	Blinding of outcome assessment	Incomplete outcome data	Selective reporting	Anything else, ideally prespecified	Overall risk of bias assessment
	were randomised 1:1:1 into one of three technology-based conditions ¦ (Methods, p.3959). Randomisation is clearly described, though the exact method (e.g., computer generated) is not detailed, but the phrasing strongly implies a proper random process.	concealed (e.g., sealed envelopes, central randomisation). The paper only mentions that participants were randomised after baseline procedures	Control) were behavioural and technology based, making blinding impractical. The paper does not mention any attempt to blind participants or staff	online surveys and ACASI; laboratory STI/HIV tests were objective but disrupted by COVID 19. No statement about blinding of assessors for self-reported data	conditions were 89.8% at 3 months, 80.7% at 6 months, and 83.1% at 9 month follow up . Missing data were handled using TMLE with modelling of missingness	160) and trial registration (NCT04000724). Primary and secondary outcomes are reported as planned	adherence measurement. While acknowledged, these changes could introduce bias, but the extent is Unclear.	
Reiter 2023	Low risk. The study reports randomisation via an automated process on the project website with a 1:1:1 allocation scheme	Low risk. Randomisation was automated and occurred immediately after baseline survey completion, reducing the likelihood of allocation knowledge by investigators.	High risk. Participants were aware of their assigned intervention (interactive reminders, unidirectional reminders, or standard information), and personnel interacted with participants in the interactive	High risk. Outcomes (HPV vaccination initiation and completion) were self-reported by participants; no indication of blinding for outcome assessment.	Low risk. Retention was high (90% at T2, 70% at T3, 69% at T4), and an intention to treat approach was used, categorising missing follow up as no for vaccination outcomes.	Low risk. The trial was registered (ClinicalTrials.gov: NCT04032106), and reported outcomes align with prespecified objectives (HPV vaccine initiation and completion, satisfaction)	Low risk. Study followed ethical guidelines, used strategies to minimise fraudulent accounts, and paused during COVID 19 pandemic (acknowledged as a limitation). No major additional sources of bias identified	High risk

Author year	Random sequence generation	Allocation concealment	Blinding of participants and personnel	Blinding of outcome assessment	Incomplete outcome data	Selective reporting	Anything else, ideally prespecified	Overall risk of bias assessment
			arm for tailored responses.					
Santa 2021	Unclear. The study reports a 1:1 randomised attention control design and that participants were randomised to either the control or the intervention group, but it does not describe the method used to generate the allocation sequence.	Unclear. No details are provided on allocation concealment procedures (e.g., sealed opaque envelopes, central randomisation, or app level concealment).	High risk. Participants received visibly different content: tailored, EMA responsive HIV prevention messages in the intervention arm versus non tailored, general health messages in the attention control arm. Blinding of participants and personnel was not reported and is unlikely to have been feasible given the nature of the behavioural, app delivered intervention.	High risk. Outcomes were self-reported via ecological momentary assessments in the app (e.g., sex, drug and alcohol use, stress, urges). No blinding of outcome assessors is described, and self-report without blinding can introduce differential reporting.	High risk. Of 100 enrolled, 3 were excluded due to lack of EMA data; EMA response rates decreased over time, yielding a median of 19 days of useful data and 46.3% of maximum responses. Sixteen phones were reported lost or stolen, one broken, and one misassigned; analyses relied on hierarchical models with sensitivity analysis, but substantial and potentially non-random missingness remains.	Unclear. The paper cites a ClinicalTrials.gov registration (NCT03911024) and reports on prespecified domains (sexual behaviours, substance use, urges, stress, HIV testing, PrEP). However, the manuscript does not explicitly cross reference all registered outcomes and analysis plans; without comparing to the registry entry, selective reporting cannot be ruled out.	High risk. Conflict of interest: A coauthor is the primary inventor of the platform used and receives royalties, which may introduce bias in conduct or interpretation. Attention control content: Generic health messages may affect stress and other behaviours, potentially diluting or confounding intervention effects. Pilot, convenience sampling and EMA reactivity: Small sample and potential reactivity to EMA could bias estimates.	High risk
Schnall 2022	Low risk. Authors state block randomisation	Low risk. Randomisation status was concealed from	High risk. Participants knew whether they had access	Unclear. Outcomes were self-reported via online surveys;	Low risk. Retention 80% at all follow ups; missing data	Low risk. Outcomes reported match those prespecified in the	Low risk. No major deviations from protocol reported; funding	High risk

Author year	Random sequence generation	Allocation concealment	Blinding of participants and personnel	Blinding of outcome assessment	Incomplete outcome data	Selective reporting	Anything else, ideally prespecified	Overall risk of bias assessment
	stratified by site was used; treatment assignment was predetermined and blinded.	staff and participants until completion of baseline assessment.	to the app (intervention vs delayed intervention); no blinding of personnel delivering access.	no explicit statement about blinding of data analysts or outcome assessors.	handled in mixed effects models; no evidence of differential attrition by arm.	trial protocol (referenced in Supplement 1); no evidence of omission.	source disclosed; no conflicts affecting design or analysis noted.	
Schnall 2025	Low risk. Block randomisation with random permuted sequences implemented via REDCap	Low risk. Treatment assignment was predetermined, blinded, and static throughout the trial to reduce allocation bias	High risk. No indication of participant or personnel blinding; intervention involved an app and test kits, making blinding impractical	Unclear. Outcomes were primarily self-reported HIV testing and app uploaded photos; no explicit statement about blinding of assessors	Low risk. Retention was high (overall 82.9%); reasons for withdrawal were reported; missing data handled via complete case analysis and maximum likelihood estimation	Low risk. Outcomes prespecified (primary and secondary) and reported as planned; trial registered (ClinicalTrials.gov NCT03803683)	Low risk. Independent Data Safety Monitoring Board oversight; FDA regulated study with IDE; CONSORT guidelines followed	High risk
Schwartz 2014	Low risk. Schools were stratified and randomly assigned to intervention or control	Unclear. randomisation described, but no explicit details on concealment procedures	Unclear No mention of blinding of personnel, schools or participants	Unclear. Outcomes were self-reported via web survey; no statement about blinding of assessors	Low risk. Retention rate was 83%; loss to follow up balanced between arms and tested for significance (P = .48)	Low risk. All prespecified outcomes in aims appear reported	Low risk. Cluster design noted; no major additional biases reported, but no discussion of potential contamination or imbalance beyond covariate adjustment	Unclear risk
Schwinn 2015	Low risk. Participants were randomly assigned to intervention or control arms.	Unclear. The paper does not describe how allocation was concealed.	High risk. Intervention delivery was online, and participants knew whether they received	Unclear. Outcomes were self-reported online; no indication of assessor blinding.	Low risk. Attrition was 15% at follow up and did not differ significantly by	Unclear. No protocol or trial registration mentioned; cannot confirm if all prespecified	Low risk. No additional sources of bias explicitly discussed; small sample size and short follow up noted as	High risk

Author year	Random sequence generation	Allocation concealment	Blinding of participants and personnel	Blinding of outcome assessment	Incomplete outcome data	Selective reporting	Anything else, ideally prespecified	Overall risk of bias assessment
			the programme or not. No mention of personnel blinding.		arm (Ï‡ ² = 1.93, p = .164).	outcomes were reported.	limitations but not systematic bias.	
Schwinn 2010	Unclear. Report random assignment of participants to intervention or control arms. No details on method (e.g., computer generated sequence) are provided.	Unclear. No description of concealment procedures	High risk. Intervention was web based and participants knew their group assignment; no mention of blinding of personnel.	High risk. Outcomes were self-reported online; no indication of blinding of outcome assessors.	Low risk. Attrition minimal and balanced.	Low risk. Paper reports outcomes as per stated aims (substance use and mediators); no evidence of selective reporting.	Unclear. Potential bias due to recruitment via single online platform and requirement for private computer access, limiting generalisability. No prespecified protocol mentioned.	High risk
Schwinn 2021	Low risk. The study used block randomisation (k = 10) to ensure balanced arms.	Unclear. The paper does not explicitly describe how allocation was concealed from recruiters or participants	High risk. Participants knew whether they were receiving the smartphone intervention or not; blinding was not feasible.	Unclear. Outcomes were self-reported via online surveys; no mention of blinding of assessors.	Low risk. Attrition was reported as 15% across surveys, and listwise deletion was used. Retention rates were strong enough for conclusions.	Low risk. Outcomes listed in the methods (drug use and psychosocial factors) were reported in results; no evidence of omission.	Unclear. No major additional biases reported, but authors note possible contamination in the control arm and higher acculturation in the sample compared to general Hispanic youth.	High risk
Shen 2023	Unclear Participants were randomly assigned to Project RISE or control after baseline using a randomisation procedure. No	Unclear. The paper does not explicitly describe how allocation was concealed (e.g., automated system, sealed envelopes).	High risk. Participants were aware of their assigned condition (Project RISE vs information only control), and there is no mention of	Unclear. Outcomes were self-reported online; no indication that outcome assessors were blinded (likely not applicable as assessments	High risk. Follow up completion was 59.85% overall, with differential attrition between arms (Project RISE = 55.34%, control = 64.13%).	Low risk. Outcomes and analyses were pre-registered on OSF, and all planned outcomes appear reported.	Unclear. No major additional biases reported, but reliance on self-report and short follow up may introduce bias.	High risk

Author year	Random sequence generation	Allocation concealment	Blinding of participants and personnel	Blinding of outcome assessment	Incomplete outcome data	Selective reporting	Anything else, ideally prespecified	Overall risk of bias assessment
	further details provided.		personnel blinding	were automated).	Multiple imputation was used, but attrition remains substantial.			
Starosta 2016	Unclear. Randomisation to the condom intervention versus binge drinking control is stated, but the method of sequence generation is not described.	Unclear. No details are provided on how allocation was concealed from recruiters or participants.	High risk. Participants completed distinct, behaviour specific tasks (condom use versus binge drinking) and wrote targeted essays, making blinding impracticable and not reported.	High risk. Outcomes were self-reported via online surveys and TLFB SS, with no blinding described and participants aware of assignment.	High risk. Of 422 at baseline, 216 completed the three month follow up. Missing data were mean imputed, two participants were removed at baseline and one at follow up for extensive missingness, and analyses were not conducted using intention to treat.	Unclear. No protocol or trial registration is cited. Outcomes reported align with stated aims, but pre-specification cannot be verified	Unclear. Convenience sampling from a single university, partial paper to online data entry due to technology limitations, and incentives are noted, but prespecified risk sources are not clearly detailed.	High risk
Stephenson 2020	Low risk. Randomisation list generated by a random number algorithm in Stata and incorporated	Low risk. Allocation was immediate and concealed via automated online system; researchers could	High risk. Participants knew whether they had access to the website; blinding was not feasible for this	Unclear risk. Primary outcome analysis was conducted blinded to allocation. Secondary	Low risk. Follow up rate was high (86% provided data at 3 or 6 months); multiple imputation used for missing six-	Low risk. Outcomes were prespecified; all primary and secondary outcomes reported in both publications.	Low risk. No evidence of baseline imbalance or protocol deviations affecting validity; funding source had no role in analysis or reporting.	High risk

Author year	Random sequence generation	Allocation concealment	Blinding of participants and personnel	Blinding of outcome assessment	Incomplete outcome data	Selective reporting	Anything else, ideally prespecified	Overall risk of bias assessment
	into trial software.	not influence assignment	type of intervention	outcomes were self-reported online, reducing assessor influence.	month outcomes.			
Tan 2022	Unclear.<br>Block randomisation (blocks of 6; 1:1 ratio) is reported, but the method for generating the sequence is not described	Unclear.<br>No details on concealment procedures	High risk.<br>Participants were not blinded to group assignment; personnel blinding is not described	High risk.<br>Outcomes were self-reported via online surveys by unblinded participants; no blinding of outcome assessment is described.	High risk.<br>Differential and substantial attrition across arms: cumulative attrition 27.3% (intervention) and 18.0% (control). Analyses at 6 months use reduced samples (intervention n=109; control n=123). Although ITT is stated, handling of missing data (e.g., imputation) is not detailed.	Low risk.<br>Trial registration is provided (NCT04021953)	High risk.<br>Pragmatic trial conducted after public release of the intervention; documented contamination in the control arm (13.8% had seen the series), and concurrent COVID 19 context likely affecting testing behaviours. These factors can bias effects toward the null or introduce unmeasured confounding.	High risk
Wall 2024	Low risk.<br>'An external researcher generated an unrestricted random allocation sequence using random	Unclear.<br>The paper describes sequence generation by an external researcher but does not report any procedure for concealing	High risk.<br>'Neither the participants nor the researchers involved in the study were blinded to group allocation.' Waitlist design with an active	High risk.<br>Outcomes were self-reported via web-based assessments and participants were unblinded; no blinding of assessors is described.	Unclear.<br>Follow up completion was 69% at 2 months and 64% at 6 months for the combined sample; per arm follow up	Low risk.<br>Trial was prospectively registered (ISRCTN41545712). Prespecified primary outcome (CPAQ) and secondary outcomes (CES	Unclear.<br>Authors note potential bias from the waitlist control and lack of blinding; intervention developers were among the authors but reported no direct financial income. Recruitment via social	High risk

**Author year**	**Random sequence generation**	**Allocation concealment**	**Blinding of participants and personnel**	**Blinding of outcome assessment**	**Incomplete outcome data**	**Selective reporting**	**Anything else, ideally prespecified**	**Overall risk of bias assessment**
	allocation software.'	allocation prior to assignment.	intervention increases expectation effects.		counts are not reported. Analyses used ITT mixed models under a missing at random assumption with sensitivity analyses, but differential attrition by arm cannot be verified	DC, AUDIT C, LoL) are reported with corresponding analyses.	media may introduce selection effects. These issues are acknowledged but their impact cannot be quantified from the report.	
Walton 2013	Low risk. Participants were randomly assigned using a computerised algorithm	Unclear risk The paper does not explicitly state whether allocation was concealed from recruiters or participants.	Unclear risk Participants were aware of intervention type (computer vs. therapist vs. brochure). Follow up staff were blind to condition allocation.	Unclear risk. Outcomes were self-reported, however, follow up staff were blind to condition assignment.	Low risk. Attrition was reported, reasons given (relocation/phone interviews), and analyses used ITT with GEE models. Follow up rates exceeded 80%.	Low risk. Outcomes reported match those described in the methods section; no evidence of omission.	Unclear. No major additional biases identified, but fidelity concerns noted (therapists rushed at discharge for last items).	Unclear risk
Walton 2010	Low risk. Computer generated randomisation, stratified by sex and age, with block size 21.	Low risk. Assignment used numbered sealed envelopes; participants were blinded to condition until after baseline assessment.	Unclear risk Participants were not blinded to group allocation. Follow up staff were blinded to allocation.	Unclear risk. Outcomes were self-reported by participants who were not blinded to allocation and therefore outcomes may have been susceptable to knowledge of allocation.	Low risk. Follow up rate exceeded 85 percent across arms; intention to treat analyses with GEE included all randomised participants.	Low risk. Trial registered (NCT00251212); prespecified outcomes in Methods were reported in Results and tables.	Low risk. Minor baseline imbalance in school dropout was noted and examined; authors report it was unrelated to outcomes and omitted from final models. No additional prespecified concerns identified.	Unclear risk

Author year	Random sequence generation	Allocation concealment	Blinding of participants and personnel	Blinding of outcome assessment	Incomplete outcome data	Selective reporting	Anything else, ideally prespecified	Overall risk of bias assessment
				However, follow up staff were blinded.				
Walton 2014	Low risk. Randomisation was conducted using a computer algorithm; stratified by gender and grade in the prevention trial.	Unclear. Randomisation was computer generated, but no explicit statement about concealment from recruiters.	Unclear risk Participants and interventionists were aware of the intervention type (computer vs therapist vs brochure). Follow up staff were blinded to condition assignment	Unclear risk. Self-reported outcomes by participants. Follow up staff were blinded to condition assignment.	Low risk. Follow up rates exceeded 85% across conditions; attrition analyses showed no differential drop out by condition at 6 and 12 months.	Low risk. All prespecified outcomes described in the methods (e.g., cannabis initiation, alcohol use, delinquency, and related risk factors) were reported in the results, with no evidence of selective omission or inconsistent reporting	Unclear. No major concerns noted, but fidelity differences between therapist and computer interventions could introduce variability.	Unclear risk
Whiteley 2018	Low risk. The paper states participants were randomised using a random number generator.	Unclear. No details provided on how allocation was concealed.	High risk. No mention of blinding; participants knew whether they received emails with links.	Unclear. Outcomes were self-reported via ACASI by unblinded participants; no explicit statement about assessor blinding.	Low risk. Retention was high (87% intervention; 83% control) and similar across arms; authors report no significant differences between retained and missing participants.	Low risk. No trial registration or protocol mentioned; cannot confirm if all prespecified outcomes were reported.	Low risk. Potential contamination noted (participants could share links), but authors acknowledge this as a limitation rather than a controlled factor.	

Author year	Random sequence generation	Allocation concealment	Blinding of participants and personnel	Blinding of outcome assessment	Incomplete outcome data	Selective reporting	Anything else, ideally prespecified	Overall risk of bias assessment
Ybarra 2017	Low risk. Participants were randomly assigned 1:1 to intervention or control using a computer program designed to minimise imbalance across sexual experience and identity.	Unclear. The paper states participants were blind to allocation but does not explicitly describe concealment procedures for researchers beyond minimisation algorithm.	Unclear. The paper states participants were blind to allocation but does not explicitly describe concealment procedures for researchers beyond minimisation algorithm.	Unclear. Outcomes were self-reported via online or text-based surveys; no indication of assessor blinding beyond automated data collection.	Low risk. High retention (94% at intervention end; 91% at 90 day follow up) and similar across arms; complete case analysis used.	Low risk. Outcomes reported align with protocol (condomless sex acts, abstinence, HIV testing); trial registered (NCT02113956).	Unclear. Age imbalance noted between arms; sensitivity analyses conducted, but potential residual confounding acknowledged.	Unclear risk

Author year	Random sequence generation	Allocation concealment	Blinding of participants and personnel	Blinding of outcome assessment	Incomplete outcome data	Selective reporting	Anything else, ideally prespecified	Overall risk of bias assessment
Ybarra 2021	Low risk. Randomisation was conducted at a 1:1 ratio and was stratified by sexual experience and sexual identity; the 2023 paper specifies that allocation was performed by our software program. This indicates an adequate random component in sequence generation.	Low risk. Participants were randomised after completing the baseline survey, with allocation performed centrally by software, which is consistent with adequate concealment from enrolment staff. Although the 2021 paper notes participants, but not researchers, were blind, the central, software driven assignment after baseline supports concealment.	High risk. Behavioural, text message interventions inherently limit blinding. The authors state participants (but not researchers) were blind to arm allocation, and they attention matched message volume and added two days of pregnancy prevention content in control to help blind this arm. Nevertheless, content differences and unblinded personnel (who monitored. Text Buddy conversations in the intervention only) mean performance bias risk is high.	High risk. Outcomes were self-reported via online or text surveys, and participants could plausibly infer their assignment from message content despite efforts to mask. There was no independent blinded assessor, and survey mode varied (text vs online) due to funding changes, with acknowledged wording/time frame differences (models adjusted for survey mode). This leaves substantial risk of measurement bias.	Low risk. Short term: 84% completed the intervention end survey (799/948), with similar completion by arm and sensitivity analyses showing conclusions unchanged when excluding decline to answer responses. Long term: mixed effects models used likelihood-based estimation with all available data (no direct imputation); 12 month follow up included 372/473 intervention and 394/475 control (see CONSORT), which is acceptable and broadly balanced.	Low risk. The trial was registered (ClinicalTrials.gov NCT03029962) and the papers report the stated primary outcomes (condom protected sex events; contraception use other than condoms; abstinence; pregnancy), plus intentions at short term.	Unclear. Several design features could introduce bias but were partially mitigated: Survey mode changed mid trial owing to funding uncertainties; wording/time frame differences between text and online were acknowledged and adjusted for in models. Incentives varied by wave and mode, which may influence response propensity. The intervention included interactive components (e.g., Text Buddy, badges, G2Genie) not present in control. While intentional, this also introduces differential co intervention beyond content alone. Given these factors and mitigation steps, I judge Unclear. risk rather than definitively high or low.	High risk

**Author year**	**Random sequence generation**	**Allocation concealment**	**Blinding of participants and personnel**	**Blinding of outcome assessment**	**Incomplete outcome data**	**Selective reporting**	**Anything else, ideally prespecified**	**Overall risk of bias assessment**
Biello 2021	Low risk. Participants were randomised 2:1 to intervention vs. control arms. Randomisation procedures are described	Unclear. The paper does not specify how allocation was concealed or whether concealment was ensured.	High risk. Participants knew whether they received the mobile app or pamphlets. Personnel were also aware of allocation. Blinding was not possible due to the nature of the intervention.	Unclear. Outcomes were primarily self- reported (HIV/STI testing, acceptability) and supplemented by order tracking and lab reports. The paper does not state whether outcome assessors were blinded.	Low risk. Follow- up completion was high: 84% at 3 months, 79% at 6 months, and 89% completed at least one follow- up. Attrition was reported transparently.	Low risk. Outcomes described in the methods (testing rates, acceptability, ordering, return rates, positivity) were reported in the results. No evidence of selective reporting.	Unclear. As pilot randomised controlled trials, the focus was feasibility and acceptability. No prespecified statistical comparisons of intervention vs. control effectiveness were reported, which limits interpretation.	
Biello 2022	Unclear. Randomised by site to intervention or comparison is stated, but the method of sequence generation is not described.	Unclear. No details are provided on how allocation was concealed.	High risk. Participants received a mobile app versus standard of care. Blinding was not reported and is unlikely to have been feasible.	High risk. Primary outcomes were self- reported at follow- up with no blinding described.	High risk. Differential follow- up shown in CONSORT: intervention arm had losses to follow- up, whereas control showed complete follow- up in the diagram. Handling of missing outcome data is not detailed, despite an intention- to- tr eat statement.	Low risk. Trial was registered (ClinicalTrials.gov NCT03179319). Reported outcomes align with the protocol aims: feasibility, acceptability, HIV testing, and PrEP initiation, with prespecified usability threshold and planned analyses presented.	Low risk. Paradata for app usage were missing for seven participants due to a storage error that could influence feasibility outcomes, although a worst- case sensitivity analysis was conducted. Pilot design with small sample and urban- only sites may also introduce concerns not fully quantifiable.	High risk

Author year	Random sequence generation	Allocation concealment	Blinding of participants and personnel	Blinding of outcome assessment	Incomplete outcome data	Selective reporting	Anything else, ideally prespecified	Overall risk of bias assessment
Biello 2025	Low risk. Randomisation was computer generated with 1:1:1 allocation, stratified by site and age.	Unclear. The method for concealing the random sequence prior to assignment is not described.	High risk. The trial did not use masking; participants and staff were aware of assignment to app vs standard of care	High risk. Primary outcomes were self- reported by participants via self- administered, web- based assessments with no blinding indicated.	Low risk. Retention was high (95% at 3 months; 92% at 6 months), missing data were under 10%, and complete- case analysis was used.	Low risk. The protocol was registered (ClinicalTrials.gov NCT03965221), prespecified primary outcomes were reported, and the shift of the primary endpoint to 6 months was documented and approved by the monitoring committee and IRB.	Low risk. Pandemic- related procedural changes (remote enrolment; national expansion; shortened follow- up for a subset) and reliance on self- report could introduce bias, although changes were documented and approvals obtained.	High risk
Mustanski 2013;	Low risk. Randomisation was computerised and stratified by race using an algorithm.	Unclear. Allocation concealment beyond computer algorithm was not described.	Low risk. Trial was double blinded so neither participants nor tracking staff knew allocation.	Unclear. Outcomes were self-reported online; no explicit mention of assessor blinding.	Low risk. Retention was high (80.3% KIU!; 94.2% control) and attrition analyses showed no demographic differences.	Low risk. Outcomes align with protocol registered on ClinicalTrials.gov; no evidence of selective reporting.	Unclear risk. Small sample size and controlled conditions; self-report measures and participant incentives may limit generalisability.	Unclear risk
Mustanski 2018;	Low risk. describe computerised randomisation using permuted blocks and stratification by race and site.	Unclear. Randomisation was automated via an eHealth platform, but concealment procedures (e.g., whether allocation was hidden from enrolment staff) are not fully detailed.	Low risk. Both trials were described as double blinded; participants and staff did not know which arm was under evaluation.	Low risk. Laboratory testing of GC/CT was objective and independent.	Low risk. Retention was high. Analyses were complete case; no imputation, but sensitivity analyses showed no bias.	Low risk. Protocols were published, and primary outcomes were reported	Low risk	Unclear risk
Glass 2024	Unclear Procedures not reported	Unclear The paper does not explicitly describe	High risk Participants knew whether	Unclear Outcomes were self-reported via	Low risk Retention was high (87% at 12	Low risk Outcomes reported match those stated	Low risk. No major additional biases identified, but	High risk

Author year	Random sequence generation	Allocation concealment	Blinding of participants and personnel	Blinding of outcome assessment	Incomplete outcome data	Selective reporting	Anything else, ideally prespecified	Overall risk of bias assessment
		the method used to conceal allocation after randomisation	they were using the myPlan Teen app or the usual care website; blinding was not feasible.	surveys; no mention of blinding of assessors.	months), and intention-to-treat analysis was used.	in the methods (safety behaviours, DV, depression, suicide behaviour).	the use of a convenience sample and online recruitment during COVID-19 may introduce contextual bias.	
Mason 2015	Low risk Randomisation was completed using a random number table generated by an online statistical computing program (GraphPad) and blocked randomisation to create equal numbers in treatment and control groups.	Unclear The paper does not describe how allocation was concealed after sequence generation	High risk Participants were aware of the intervention content (text messages about smoking vs general health), and there is no indication of personnel blinding.	Unclear Outcomes were self-reported via text and surveys; no explicit statement about blinding of assessors.	Low risk All participants completed baseline and follow-up assessments at 1, 3, and 6 months; completion rates were high (80.4%). No major differential attrition reported	Low risk Outcomes reported match those described in the methods section (smoking behaviour, intentions, readiness, peer network measures). No evidence of omitted prespecified outcomes.	Unclear No major additional biases identified, but small sample size and preliminary nature of the trial may limit generalisability.	High risk
Schwinn 2015	Low risk Participants were randomly assigned to intervention or control arms	unclear The paper does not describe how allocation was concealed	High risk Intervention was delivered online and participants knew their group assignment; no mention of personnel blinding.	Unclear Outcomes were self- reported online; no explicit statement about blinding of assessors	Low risk Attrition was 15% and did not differ significantly by arm (p = .164); reasons for loss to follow- up not detailed but overall retention was high	Low risk Outcomes reported match those described in the methods; no evidence of selective reporting.	Low risk No major additional biases identified, but small sample size and short follow- up noted as limitations	
Rushing 2021	Low risk Participants were	Unclear risk No details on concealment	High risk Participants and staff were aware	High risk Outcomes were self-reported via	High risk 833 analysed out of 1,044	High risk Trial was registered retrospectively,	Unclear risk Short washout period in crossover design	High risk

Author year	Random sequence generation	Allocation concealment	Blinding of participants and personnel	Blinding of outcome assessment	Incomplete outcome data	Selective reporting	Anything else, ideally prespecified	Overall risk of bias assessment
	randomised using a computer-generated sequence (Excel)	procedures; it's not clear if allocation was hidden from recruiters	of group assignment because message content differed	online surveys; no blinding of assessors or participants.	randomised; exclusions after randomisation and limited explanation raise concern	increasing risk of outcome switching.	and potential conflicts of interest (authors employed by intervention developer) noted, but impact uncertain	
Mason 2016	Low risk<br>Participants were randomised using a blocked design to create equal allocation between intervention and control groups	Unclear<br>The paper does not describe how allocation was concealed	Unclear<br>Blinding of participants or study staff is not reported in the paper	Unclear<br>Outcomes were self- reported online; no explicit statement about blinding of assessors	High risk<br>172 of 200 randomised participants analysed. No analyses undertaken to assess the impact of missing outcome data.	Unclear<br>No protocol or trial registration is cited. Outcomes reported align with stated aims, but pre-specification cannot be verified	Low risk<br>No major additional biases identified, but small sample size and preliminary nature of the trial may limit generalisability.	High risk
